# Using Tumor-Infiltrating Immune Cells and a ceRNA Network Model to Construct a Prognostic Analysis Model of Thyroid Carcinoma

**DOI:** 10.3389/fonc.2021.658165

**Published:** 2021-06-01

**Authors:** Fan Zhang, Xiaohui Yu, Zheyu Lin, Xichang Wang, Tiantian Gao, Di Teng, Weiping Teng

**Affiliations:** Department of Endocrinology and Metabolism, Institute of Endocrinology, National Health Commission Key Laboratory of Diagnosis and Treatment of Thyroid Diseases, The First Hospital of China Medical University, Shenyang, China

**Keywords:** thyroid carcinoma, ceRNA network, immune infiltration, prognosis, nomogram

## Abstract

Thyroid carcinoma is a solid malignant tumor that has had a fast-growing incidence in recent years. Our research used thyroid carcinoma gene expression profiling from TCGA (The Cancer Genome Atlas) database to identify differentially expressed ceRNAs. Using the gene expression profiling from 502 carcinoma thyroid tissues and 58 normal thyroid tissues from the TCGA database, we established the thyroid carcinoma-specific competitive endogenous RNA (ceRNA) network and found nine overall survival (OS)-associated genes (*PRDM1, TGFBR3, E2F1, FGF1, ADAM12, ALPL, RET, AL928654.2, AC128688.2*). We quantified the proportions of immune cells using the algorithm “CIBERSORT”, found three OS-associated immune cells (memory B cells, M0 macrophages, and activated dendritic cells), and established a thyroid carcinoma-specific immune cell network based on that. The good reliabilities AUC (area under the curve) of 10-year survival (0.955, 0.944, respectively) were accessed from the nomograms of genes and immune cells. Subsequently, by conducting co-expression analyses, we found a potential regulation network among ceRNAs and immune cells. Besides, we found that *ALPL* (alkaline phosphatase) and hsa-miR-204-5p were significantly correlated and that *ALPL* was related to activated dendritic cells. We took advantage of multi-dimensional databases to verify our discovery. Besides, immunohistochemistry (IHC) assays were conducted to detect the expression of a dendritic cell marker (CD11c) and ALPL in thyroid carcinoma (TC) and paracancerous tissues. In summary, our study found a potential mechanism in which hsa-miR-204-5p regulated *ALPL* in activated dendritic cells, which may allow them to play a critical role in thyroid carcinoma. These findings provide potential prognostic biomarkers and therapeutic targets for thyroid carcinoma.

## Introduction

The incidence of thyroid cancer is on the rise. This solid malignant tumor has had a fast-growing incidence in recent years. This disease is projected to become the fourth leading type of cancer across the globe. Thyroid cancer is responsible for 567,000 cases worldwide, ranking ninth in place for incidence (1). From 1990 to 2013, the global age-standardized incidence rate of thyroid cancer increased by 20% (2). In China, approximately 201,000 people develop thyroid cancer per year, and the incidence rate is approximately 14.6 per 100,000 people (3). Worldwide, it has become one of the main diseases threatening the health of people. Although thyroid cancer mortality is much lower than the incidence rate, the mortality rate of thyroid cancer has increased significantly (0.7%, p <0.001 per year) since the late 1980s (4–7). These trends are consistent with the prevalence of certain risk factors (including obesity and non-frequent smoking) over time (8). Lim et al. (9) used SEER data from 1974–2013 to compare trends in thyroid cancer morbidity and mortality by demographic and tumor characteristics at the time of diagnosis. They found that among patients diagnosed with thyroid cancer in the United States from 1974 to 2013, the total incidence of thyroid cancer increased by 3% per year. This represents an average relative annual increase in mortality of 1.1% per year (10). Therefore, although thyroid cancer is relatively inert, the poor prognosis represented by the rising mortality cannot be underestimated.

Molecular and cellular biomarkers play an important role in the prognosis of thyroid cancer in pathological diagnosis and prognosis (11). Non-coding genes can regulate gene expression to a certain degree. The expression of mRNA and microRNA (miRNA) can be regulated by lncRNA (long non-coding RNA) through changes in chromatin modification and mRNA stability at the transcriptional level (12). MicroRNA regulates gene expression after transcription (13). The competitive endogenous RNA (ceRNA) network is a transcriptional regulatory network at the molecular level, consisting of lncRNA, miRNA, and mRNA. More and more studies have shown that ceRNA networks regulate the transcription of oncogenes and tumor suppressor genes, regulate the interaction between proteins and genes, and control biological behaviors, such as tumor invasion and metastasis (14). Both tumor cells and invasive immune cells are involved in tumorigenesis and tumor progression (15). At the cellular level, it has been proven that assessing the degree and type of tumor-infiltrating immune cells is of great importance for predicting metastasis and mortality (16, 17). However, no joint network has been established to predict the prognosis of thyroid cancer. Therefore, a deeper understanding of thyroid cancer tumor-infiltrating immune cells and ceRNA networks is needed.

In this study, we screened the differential expression of lncRNA, miRNA, and mRNA in thyroid tumor and normal thyroid tissue using the TCGA database and established a ceRNA network based on gene expression profiles. Meanwhile, we used CIBERSORT (cell type identification by estimating relative subsets of RNA transcripts) (17) to quantify the proportion of immune cells in all samples. Based on the ceRNA network and immune cell analyses, two nomograms were established to predict the prognosis of thyroid carcinoma. Overall, we found a relationship between the thyroid carcinoma OS (overall survival)-specific ceRNA network and immune cells, which may play a critical role in thyroid carcinoma.

## Materials and Methods

### Data Collection and Differential Gene Expression Analysis

The relevant data provided by TCGA are publicly available. Expression profiles of thyroid carcinoma were downloaded from TCGA’s (https://portal.gdc.cancer.gov) database, including mRNA, lncRNA, and miRNA. The exclusion criteria were set as follows: 1) histological diagnosis was not thyroid carcinoma and 2) samples were without complete RNA sequencing data. On the whole, we included 502 patients who had demographic information and survival endpoint information. The HTseq-count profiles of 560 samples were comprised of 502 thyroid carcinoma tissues and 58 normal thyroid tissues. We screened the thyroid carcinoma and paracancerous-specific expression genes and conducted an expression differences analysis of each RNA between thyroid carcinoma and paracancerous tissues by using the DEseq2. With a false discovery rate (FDR) adjusted p-value < 0.05, a log (fold-change) > 1.0 or < -1.0 was defined as a downregulated or upregulated gene, respectively. Volcano plots and heat map analyses of differentially expressed mRNAs, lncRNAs, and miRNAs were performed using the plot function and the pheatmap package in R, respectively.

### Construction of the ceRNA Network

Before the primary statistical analysis, miRNA-lncRNA interactions were predicted by miRcode (http://www.mircode.org/) (18), and miRNA-mRNA interactions were predicted by miRTarBase (http://mirtarbase.cuhk.edu.cn) (19). By screening the miRNAs that could regulate both lncRNA and mRNA, the significant results in hypergeometric distribution testing (p < 0.01) and correlation analysis (p < 0.01) were selected for visualization of the ceRNA network using Cytoscape v.3.8.0 (20).

### Functional Enrichment Analysis of mRNAs in the ceRNA Network

To further study the function of the predicted signature, the Kyoto Encyclopedia of Genes and Genomes (KEGG) and Gene Ontology (GO) enrichment analyses for differentially expressed mRNAs in the ceRNA network were performed using the clusterProfiler package. The cellular component (CC), molecular function (MF), and biological process (BP) terms were evaluated. Significance was defined as p < 0.05.

### Survival Analysis and Nomograms in the ceRNA Network

To identify OS-associated lncRNAs, miRNAs, and mRNAs in the ceRNA network, we used Kaplan-Meier survival analysis curves (p < 0.05) and a univariate Cox proportional hazards model to evaluate the prognostic value of all biomarkers. Then, the OS-related RNAs were entered into the stepwise multivariate Cox regression model to construct thyroid carcinoma prognosis-associated signatures. The Lasso regression was employed to ensure that the multivariate models were not overfitting. The prognostic risk score for predicting OS was conducted as follows:

$$Gene\;Risk\;Score=\;\sum_{n=1}^{k}C_{k}*G_{k}$$

in where n is the number of selected genes, C_k_ is the coefficient of gene k, and G_k_ is the expression level of gene k. All patients were classified into high- and low-risk groups with a median risk score. A Kaplan-Meier analysis was performed to assess the difference in overall survival between high- and low-risk groups. Eventually, we built a nomogram based on the multifactor models to predict the prognosis of patients with thyroid carcinoma. The receiver operating characteristic curves (ROC) and calibration curves were utilized to assess the discrimination and accuracy of the nomogram. The AUC value was calculated from the ROC curve.

### CIBERSORT Estimation

To estimate the proportion of infiltrating immune cells, standard annotated gene expression data were downloaded from the CIBERSORT web portal (http://cibersort.stanford.edu/), with LM22 signature and 1,000 permutations applied to the algorithm. Only cases with CIBERSORT p < 0.05 were considered eligible for subsequent Kaplan-Meier analysis.

### Survival Analysis and Nomograms of Key Members of the Immune Cells

To identify OS-associated immune cells, we used Kaplan-Meier survival analysis (p < 0.05) and Cox regression to detect the prognosis-associated immune cell types. Then, the OS-related immune cells were entered into the stepwise multivariate Cox regression model to construct thyroid carcinoma prognosis-associated signatures. The Lasso regression was employed to ensure that the multivariate models were not overfitting. The prognostic risk score for predicting OS was conducted as follows:

$$Immune\;Cells\;Risk\;Score=\;\sum_{n=1}^{k}D_{k}*I_{k}$$

in where n is the number of selected genes, D_k_ is the coefficient of immune cell k, and I_k_ is the expression level of immune cell k. All patients were classified into high- and low-risk groups with a median risk score. A Kaplan-Meier analysis was performed to assess the difference in OS between high and low risk groups. Eventually, we built a nomogram based on the multifactor models to predict the prognosis of patients with thyroid carcinoma. The receiver ROC and calibration curves were utilized to assess the discrimination and accuracy of the nomogram. The AUC value was calculated from the ROC curve.

### Immunohistochemistry

Paraffin-embedded, formalin-fixed thyroid carcinoma (TC) and paracancerous tissue specimens were used for immunohistochemistry (IHC). Sections were incubated overnight in a humidified container at 4°C with primary antibodies against ALPL (alkaline phosphatase) (1:100, ab109185, Abcam) and CD11c (1:100, ab52632, Abcam). After washing three times, tissue sections were incubated for 1 h at room temperature with the secondary antibody conjugated with streptavidin-horseradish peroxidase. The slides were stained with 3,3’-diaminobenzidine tetrahydrochloride (DAB), and the nuclei were counterstained with hematoxylin. Immunostaining on each slide was assessed by experienced pathologists to examine the percentage of ALPL- or CD11c-positive tumor cells and presented as a histochemistry score (H-score).

### Multi-dimensional Validation

To minimize bias, multiple databases including the Oncomine (21), The Human Protein Atlas (22), UALCAN (23), Genotype-Tissue Expression (GTEx) (24), Cancer Cell Line Encyclopedia (CCLE) (25), and STRING (26) were used to detect the gene and protein expression levels of key biomarkers at the tissue and cellular levels.

### Statistical Analysis

**Figure 1:**
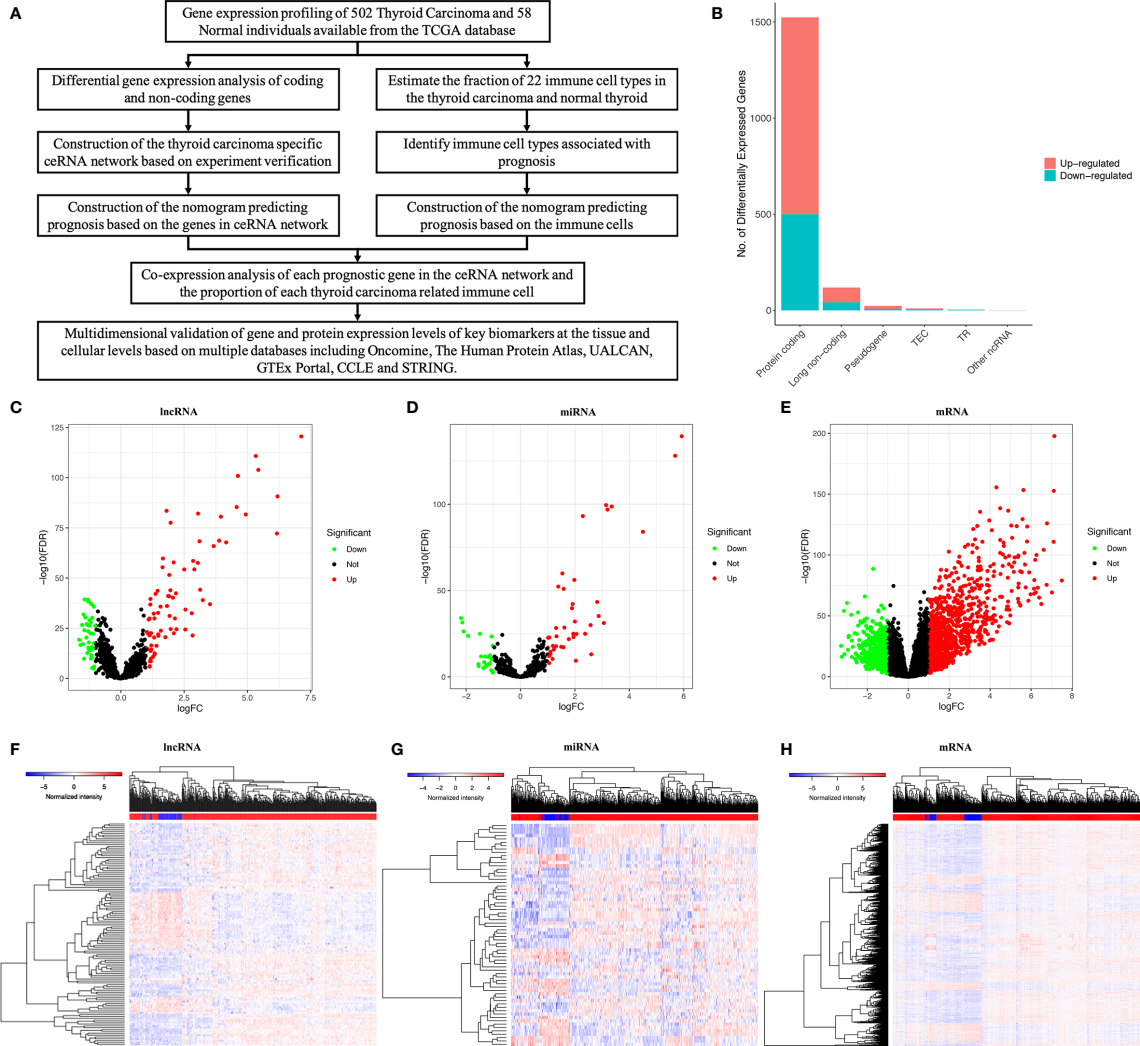
The whole analytical process of this study **(A)**; total of lncRNAs, mRNAs and miRNAs from TCGA (The Cancer Genome Atlas) database **(B)**; differentially expressed lncRNAs; **(C, F)**; differentially expressed miRNAs **(D, G)**; differentially expressed mRNAs **(E, H)**.

Only a two-sided p-value < 0.05 was considered to be of statistical significance. All statistical analyses were implemented with R version 4.0.2 software (Institute for Statistics and Mathematics, Vienna, Austria; www.r-project.org) [Package: GDCRNATools (27), edgeR, ggplot2, limma, rms, glmnet, preprocessCore, survival, survminer, timeROC, corrplot, pheatmap, vioplot, ggpubr, ggExtra, clusterProfiler]. The whole analytical process of this study is shown in **Figure 1A**.

## Results

### Identification of Significant Differential Genes

We used a log (fold-change) > 1.0 or < -1.0 and FDR < 0.05 as cutoffs to identify differential RNA profiles. The baseline characteristics of all the patients available from the TCGA are described in **Supplementary Table 1**. In a total of 60483 lncRNAs and mRNAs, and 2,588 miRNAs from the TCGA database (**Figure 1B**), there were 119 differentially expressed lncRNAs (42 downregulated and 77 upregulated) (**Figures 1C, F**), 66 differentially expressed miRNAs (24 downregulated and 42 upregulated) (**Figures 1D, G**), and 1524 differentially expressed protein-coding genes (500 downregulated and 1024 upregulated) (**Figures 1E, H**). **Supplementary Table 2** summarizes the top 10 downregulated and top 10 upregulated genes in the differential gene analysis.

### Construction of the ceRNA Network

**Figure 2:**
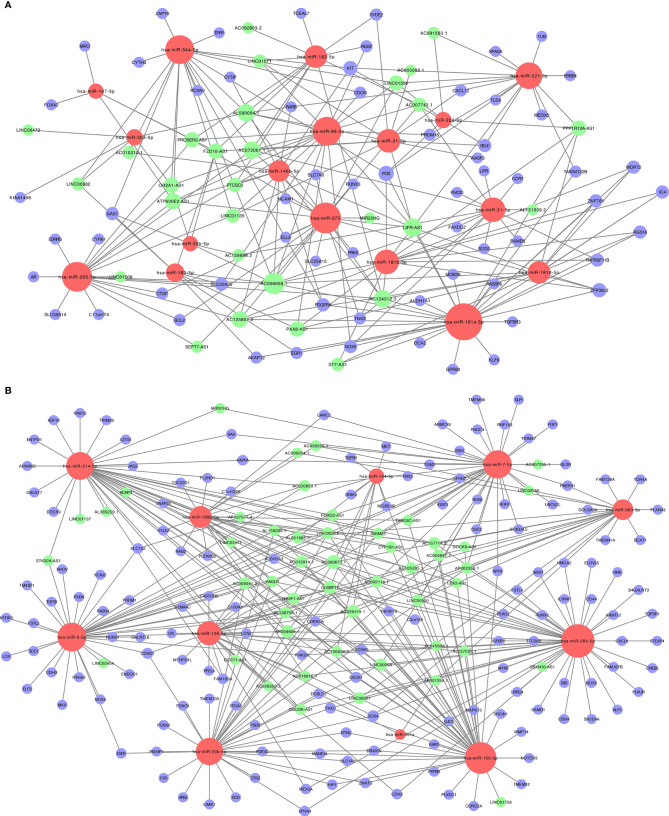
The lncRNA-miRNA-mRNA ceRNA (competitive endogenous RNA) networks. **(A)** Downregulated lncRNAs (green) and mRNAs (purple) and upregulated miRNAs (red); **(B)** upregulated lncRNAs (green) and mRNAs (purple) and downregulated miRNAs (red).

We constructed a ceRNA network, which included 317 genes, based on the interactions of 281 lncRNA–miRNA pairs and 285 miRNA–mRNA pairs. Because of the negative regulatory relationship between miRNA and lncRNA and mRNA, in the ceRNA network with high miRNA expression, lncRNAs and mRNAs had low expression (**Figure 2A**). In contrast, where miRNAs had a low expression, lncRNAs and mRNAs had high expression (**Figure 2B**).

### Functional Enrichment Analysis of mRNAs in the ceRNA Network

The functional role of mRNAs was assessed using the clusterProfiler package (**Supplementary Figures 3A–D**). The results showed that differentially expressed mRNAs in the ceRNA network were associated with BP terms, including gland development, urogenital system development, response to steroid hormones, lymphocyte differentiation, and T cell differentiation. These genes were significantly enriched in the AGE-RAGE signaling pathway in diabetic complications, colorectal cancer, endocrine resistance, p53 signaling pathway, and Th17 cell differentiation.

### Survival Analysis

**Figure 3:**
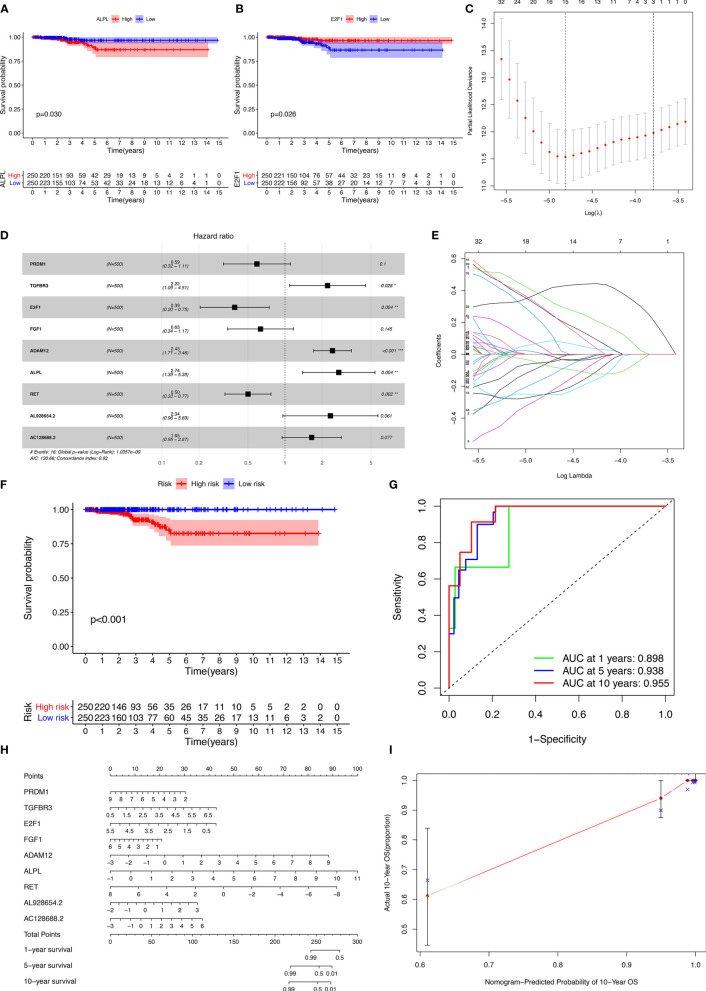
Kaplan-Meier analysis of different genes **(A, B)**; the results of the Lasso regression **(C–E)**; ROC (receiver operating characteristic curves) and the calibration curves **(F, G)**; nomogram based on the model **(H)**; calibration curves of the nomogram **(I)**.

The miRNAs regulated by both lncRNAs and mRNAs in hypergeometric distribution testing and correlation analysis are shown in **Supplementary Table 3**. We used Cox regression, Kaplan-Meier, and the log-rank test (p < 0.05) to examine the relationship between biomarkers in the thyroid carcinoma ceRNA network and prognosis. Forty-four genes were found to be significant in the Kaplan-Meier analysis, including *ALPL* (p = 0.03) and *E2F1* (p = 0.026) (**Figures 3A, B** and **Supplementary Figure 2**).

The results of the Lasso regression revealed that fifteen genes were essential for modeling (**Figure 3C**). However, only nine potential prognosis-related biomarkers were key members of the ceRNA network (**Table 1**) and were integrated into a new multivariable model (**Figures 3D, E**). Furthermore, the ROC and calibration curves suggested an acceptable accuracy (AUC of 1-year survival: 0.898; AUC of 5-year survival: 0.983; AUC of 10-year survival: 0.955) (**Figures 3F, G**). The nomogram was constructed based on the model (**Figure 3H**) and the discrimination of the nomogram (**Figure 3I**). The genes risk score for prognosis was calculated as follows: Genes Risk Score = (-0.5257 × *PRDM1*) + (0.7967 × *TGFBR3*) + (-0.94 × *E2F1*) + (-0.4575 × *FGF1*) + (0.8887 × *ADAM12*) + (1.0064 × *ALPL*) + (-0.6918 × *RET*) + (0.8499 × *AL928654.2*) + (0.4993 × *AC128688.2*).

**Table 1:** Cox proportional hazards regression model including the key members of the ceRNA network for overall survival in patients with thyroid carcinoma.

Gene	Hazard ratio	95%CI	p value
*PRDM1*	0.59	(0.32-1.11)	0.1
*TGFBR3*	2.22	(1.09-4.51)	0.028*
*E2F1*	0.39	(0.20-0.75)	0.004**
*FGF1*	0.63	(0.34-1.17)	0.145
*ADAM12*	2.43	(1.71-3.46)	< 0.001***
*ALPL*	2.74	(1.39-5.39)	0.004**
*RET*	0.50	(0.33-5.69)	0.002**
*AL928654.2*	2.34	(0.96-5.69)	0.061
*AC128688.2*	1.65	(0.95-2.87)	0.077

ceRNAs, competing endogenous RNAs; CI, confidence interval. *p < 0.05; **p < 0.01; ***p < 0.001; In the variable selection process, first of all, the univariate Cox models including all members of the ceRNA network were used to select potential prognostic genes. At the same time, the Lasso regression was performed based on all members of the ceRNA network. The results of the Lasso regression (**Figures 3C–E**) suggested that the 9 genes were essential to modeling and ensuring not overfitting of the model. Eventually, the univariate Cox model is shown in this table only including 9 genes filtrating by the Lasso regression (The Cox model of immune cells was constructed in the same way).

### Composition of the Immune Cells in Thyroid Carcinoma

**Figure 4:**
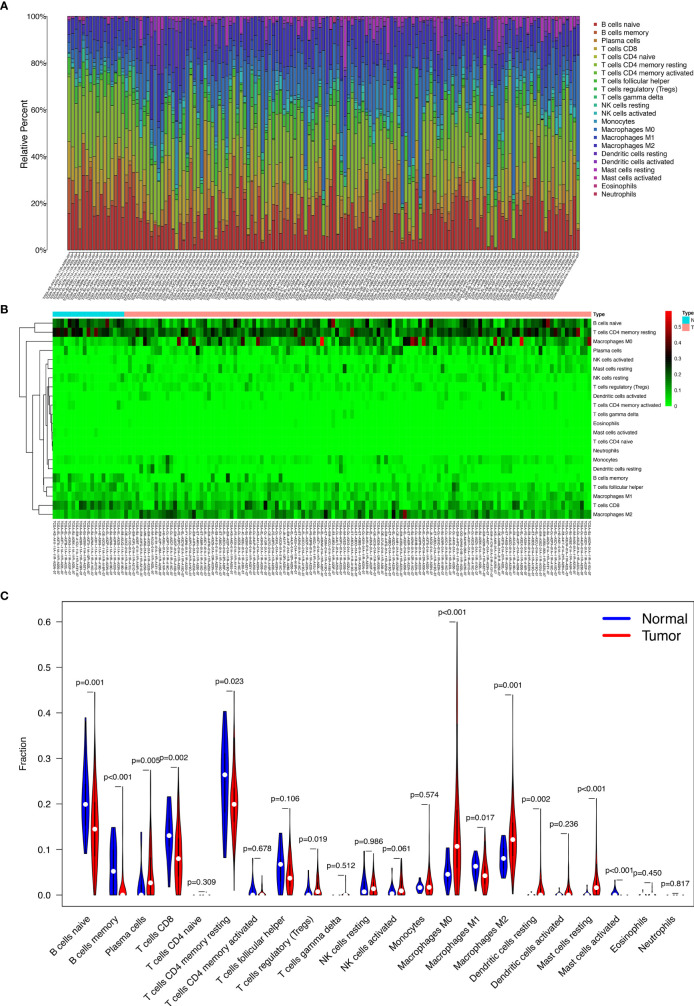
The proportion of the 22 immune cells detected by the CIBERSORT algorithm **(A, B)**; the results of the Wilcoxon rank-sum test **(C)**.

**Figures 4A, B** display the proportions of the 22 immune cells detected by the CIBERSORT algorithm. The violin plot (**Figure 4C**) depicts the results of the Wilcoxon rank-sum test. The results showed that the fraction of naive B cells (p = 0.001), memory B cells (p < 0.001), plasma cells (p = 0.005), CD8 T cells (p = 0.002), resting memory CD4 T cells (p = 0.023), regulatory T cells (Tregs) (p = 0.019), M0 macrophages (p < 0.001), M1 macrophages (p = 0.017), M2 macrophages (p = 0.001), resting dendritic cells (p = 0.002), resting mast cells (p < 0.001), and activated mast cells (p < 0.001) were significantly different between the thyroid carcinoma group and normal thyroid group.

### Clinic Correlation and Nomogram of the Immune Cells

**Figure 5:**
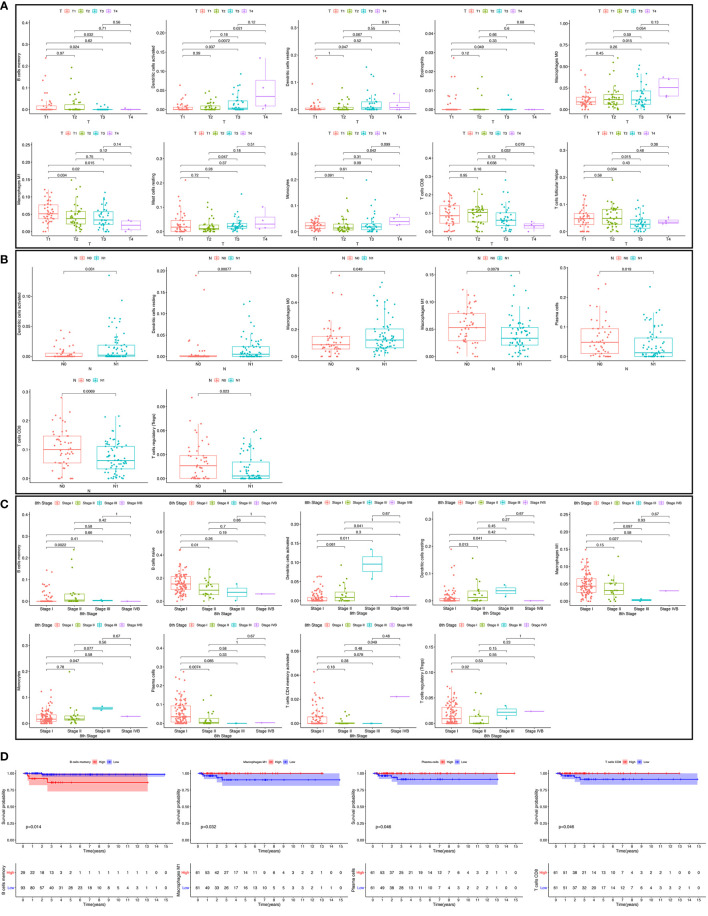
The fraction of immune cells in the different T stages **(A)**. The fraction of immune cells in different lymph node metastasis situations **(B)**; the fraction of immune cells in four tumor stages **(C)**; the relationship between immune cell fraction and overall survival (OS) **(D)**.

We used the non-parameter test and Kaplan-Meier survival analysis to examine the association between the different fractions of immune cell subtypes and prognosis. The fraction of memory B cells, activated dendritic cells, resting dendritic cells, eosinophils, M0 macrophages, M1 macrophages, resting mast cells, monocytes, CD8 T cells, and T follicular helper cells were significantly different in the stages of the different groups (**Figure 5A**). The fraction of activated dendritic cells (p = 0.031), resting dendritic cells (p = 0.00077), M0 macrophages (p = 0.049), M1 macrophages (p = 0.0079), plasma cells (p = 0.019), CD8 T cells (p = 0.0069), and regulatory T cells (Tregs) (p = 0.023) were significantly different in the situation of lymph node metastasis in thyroid carcinoma (**Figure 5B**). The fraction of memory B cells, naive B cells, activated dendritic cells, resting dendritic cells, M1 macrophages, monocytes, plasma cells, activated memory CD4 T cells, and regulatory T cells (Tregs) were significantly different in the four tumor stages of thyroid carcinoma of the different groups (**Figure 5C**). The fraction of memory B cells (p = 0.014), M1 macrophages (p = 0.032), plasma cells (p = 0.046), and CD8 T cells (p = 0.046) were found to be significantly associated with OS (**Figure 5D**).

**Figure 6:**
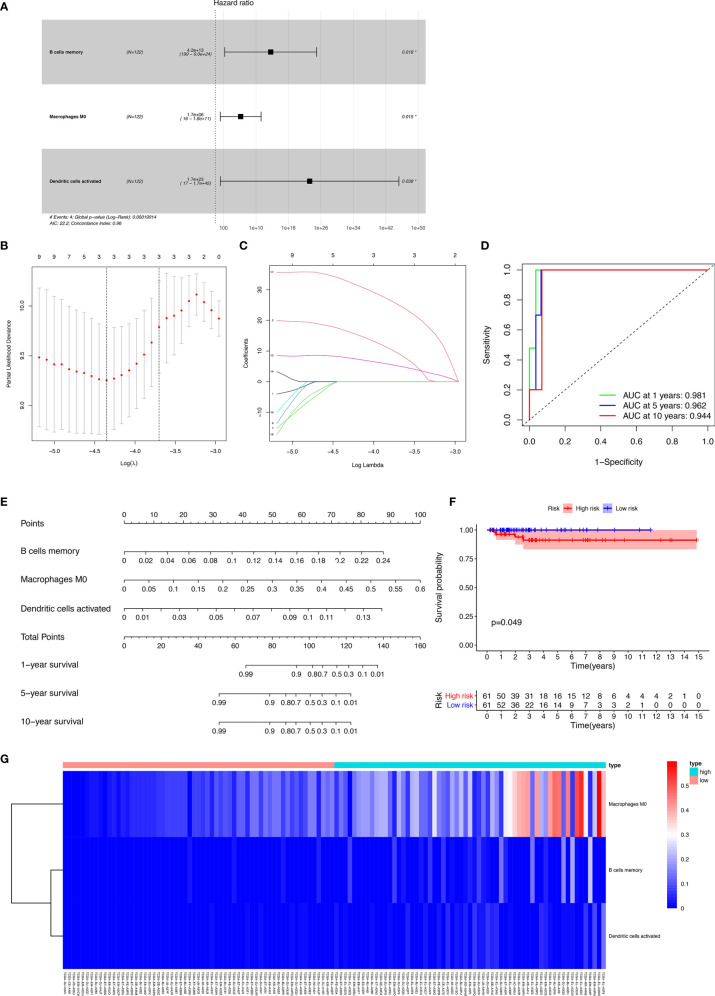
The results of the Lasso regression revealed three immune cells that were key members and were integrated into a new multivariable model **(A–C)**; the ROC demonstrated the nomogram’s good accuracy and concordance **(D)**; constructed nomogram based on immune cells **(E)**; the result of Kaplan-Meier analysis immune cells **(F)**; the heatmap of the three immune cells in the Cox regression model **(G)**.

The results of the Lasso regression revealed that three potential prognosis-related biomarkers were regarded as key members among the 22 immune cell subtypes and were integrated into a new multivariable model (**Figures 6A–C** and **Table 2**). The ROC demonstrated the nomogram’s good accuracy (AUC of 1-year survival: 0.981; 5-year survival: 0.962; AUC of 10-year survival: 0.944) and concordance (**Figure 6D**). The nomogram was constructed based on this model (**Figure 6E**). All the cases were identified as a high or low risk group according to the nomogram model. The proportion of immune cells and the survival of each group are depicted in **Figure 6G**. The result of the Kaplan-Meier analysis showed a significant difference between high and low risk groups (**Figure 6F**). The heat map of the three immune cells in the Cox regression model also showed a significant difference between the high and low risk groups (**Figure 6G**). The immune cells risk score for prognosis was calculated as follows: Immune Cells Risk Score = (31.3769 × memory B cells) + (14.3447 × M0 macrophages) + (53.5019 × activated dendritic cells).

**Table 2:** Cox proportional hazards regression model including the key members of the immune cells for overall survival in patients with thyroid carcinoma.

Immune cells	Hazard Ratio	95%CI	p value
B cells memory	4.2 * 10^13^	(199 - 9 * 10^24^)	0.018*
Macrophages M0	1.7 * 10^6^	(16 - 1.8 * 10^11^)	0.015*
Dendritic cells activated	1.7 * 10^23^	(17 - 1.7 * 10^45^)	0.038*

CI, Confidence Interval. *p < 0.05.

### Co-Expression Analysis

**Figure 7:**
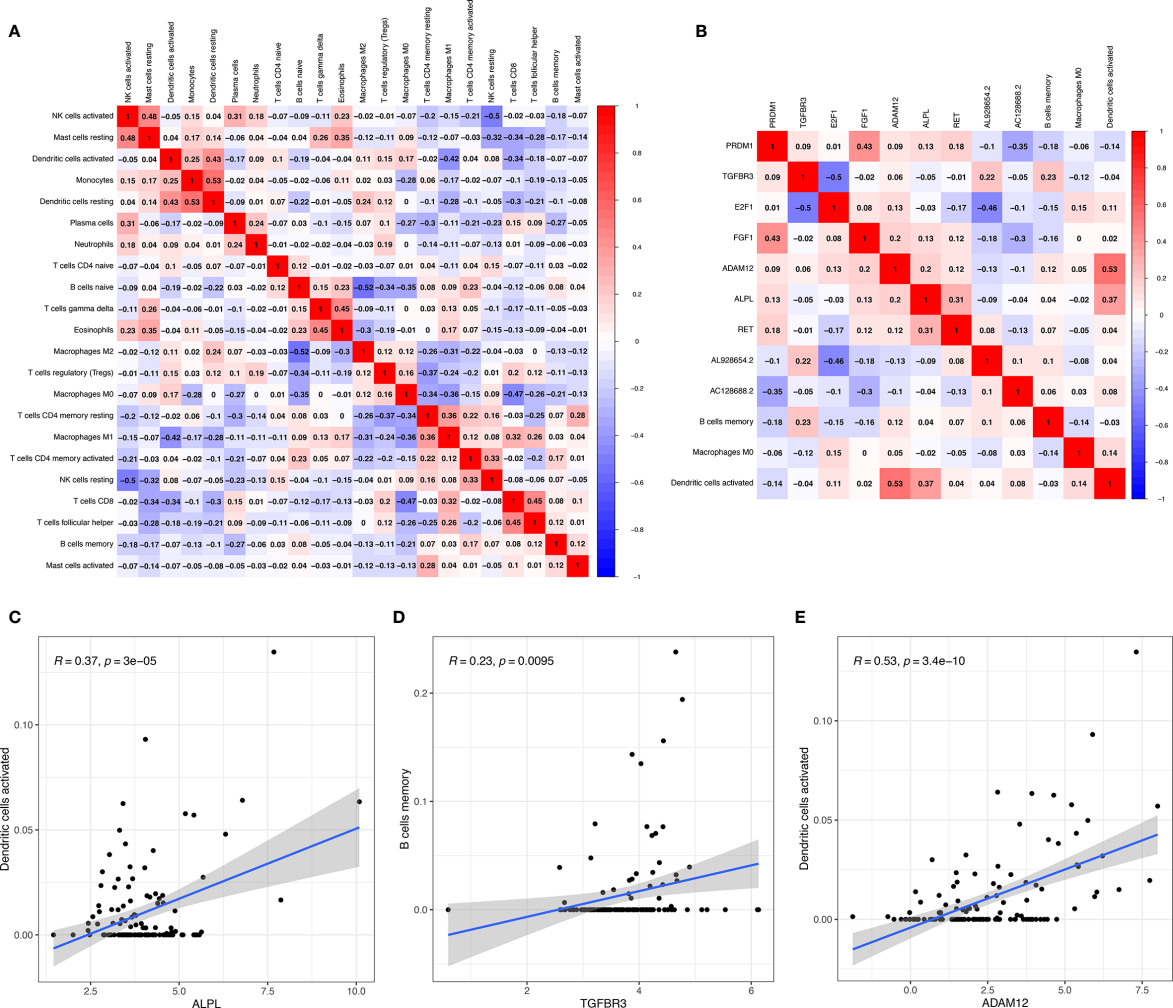
Significant co-expression patterns between proportions of immune cells **(A)**; significant co-expression patterns between ceRNA and immune cells **(B)**; the fraction of activated dendritic cells was positively associated with *ALPL* expression **(C)**; the memory B cells were positively associated with TGFBR3 expression **(D)**; the dendritic cell activated were positively associated with *ADAM12* **(E)**.

Significant co-expression patterns between proportions of immune cells (**Figure 7A**) and ceRNA-immune cells (**Figure 7B**) were analyzed *via* Pearson’s correlation analysis. The fraction of dendritic cells activated was positively associated with *ALPL* expression (R = 0.37, p < 0.001) (**Figure 7C**). The memory B cells were positively associated with *TGFBR3* expression (R = 0.23, p = 0.0095) (**Figure 7D**). The dendritic cell activated were positively associated with *ADAM12* (R = 0.53, p < 0.001) (**Figure 7E**). As for *TGFBR3*, because the R-value in the co-expression results was small, we considered the result not to be meaningful.

### ALPL and CD11c Were Associated With TC

**Figure 8:**
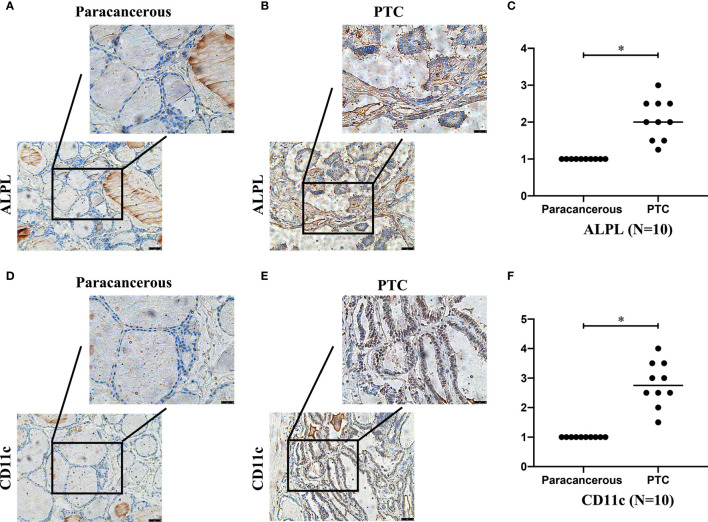
The expressions of ALPL **(A–C)** and CD11c **(D–F)** proteins in papillary thyroid carcinoma **(B, E)** and paracancerous tissues **(A, D)**.

We detected the expression of ALPL and CD11c in papillary thyroid carcinoma (PTC) and paracancerous tissue specimens (N=10). The results suggested that the mean H-score of ALPL in PTC was 2.075, which was significantly higher than in the paracancerous tissue specimens (p < 0.05; **Figures 8A–C**). The mean H-score of CD11c in PTC was 2.800, which was significantly higher than in the paracancerous tissue specimens (p < 0.05; **Figures 8D–F**). The follicular (**Supplementary Figures 3A–D**) and medullary (**Supplementary Figures 3E–H**) thyroid carcinoma have the similar results.

### Multi-Dimensional Validation

A dimensional validation was performed to explore the expressions of ALPL and CD11c (dendritic cell marker) in thyroid carcinoma, normal thyroid tissue, and cell lines (**Table 3**). First, in the Oncomine database, *ALPL* showed no differences across six analyses and CD11c (Gene symbol: *ITGAX*) showed no differences across 11 analyses in thyroid carcinoma compared to normal tissue (**Supplementary Figures 4A–D**). In the UALCAN database, *ALPL* had higher expression in thyroid carcinoma tissue than normal thyroid tissue (p < 0.001). *ITGAX* had no significant difference between thyroid carcinoma tissue and normal thyroid tissue (**Supplementary Figures 5A, B**). However, in the GTEx Portal database, *ALPL* was highly expressed, while *ITGAX* expression in normal thyroid tissue was low (**Supplementary Figures 5C, D**). At the cellular level, in the CCLE database, *ALPL* and *ITGAX* were expressed in various thyroid carcinoma cell lines (**Supplementary Figures 6A–C**). At the protein level, in the STRING database, *ALPL* and *ITGAX* had a significant protein-protein interaction network (**Supplementary Figures 6D, E**). In the Human Protein Atlas database, ALPL protein was not detected in thyroid carcinoma tissue (**Supplementary Figures 6F, G**) but had medium expression in normal thyroid tissue (**Supplementary Figures 6H, I**). CD11c protein expression was high in thyroid carcinoma tissue (**Supplementary Figures 6J, K**), while it was almost not detected in normal thyroid tissue (**Supplementary Figures 6L, M**).

**Table 3:** Summary of multidimensional external validation results based on multiple databases.

Database	ALPL/*ALPL*		CD11c/*ITGAX*		Results
	Cancer	Normal	Cancer	Normal	
Oncomine	—	—	—	—	*ALPL* showed no differences across six analyses and CD11c (Gene symbol: *ITGAX*) showed no differences across eleven analyses in thyroid carcinoma compared to normal tissue (**Supplementary Figures 4A–D**).
UALCAN	↑	↓	—	—	*ALPL* is the higher expression in thyroid carcinoma tissue compared with normal thyroid tissue (p < 0.001). *ITGAX* has no significant difference between thyroid carcinoma tissue and normal thyroid tissue (**Supplementary Figures 5A, B**).
GTEx Portal	—	↑	—	↓	*ALPL* were highly expressed, while *ITGAX* were lowly expressed in normal thyroid tissue (**Supplementary Figures 5C, D**).
CCLE	↑	NA	↑	NA	At the cellular level, *ALPL* and *ITGAX* were expressed in various thyroid cancer cell lines (**Supplementary Figures 6A-C**).
STRING	—	—	—	—	*ALPL* and *ITGAX* had a significant Protein-Protein interaction network (**Supplementary Figures 6D, E**).
The Human Protein Atlas	ND	Medium	High	ND	Protein ALPL is not detected in thyroid carcinoma tissue (**Supplementary Figures 6F, G**), but the medium expression in normal thyroid tissue (**Supplementary Figures 6H, I**). Protein CD11c is high expression in thyroid carcinoma tissue (**Supplementary Figures 6J, K**), while almost not detected in normal thyroid tissue (**Supplementary Figures 6L, M**).

## Discussion

Thyroid carcinoma is the most common endocrine tumor and is characterized by various abnormal molecular events that drive tumorigenesis (28). In recent years, several studies have elucidated the role of non-coding RNA in tumorigenesis, disease development, and metastasis, such as in PTC (29–31). The ceRNAs are transcripts that can regulate each other at the post-transcription level by competing for shared miRNAs. The ceRNA networks link the function of protein-coding mRNAs with non-coding RNAs, such as miRNA, lncRNA, pseudogenic RNA, and circular RNA (32). More and more evidence has shown that ceRNA plays a vital role in a variety of biological pathways (33, 34). At present, the relationship between lncRNA and miRNA has become a key regulator of tumorigenesis and may be regarded as a target for the development of anticancer drugs (35, 36).

The ceRNA network can link non-coding RNA (miRNA, lncRNA) with mRNA, which has the function of protein-coding (14). Competitive binding between lncRNA, mRNA, and miRNA can influence protein expression and biological functions, especially in cancer (37). In this study, we used bioinformatics analysis to identify the ceRNA network that could regulate the prognosis of thyroid carcinoma, which contained 211 protein-encoding mRNAs, 78 lncRNAs, and 28 miRNAs. In this ceRNA network, some genes were significantly related to the OS rate of thyroid cancer. A genetic risk prediction model was constructed based on these risk genes. This model can predict the prognosis of thyroid carcinoma effectively. Furthermore, hypergeometric testing and correlation analysis results of the ceRNA network revealed that hsa-miR-204-5p (miRNA) and *ALPL* (protein-coding RNA) were significantly correlated. Besides, we discovered important tumor-infiltrating immune cells and ceRNA in thyroid carcinoma. Based on these findings, two efficient predictive nomograms were constructed, which could help clinicians evaluate prognosis. The high AUC values of both nomograms proved their clinical application. Meanwhile, the correlation analysis also revealed that *ALPL* was significantly associated with activated dendritic cells (R= 0.37, p < 0.001). By comparing the correlation between thyroid carcinoma-related ceRNA and immune cells, we inferred the underlying mechanism of cancer progression. The mechanism of hsa-miR-204-5p regulating *ALPL* and activated dendritic cells might play an important role in thyroid carcinoma. Finally, a multi-dimensional verification of multiple databases and our IHC results confirmed the reliability of our results.

Some studies have shown that has-miR-204-5p can inhibit tumor cell proliferation, migration, invasion, and metastasis (38–43). Gao et al. (44) found that has-miR-204-5p influences the invasion and metastasis of laryngeal squamous cell carcinoma by specifically regulating FOXC1 expression, thereby inhibiting the malignant behavior, including cell proliferation, invasion, and metastasis, and inhibiting tumor growth *in vivo*. Ma et al. (45) found that PBB12 can act as a microRNA sponge, thereby competitively binding to hsa−miR−204−5p. In addition, PBB12 interferes with the Kruppel-like factor 4 (KLF4)/hsa-miR-204-5p/activating transcription factor 2 (ATF2) pathway and affects the proliferation and invasion of osteosarcoma cells. Besides, hsa-miR-204-5p was identified as a key regulator in the progression of breast cancer (46). Tumor microenvironment-related studies have also found that downregulated hsa-miR-204-5p in osteogenic exosomes could activate PI3K/Akt and MAPK signaling pathways (47). Fan et al. (48) speculated that circKMT2E might act as a sponge molecule of miR-204-5p and play a role in the pathogenesis of diabetic cataracts. It was also found that hsa-miR-204-5p is at a key position in the ceRNA network in several diseases (46, 49–51).

*ALPL* is one of the non-specific tissue genes and also encodes a member of the alkaline phosphatase family of proteins. *ALPL* might restrict the function of the WNT5A–FZD2–STAT3 axis, a non-canonical WNT pathway promoting epithelial-mesenchymal transition progression, which results in attenuated migration and invasion in high grade serous ovarian cancer cells and improves survival in patients with serous ovarian cancer (52). Low expression of *ALPL* has also been associated with the pathogenesis of glioblastoma multiform (53). In prostate cancer, *ALPL* was identified from the protein-protein interaction network, and the sub-networks revealed by this gene are involved in significant pathways (54). The genetic variants of *ALPL* are significantly associated with cutaneous melanoma-specific survival in the folate metabolic pathway genes (55). The role of *ALPL* in thyroid cancer was reported in 2020 (56). Our results suggested that *ALPL* is an important gene that could influence the prognosis of thyroid carcinoma. In addition, *ALPL* was identified as a novel marker of neutrophil activation (57). Therefore, *ALPL* may be associated with immune infiltration.

Dendritic cells (DCs), also known as professional antigen-presenting cells (APC), can promote immunity or tolerance by sampling and presenting antigens to T cells and by providing immunomodulatory signals through cell-cell contact and cytokines (58, 59). DCs constitute a rare immune cell population within tumors and lymphoid organs and interact with other immune cells, such as natural killer cells (NK cells) and B cells. These cells are central for the initiation of antigen-specific immunity and tolerance (60) and activate anti-tumor responses (61). Therefore, manipulation of DCs holds great potential for inducing efficient anti-tumor immunity (60).

The diversity of DC populations, divided by localization and activity, makes its function specific. The former includes Langerhans cells, monocyte-derived DCs (CD14+ DCs), myeloid DCs, and plasmacytoid DCs (pDCs) (62). The latter includes activated DCs and resting DCs (63). DC functions are determined by their integration of environmental signals, which are sensed *via* surface-expressed and intracellular receptors for cytokines, pathogen-associated molecular patterns (PAMPs), and damage-associated molecular patterns (DAMPs) (64). Recent data highlight the specific roles of DC subsets in anti-tumor immunity, with key implications for therapy (65, 66).

In addition, both miR-204-5p and *ALPL* were reported to have effects not only in the intracellular environment but also in the extracellular environment following their secretion. This provides them the opportunity to regulate activated dendritic cells (53, 67, 68).

It should be acknowledged that our research has some unavoidable limitations. First, TCGA is a regularly updated public database, but the sample size and data volume were limited, and the clinical-pathological information was not comprehensive. This may lead to some potential errors or biases. More data should be incorporated to improve the model in the future. Second, we have not considered the heterogeneity of the immune microenvironment related to the location of immune infiltration. The heterogeneity of histological subtypes may affect the accuracy and generalization of the prediction model. Third, all of the data needed to build the model in this study were from patients in Western countries. Therefore, caution should be exercised when applying the conclusions obtained to patients in Asian countries. To minimize the deviation, multiple databases were used to detect the gene and protein expression levels of key biomarkers at the tissue and cell level. However, the results in these databases were not the same. In this regard, this research was only multi-dimensional related research, not biological mechanism research. All the pathological types of thyroid cancer (follicular, medullary, and undifferentiated et al.) should be verified by IHC. Due to the low prevalence of above pathology types, the sample size was limited. The expression of ALPL and CD11c should be verified with a larger sample size, especially follicular, medullary, and undifferentiated thyroid carcinoma. Finally, due to the longer OS of thyroid cancer, we could not use IHC to verify the effects of ALPL and CD11c on the prognosis of thyroid cancer.

## Conclusion

In summary, the deterioration of ceRNA networks may cause to cancer progression and other diseases. Tumor progression and the efficacy of immunotherapy are strongly influenced by the composition and abundance of immune cells in the tumor microenvironment. Given that immune cells play a key role in supporting the initiation and progression of TC, the identification of specific immune targets could improve the efficacy of TC therapy. We screened for thyroid carcinoma-specific differentially expressed lncRNAs, miRNAs, and mRNAs. Based on the ceRNA network and tumor-infiltrating immune cell analysis, two nomograms were established to predict the prognosis of thyroid carcinoma patients. The proposed prediction model may provide a more convincing theoretical basis or provide therapeutic targets for improving the personalized treatment of patients with thyroid cancer. As far as we know, it’s the first time that using tumor-infiltrating immune cells and a ceRNA network model to construct a prognostic analysis model of thyroid carcinoma and was validated by IHC. Our study found the potential mechanism, that hsa-miR-204-5p regulates ALPL so that dendritic cells activated, which may play a critical role in thyroid carcinoma prognosis. These findings may provide potential prognostic biomarkers and therapeutic targets for thyroid carcinoma. In the future, we’ll aim to investigate the exact molecular mechanisms of the relationship and intercellular communication between ceRNA and activated dendritic cells in thyroid carcinoma.

## Data Availability Statement

The original contributions presented in the study are included in the article/**Supplementary Material**. Further inquiries can be directed to the corresponding authors.

## Ethics Statement

The studies involving human participants were reviewed and approved by Institutional Review Board (or Ethics Committee) of the First Hospital of China Medical University. The patients/participants provided their written informed consent to participate in this study.

## Author Contributions

FZ is the first author of this study. WT and DT are the corresponding author supervising this work. FZ managed the case and drafted the manuscript. FZ and XY downloaded the data from the website and performed analysis on all data. FZ and ZL performed the IHC. ZL, XW, and TG assisted in literature review and taking pictures. WT and DT reviewed the manuscript. All authors contributed to the article and approved the submitted version.

## Funding

This research was funded by The Research Fund for Public Welfare, National Health and Family Planning Commission of China, Grant Number 201402005). The funders had no role in the design of the study; in the collection, analyses, or interpretation of data, in the writing of the manuscript, or in the decision to publish the results.

## Conflict of Interest

The authors declare that the research was conducted in the absence of any commercial or financial relationships that could be construed as a potential conflict of interest.

## Supplementary Material

Supplementary Figure 1 The functional role of mRNAs was assessed using the clusterProfiler package. Gene ontology **(A, C)**, Kyoto encyclopedia of genes and genomes **(B, D)**.

Supplementary Figure 2 Forty-two genes were significant in the Kaplan-Meier analysis.

Supplementary Figure 3 The expression of ALPL and CD11c in the follicular **(A-D)** and medullary **(E-H)** thyroid carcinoma. The ALPL and CD11c was higher in thyroid carcinoma tissues **(A, C, E, G)** compared to the paracancerous tissue specimens **(B, D, F, H)**.

Supplementary Figure 4 *ALPL* and CD11c (*ITGAX*) expression in the Oncomine database.

Supplementary Figure 5 ALPL and CD11c(*ITGAX*) expression. **(A, B)** UALCAN database; **(C, D)** GTEx Portal database.

Supplementary Figure 6 *ALPL* and *ITGAX* expression. **(A–C)** CCLE database; **(D, E)** STRING database; **(F–M)** Human Protein Atlas database; **(F, G)** ALPL was not detected in thyroid carcinoma tissue; **(H, I)** ALPL had medium expression in normal thyroid tissue; **(J, K)** CD11c had high expression in thyroid carcinoma tissue; **(L, M)** CD11c was not detected in normal thyroid tissue.

## Abbreviations

TCGA, The Cancer Genome Atlas; ceRNA, competitive endogenous RNA; miRNA, microRNA; lncRNA, long noncoding RNA; OS, overall survival; AUC, the area under the curve; IHC, immunohistochemistry; CIBERSORT, cell type identification by estimating relative subsets of RNA transcripts; FDR, false discovery rate; GO, Gene Ontology; KEGG, Kyoto Encyclopedia of Genes and Genomes; BP, biological process; MF, molecular function; CC, cellular component; ROC, receiver operating characteristic curves; GTEx, Genotype-Tissue Expression; CCLE, Cancer Cell Line Encyclopedia; DAB, 3, 3- diaminobenzidine tetrahydrochloride; H-score, histochemistry score; Tregs, T cells regulatory; KLF4, Kruppel-like factor 4; ATF2, activating transcription factor 2; DCs, Dendritic cells; APC, antigen-presenting cells; pDCs, plasmacytoid DCs; PAMPs, pathogen-associated molecular patterns; DAMPs, damage-associated molecular patterns; TC, thyroid carcinoma.
