# Supplementary figures and images for: Using Tumor-Infiltrating Immune Cells and a ceRNA Network Model to Construct a Prognostic Analysis Model of Thyroid Carcinoma
Source: Front Oncol. 2021 Jun 1;11:658165. doi: 10.3389/fonc.2021.658165 (PMC8204697; doi:10.3389/fonc.2021.658165)

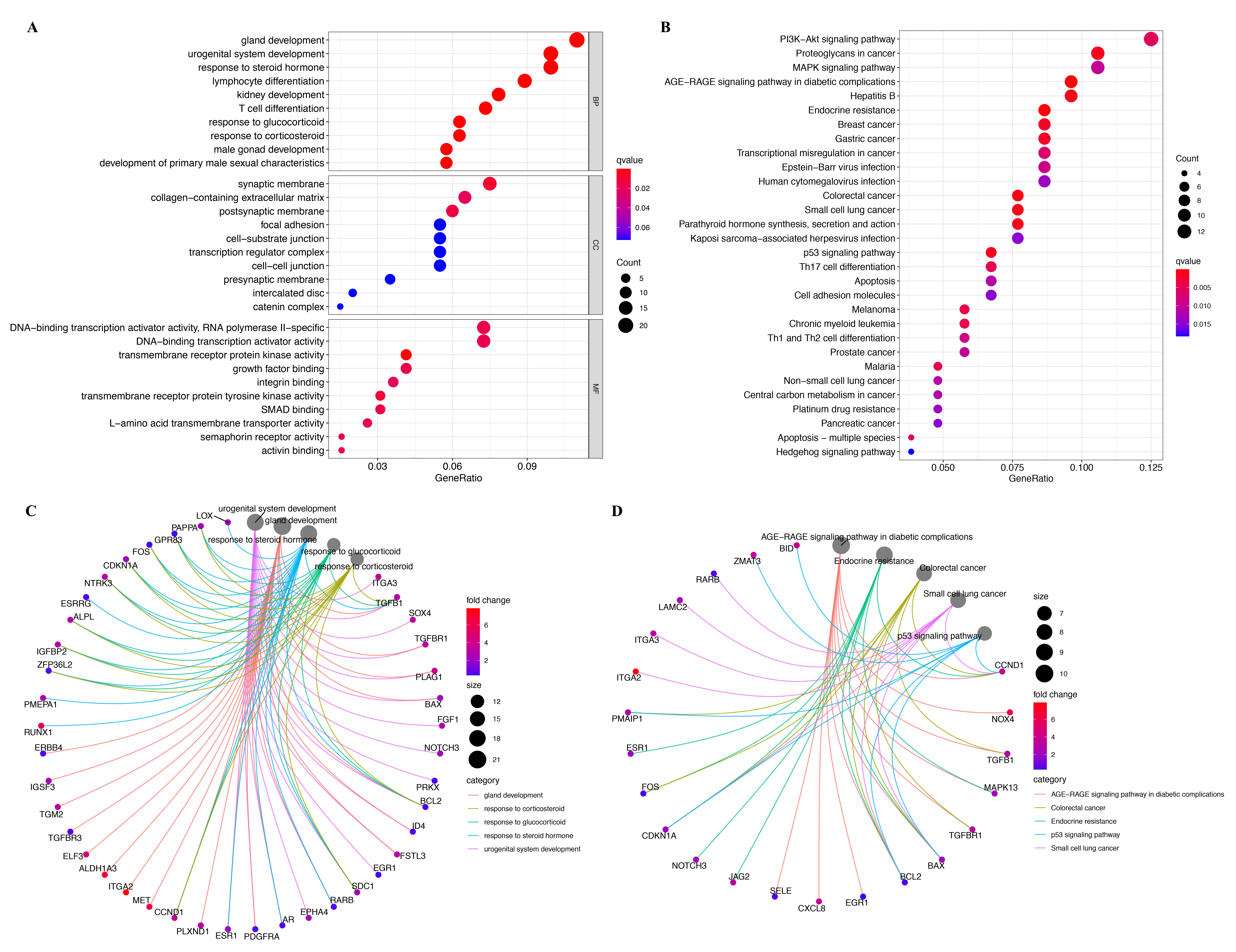

Supplement: Supplementary Figure 1 — The functional role of mRNAs was assessed using the clusterProfiler package. Gene ontology (A, C), Kyoto encyclopedia of genes and genomes (B, D). [file Image_1.tif]

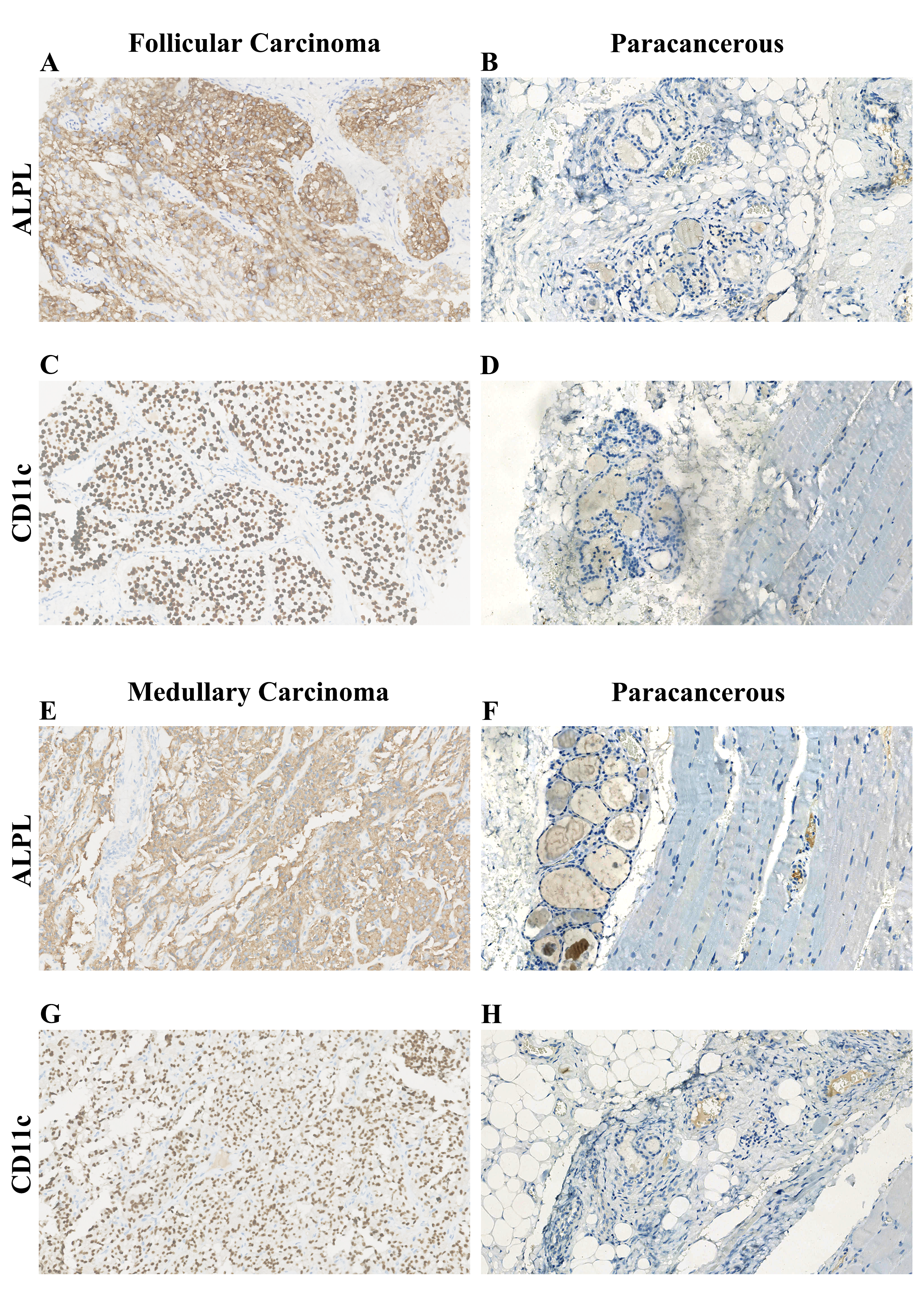

Supplement: Supplementary Figure 3 — The expression of ALPL and CD11c in the follicular (A-D) and medullary (E-H) thyroid carcinoma. The ALPL and CD11c was higher in thyroid carcinoma tissues (A, C, E, G) compared to the paracancerous tissue specimens (B, D, F, H). [file Image_3.tif]

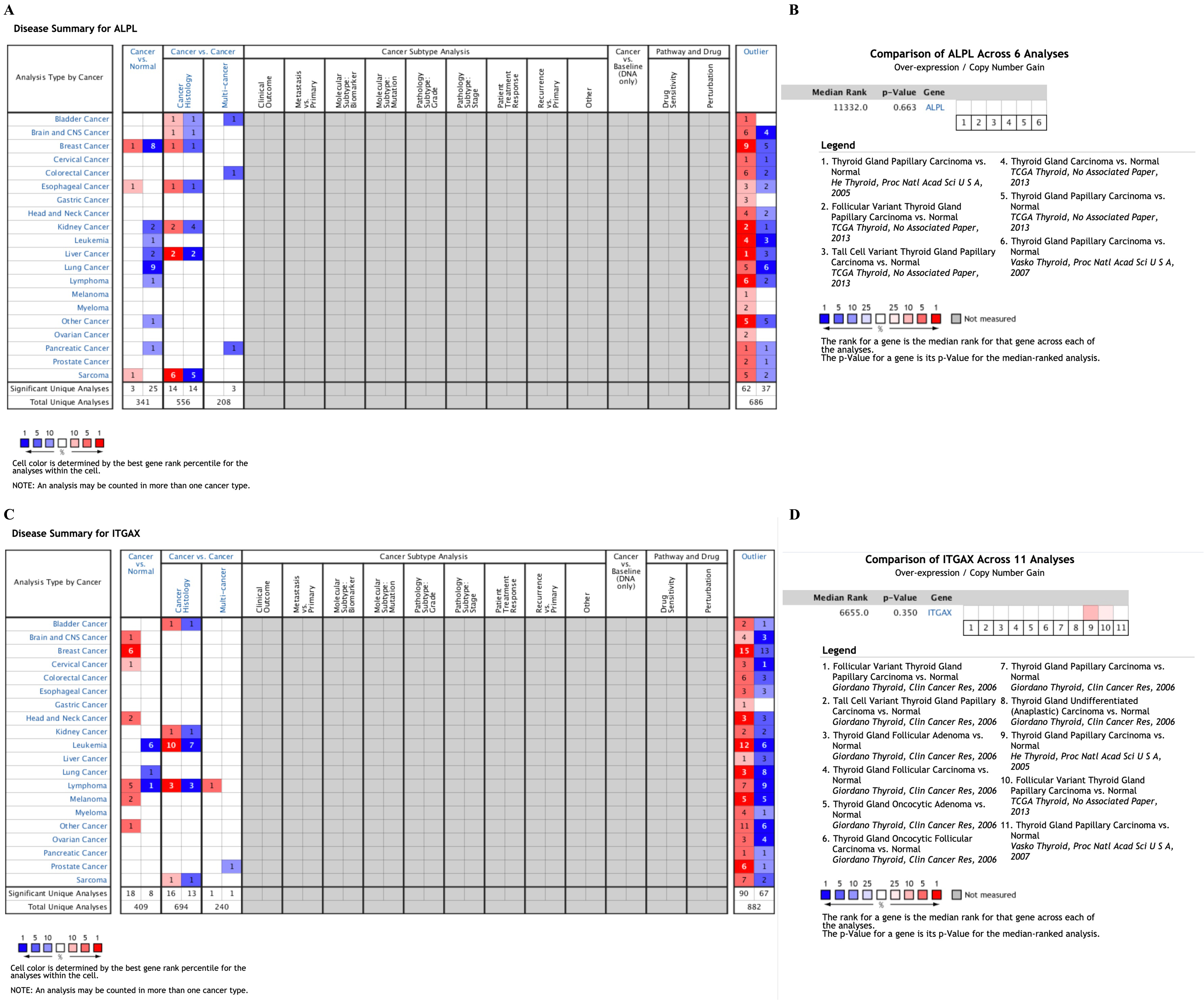

Supplement: Supplementary Figure 4 — ALPL and CD11c (ITGAX) expression in the Oncomine database. [file Image_4.tif]
