# Supplementary material for: Using Tumor-Infiltrating Immune Cells and a ceRNA Network Model to Construct a Prognostic Analysis Model of Thyroid Carcinoma
Source: Front Oncol. 2021 Jun 1;11:658165. doi: 10.3389/fonc.2021.658165 (PMC8204697; doi:10.3389/fonc.2021.658165)
Supplement: Supplementary file 7 [file Table_1.docx]

**Supplementary Table 1. Baseline information of 502 patients diagnosed with thyroid cancer.**

| **Variables** | | **Total Patients (n=502)** |
| --- | --- | --- |
| **Age, years** | |  |
|  | Mean ± SD | 47.27±15.77 |
|  | Median (Range) | 46 (15-89) |
| **Gender** | |  |
|  | Female | 366 (72.91%) |
|  | Male | 136 (27.09%) |
| **Race** | |  |
|  | Asian | 51 (10.16%) |
|  | Black or African American | 27 (5.38%) |
|  | White | 332 (66.14%) |
|  | American Indian or Alaska native | 1 (0.19%) |
|  | Not reported | 91 (18.13%) |
| **T** | |  |
|  | T1 | 143 (28.49%) |
|  | T2 | 164 (32.67%) |
|  | T3 | 168 (33.47%) |
|  | T4 | 23 (4.58%) |
|  | TX | 4 (0.79%) |
| **N** | |  |
|  | N0 | 228 (45.42%) |
|  | N1 | 222 (44.22%) |
|  | NX | 52 (10.36%) |
| **M** | |  |
|  | M0 | 282 (56.18%) |
|  | M1 | 9 (1.79%) |
|  | MX | 211 (42.03%) |
| **Pathologic Stage** | |  |
|  | Stage I | 284 (56.%) |
|  | Stage II | 52 (10.36%) |
|  | Stage III | 111 (22.11%) |
|  | Stage IV | 55 (10.96%) |
