# Supplementary material for: Using Tumor-Infiltrating Immune Cells and a ceRNA Network Model to Construct a Prognostic Analysis Model of Thyroid Carcinoma
Source: Front Oncol. 2021 Jun 1;11:658165. doi: 10.3389/fonc.2021.658165 (PMC8204697; doi:10.3389/fonc.2021.658165)
Supplement: Supplementary file 8 [file Table_2.docx]

**Supplementary Table 2. The list of top 10 downregulated and top 10 upregulated genes in differential gene analysis.**

| **Genes** | **Type** | **logFC** | ***p* Value** | **FDR** |
| --- | --- | --- | --- | --- |
| ARHGAP36 | protein_coding | 7.4975483 | 5.80E-82 | 8.55E-80 |
| GABRB2 | protein_coding | 7.13359451 | 1.23E-202 | 1.87E-198 |
| AL157714.2 | long_non_coding | 7.12833635 | 2.89E-124 | 2.74E-121 |
| SLC22A31 | protein_coding | 7.10605791 | 5.17E-157 | 1.96E-153 |
| TMPRSS6 | protein_coding | 7.09905207 | 2.23E-114 | 1.47E-111 |
| KLK11 | protein_coding | 7.01507314 | 5.78E-72 | 5.92E-70 |
| ZCCHC12 | protein_coding | 6.77349191 | 6.78E-130 | 1.03E-126 |
| PRSS2 | protein_coding | 6.75600401 | 2.37E-78 | 3.24E-76 |
| SYT12 | protein_coding | 6.61223023 | 1.10E-107 | 5.37E-105 |
| IVL | protein_coding | 6.49582025 | 2.57E-62 | 1.83E-60 |
| DPT | protein_coding | -3.28082 | 3.43E-27 | 4.38E-26 |
| CCL21 | protein_coding | -3.2447595 | 6.63E-18 | 4.44E-17 |
| MMRN1 | protein_coding | -3.1420591 | 1.58E-56 | 8.79E-55 |
| TFF3 | protein_coding | -3.1189012 | 3.89E-20 | 3.04E-19 |
| LYVE1 | protein_coding | -2.9936125 | 3.79E-63 | 2.80E-61 |
| CHRDL1 | protein_coding | -2.9485063 | 8.95E-24 | 9.05E-23 |
| RYR2 | protein_coding | -2.9153006 | 1.74E-34 | 3.48E-33 |
| IPCEF1 | protein_coding | -2.9020402 | 1.22E-35 | 2.64E-34 |
| PLA2R1 | protein_coding | -2.7887497 | 2.88E-53 | 1.42E-51 |
| TFCP2L1 | protein_coding | -2.7600943 | 1.42E-31 | 2.44E-30 |

Abbreviations: FC: Fold change; FDR: False Discovery Rate.
