# Supplementary material for: Using Tumor-Infiltrating Immune Cells and a ceRNA Network Model to Construct a Prognostic Analysis Model of Thyroid Carcinoma
Source: Front Oncol. 2021 Jun 1;11:658165. doi: 10.3389/fonc.2021.658165 (PMC8204697; doi:10.3389/fonc.2021.658165)
Supplement: Supplementary file 9 [file Table_3.docx]

| **Supplementary Table 3. Hypergeometric testing and correlation analysis results of ceRNAs network.** | | | | |
| --- | --- | --- | --- | --- |
| **lncRNAs** | **miRNAs** | **mRNA** | **hyperPValue** | **corPValue** |
| AL157714.2 | hsa-miR-150-5p | TMEM92 | 0.032777083 | 3.34E-104 |
| AL157714.2 | hsa-miR-204-5p | BID | 0.003459286 | 3.48E-124 |
| AL157714.2 | hsa-miR-214-3p | JAG2 | 0.004968646 | 3.29E-19 |
| AL157714.2 | hsa-miR-204-5p | SAMD1 | 0.040113551 | 7.98E-47 |
| AL157714.2 | hsa-miR-150-5p, hsa-miR-205-5p | XPR1 | 0.001054142 | 4.41E-49 |
| AL157714.2 | hsa-miR-204-5p | CDH4 | 0.004607202 | 6.15E-48 |
| AL157714.2 | hsa-miR-204-5p | SH2D4A | 0.016477616 | 3.05E-73 |
| AL157714.2 | hsa-miR-363-3p | PLXNA3 | 0.013431656 | 7.68E-69 |
| AL157714.2 | hsa-miR-204-5p, hsa-miR-363-3p | SOX4 | 0.002638846 | 2.26E-82 |
| AL157714.2 | hsa-miR-363-3p | TOR4A | 0.002036688 | 3.22E-53 |
| AL157714.2 | hsa-miR-214-3p | LZTS1 | 0.001057433 | 8.41E-23 |
| AL157714.2 | hsa-miR-363-3p | PMEPA1 | 0.011511979 | 2.69E-40 |
| AL157714.2 | hsa-miR-214-3p | TRIM29 | 0.000350014 | 7.75E-48 |
| AL157714.2 | hsa-miR-150-5p | NOTCH3 | 0.002459651 | 3.97E-17 |
| AL157714.2 | hsa-miR-181a-5p | S100A1 | 0.006077435 | 9.45E-33 |
| AL157714.2 | hsa-miR-214-3p | RAB15 | 0.030398331 | 9.91E-22 |
| AL157714.2 | hsa-miR-150-5p, hsa-miR-214-3p | TNFSF15 | 0.025184467 | 4.74E-31 |
| AL157714.2 | hsa-miR-204-5p | PI4K2A | 0.004607202 | 1.39E-29 |
| AL157714.2 | hsa-miR-199b-5p | LAMC2 | 0.026039876 | 4.79E-32 |
| AL157714.2 | hsa-miR-181a-5p, hsa-miR-181b-5p, hsa-miR-181d-5p | DCBLD2 | 0.016822617 | 3.98E-31 |
| AL157714.2 | hsa-miR-204-5p | ALPL | 0.032704886 | 0.019119228 |
| AL157714.2 | hsa-miR-214-3p | ASF1B | 0.032777083 | 5.87E-23 |
| AL157714.2 | hsa-miR-204-5p | PLAUR | 0.014556915 | 1.39E-54 |
| AL157714.2 | hsa-miR-204-5p | CREB5 | 0.007130559 | 1.04E-81 |
| AL157714.2 | hsa-miR-204-5p | STEAP4 | 0.022403097 | 1.25E-17 |
| AL157714.2 | hsa-miR-363-3p | FAM129A | 0.002998222 | 2.08E-16 |
| AL157714.2 | hsa-miR-181a-5p, hsa-miR-181b-5p, hsa-miR-181d-5p | ARSJ | 0.001055277 | 1.78E-41 |
| AL157714.2 | hsa-miR-363-3p | GOLGA8A | 0.002088714 | 1.49E-06 |
| AL157714.2 | hsa-miR-181a-5p, hsa-miR-34a-5p | PDGFRA | 0.047117587 | 0.000159277 |
| AL157714.2 | hsa-miR-34a-5p | ZAP70 | 0.006077435 | 0.003537464 |
| LINC02471 | hsa-miR-34a-5p | HMGA2 | 0.004995316 | 2.05E-112 |
| LINC02471 | hsa-miR-34a-5p | GALNT7 | 0.003432735 | 2.04E-123 |
| LINC02471 | hsa-miR-34a-5p | MET | 0.000543056 | 7.79E-71 |
| LINC02471 | hsa-miR-34a-5p | CD44 | 0.003412825 | 2.17E-80 |
| LINC02471 | hsa-miR-199b-5p | C1orf226 | 0.018373224 | 5.86E-30 |
| LINC02471 | hsa-miR-375 | PLAG1 | 0.039200604 | 8.66E-68 |
| LINC02471 | hsa-miR-34a-5p, hsa-miR-375 | TGM2 | 0.00166888 | 1.91E-20 |
| LINC02471 | hsa-miR-375 | SDC1 | 0.009064335 | 6.66E-10 |
| AC025419.1 | hsa-miR-150-5p | TMEM92 | 0.043425559 | 5.05E-56 |
| AC025419.1 | hsa-miR-144-3p, hsa-miR-31-5p | MET | 0.022122445 | 3.94E-73 |
| AC025419.1 | hsa-miR-214-3p | JAG2 | 0.001138924 | 1.78E-20 |
| AC025419.1 | hsa-miR-138-5p | LCN2 | 0.038091574 | 1.28E-47 |
| AC025419.1 | hsa-miR-150-5p, hsa-miR-205-5p, hsa-miR-375 | XPR1 | 0.000258122 | 2.48E-55 |
| AC025419.1 | hsa-miR-20b-5p | LIMK1 | 0.00062659 | 2.31E-30 |
| AC025419.1 | hsa-miR-9-5p, hsa-miR-181a-5p | RUNX1 | 0.040104619 | 4.40E-56 |
| AC025419.1 | hsa-miR-146b-5p, hsa-miR-375 | IL1RAP | 0.007080122 | 3.77E-58 |
| AC025419.1 | hsa-miR-9-5p | RAB34 | 0.001483679 | 4.27E-65 |
| AC025419.1 | hsa-miR-363-3p | TOR4A | 0.00135537 | 2.40E-56 |
| AC025419.1 | hsa-miR-138-5p | CDH2 | 0.001730084 | 5.90E-06 |
| AC025419.1 | hsa-miR-214-3p | LZTS1 | 0.001436865 | 3.78E-15 |
| AC025419.1 | hsa-miR-181a-5p, hsa-miR-214-3p | BAX | 0.000160697 | 6.37E-35 |
| AC025419.1 | hsa-miR-363-3p | PMEPA1 | 0.006307091 | 2.28E-29 |
| AC025419.1 | hsa-miR-150-5p | NOTCH3 | 0.003810686 | 1.69E-12 |
| AC025419.1 | hsa-miR-138-5p, hsa-miR-181a-5p | S100A1 | 0.000208718 | 1.14E-44 |
| AC025419.1 | hsa-miR-9-5p | PXDN | 0.017717167 | 4.33E-07 |
| AC025419.1 | hsa-miR-150-5p, hsa-miR-214-3p | TNFSF15 | 0.048119968 | 7.88E-28 |
| AC025419.1 | hsa-miR-9-5p, hsa-miR-21-5p | TGFBI | 0.008541223 | 1.02E-20 |
| AC025419.1 | hsa-miR-9-5p, hsa-miR-375 | FSTL3 | 0.014913288 | 3.13E-37 |
| AC025419.1 | hsa-miR-214-3p | ASF1B | 0.000215591 | 5.46E-39 |
| AC025419.1 | hsa-miR-9-5p, hsa-miR-375 | SDC1 | 0.002533282 | 3.36E-09 |
| AC025419.1 | hsa-miR-138-5p, hsa-miR-182-5p | CASTOR2 | 0.008172354 | 1.19E-18 |
| AC025419.1 | hsa-miR-20b-5p, hsa-miR-181a-5p, hsa-miR-181b-5p, hsa-miR-181d-5p | SCD | 0.000326095 | 8.35E-35 |
| AC025419.1 | hsa-miR-9-5p, hsa-miR-144-3p, hsa-miR-205-5p | SLC7A2 | 0.026452628 | 2.62E-14 |
| AC025419.1 | hsa-miR-20b-5p | CTSS | 0.000119356 | 7.80E-40 |
| AC025419.1 | hsa-miR-363-3p | GOLGA8A | 0.004522502 | 1.26E-13 |
| AC025419.1 | hsa-miR-214-3p | PAPPA | 0.000550886 | 3.00E-05 |
| AC025419.1 | hsa-miR-146b-5p, hsa-miR-150-5p | CYTIP | 0.045867633 | 0.002895046 |
| LINC02454 | hsa-miR-9-5p | CDH3 | 0.019502315 | 7.18E-144 |
| LINC02454 | hsa-miR-9-5p | P4HA2 | 0.013041671 | 4.21E-76 |
| LINC02454 | hsa-miR-9-5p | CCND1 | 0.000600494 | 2.37E-75 |
| LINC02454 | hsa-miR-9-5p | NOX4 | 0.032304375 | 1.70E-50 |
| LINC02454 | hsa-miR-9-5p | RUNX1 | 0.000745909 | 1.07E-78 |
| LINC02454 | hsa-miR-9-5p | RAB34 | 0.01998201 | 1.20E-93 |
| LINC02454 | hsa-miR-9-5p | ENDOD1 | 0.01193679 | 6.67E-92 |
| LINC02454 | hsa-miR-9-5p | GALNTL6 | 0.02592314 | 7.06E-94 |
| LINC02454 | hsa-miR-9-5p | PRDM1 | 0.000489478 | 4.26E-58 |
| LINC02454 | hsa-miR-9-5p | PXDN | 0.012842337 | 9.25E-06 |
| LINC02454 | hsa-miR-9-5p | PI4K2A | 0.002140023 | 1.40E-39 |
| LINC02454 | hsa-miR-9-5p | TGFBI | 0.008615666 | 8.09E-25 |
| LINC02454 | hsa-miR-9-5p | FSTL3 | 0.025892658 | 9.92E-59 |
| LINC02454 | hsa-miR-9-5p | SLC7A2 | 0.002784533 | 1.05E-17 |
| LINC02454 | hsa-miR-9-5p | ESR1 | 0.00098109 | 9.32E-09 |
| LINC02555 | hsa-miR-96-5p, hsa-miR-183-5p, hsa-miR-34a-5p | CCND1 | 0.000367179 | 6.93E-48 |
| LINC02555 | hsa-miR-7-5p, hsa-miR-205-5p, hsa-miR-375 | XPR1 | 0.004554791 | 3.23E-32 |
| LINC02555 | hsa-miR-7-5p | TRIM47 | 0.013851481 | 1.66E-31 |
| LINC02555 | hsa-miR-7-5p | RNF183 | 0.04099707 | 4.26E-07 |
| LINC02555 | hsa-miR-7-5p | FNDC4 | 0.027516357 | 2.29E-39 |
| LINC02555 | hsa-miR-181a-5p | TGFBR1 | 0.032251931 | 1.83E-20 |
| LINC02555 | hsa-miR-7-5p | SSX1 | 0.013851481 | 1.36E-09 |
| LINC02555 | hsa-miR-205-5p, hsa-miR-222-3p | RUNX2 | 0.000501366 | 1.46E-29 |
| LINC02555 | hsa-miR-7-5p, hsa-miR-96-5p, hsa-miR-183-5p, hsa-miR-34a-5p | EFHD2 | 0.005090587 | 1.14E-66 |
| LINC02555 | hsa-miR-181a-5p, hsa-miR-181b-5p, hsa-miR-181d-5p | SCD | 0.002550202 | 1.94E-19 |
| LINC02555 | hsa-miR-7-5p, hsa-miR-181a-5p | RGS5 | 0.029075397 | 2.39E-10 |
| LINC02555 | hsa-miR-7-5p | ROR1 | 0.004957777 | 0.02187035 |
| LINC02555 | hsa-miR-181a-5p, hsa-miR-181b-5p | PBX3 | 0.01322251 | 2.12E-43 |
| LINC02555 | hsa-miR-181a-5p, hsa-miR-181b-5p, hsa-miR-181d-5p | ARSJ | 0.000121163 | 0.000564989 |
| AL158206.1 | hsa-miR-9-5p | CDH3 | 0.049952682 | 2.11E-47 |
| AL158206.1 | hsa-miR-9-5p | P4HA2 | 0.033578972 | 1.41E-32 |
| AL158206.1 | hsa-miR-9-5p, hsa-miR-20b-5p | CCND1 | 0.000637416 | 4.07E-85 |
| AL158206.1 | hsa-miR-9-5p | KCNJ2 | 0.0010888 | 4.68E-36 |
| AL158206.1 | hsa-miR-20b-5p | PDE4C | 0.001789899 | 2.32E-60 |
| AL158206.1 | hsa-miR-20b-5p | PSD3 | 0.01384264 | 1.14E-17 |
| AL158206.1 | hsa-miR-9-5p | PXDN | 0.009975158 | 2.86E-13 |
| AL158206.1 | hsa-miR-150-5p, hsa-miR-20b-5p | SLC1A5 | 0.000470597 | 3.19E-19 |
| AL158206.1 | hsa-miR-150-5p, hsa-miR-181a-5p | MMP14 | 0.000679845 | 4.55E-18 |
| AL158206.1 | hsa-miR-20b-5p, hsa-miR-181a-5p | CDKN1A | 0.04537813 | 1.87E-54 |
| AL158206.1 | hsa-miR-9-5p | SLC7A2 | 0.006426521 | 1.06E-07 |
| STK32A-AS1 | hsa-miR-181a-5p, hsa-miR-181b-5p, hsa-miR-181d-5p | HMGA2 | 0.00312017 | 2.79E-85 |
| STK32A-AS1 | hsa-miR-9-5p | CDH3 | 0.035371333 | 3.33E-68 |
| STK32A-AS1 | hsa-miR-9-5p | P4HA2 | 0.023717596 | 1.02E-48 |
| STK32A-AS1 | hsa-miR-214-3p | ENTPD1 | 0.03920078 | 6.56E-64 |
| STK32A-AS1 | hsa-miR-9-5p | CCND1 | 0.007439072 | 1.17E-52 |
| STK32A-AS1 | hsa-miR-9-5p | NOX4 | 0.001346816 | 1.84E-32 |
| STK32A-AS1 | hsa-miR-214-3p | JAG2 | 0.002030539 | 6.58E-24 |
| STK32A-AS1 | hsa-miR-214-3p | AHNAK2 | 0.000775107 | 4.51E-42 |
| STK32A-AS1 | hsa-miR-9-5p | RAB34 | 0.000598783 | 4.47E-50 |
| STK32A-AS1 | hsa-miR-181a-5p | TGFBR1 | 0.021740854 | 4.25E-34 |
| STK32A-AS1 | hsa-miR-9-5p | GALNTL6 | 0.046890396 | 4.10E-53 |
| STK32A-AS1 | hsa-miR-214-3p | KCNK5 | 0.024734324 | 2.66E-48 |
| STK32A-AS1 | hsa-miR-214-3p | TRIM29 | 0.001527634 | 1.32E-10 |
| STK32A-AS1 | hsa-miR-9-5p | PRDM1 | 0.034839288 | 3.34E-45 |
| STK32A-AS1 | hsa-miR-181a-5p | S100A1 | 0.046890396 | 4.08E-28 |
| STK32A-AS1 | hsa-miR-214-3p | TNFSF15 | 0.008769097 | 2.71E-24 |
| STK32A-AS1 | hsa-miR-9-5p | RHOV | 0.007083866 | 2.66E-13 |
| STK32A-AS1 | hsa-miR-181a-5p, hsa-miR-181b-5p, hsa-miR-181d-5p | DCBLD2 | 0.000801403 | 2.06E-20 |
| STK32A-AS1 | hsa-miR-9-5p | SDC1 | 0.014892981 | 0.002783858 |
| STK32A-AS1 | hsa-miR-181a-5p | MMP14 | 0.007083866 | 1.86E-07 |
| STK32A-AS1 | hsa-miR-181a-5p | CDKN1A | 0.035636172 | 8.06E-16 |
| STK32A-AS1 | hsa-miR-181a-5p, hsa-miR-181b-5p | PBX3 | 0.009898121 | 1.42E-10 |
| AC007255.1 | hsa-miR-7-5p | XPR1 | 0.02859146 | 7.59E-49 |
| AC007255.1 | hsa-miR-7-5p | TRIM47 | 0.00115429 | 4.20E-28 |
| AC007255.1 | hsa-miR-7-5p | ST3GAL5 | 0.020641546 | 2.98E-82 |
| AC007255.1 | hsa-miR-7-5p | UNC5CL | 0.009209459 | 2.90E-45 |
| AC007255.1 | hsa-miR-7-5p | RNF183 | 0.003460205 | 6.48E-35 |
| AC007255.1 | hsa-miR-7-5p | FNDC4 | 0.002307692 | 8.89E-38 |
| AC007255.1 | hsa-miR-7-5p | ALDH1A3 | 0.036498236 | 1.07E-17 |
| AC007255.1 | hsa-miR-7-5p | GLDN | 0.018362202 | 2.98E-54 |
| AC007255.1 | hsa-miR-7-5p | BAX | 0.017221204 | 3.06E-17 |
| AC007255.1 | hsa-miR-7-5p | FGF1 | 0.02859146 | 1.37E-29 |
| AC007255.1 | hsa-miR-7-5p | SLPI | 0.029723638 | 6.28E-23 |
| AC007255.1 | hsa-miR-7-5p | SSX1 | 0.00115429 | 6.65E-17 |
| AC007255.1 | hsa-miR-7-5p | TMEM98 | 0.022917358 | 6.11E-45 |
| AC007255.1 | hsa-miR-7-5p | LAMC2 | 0.009209459 | 1.98E-13 |
| AC007255.1 | hsa-miR-7-5p | ARMCX6 | 0.026324463 | 1.06E-68 |
| AC007255.1 | hsa-miR-7-5p | TGM2 | 0.008061382 | 1.17E-14 |
| AC007255.1 | hsa-miR-7-5p | EFHD2 | 0.002961026 | 1.03E-37 |
| AC007255.1 | hsa-miR-7-5p | IGSF3 | 0.02859146 | 8.48E-07 |
| AC007255.1 | hsa-miR-7-5p | DSC2 | 0.008061382 | 4.67E-12 |
| AC007255.1 | hsa-miR-7-5p | ROR1 | 0.009209459 | 1.58E-13 |
| AC007255.1 | hsa-miR-7-5p | PAPPA | 0.021779893 | 4.93E-09 |
| AP002358.1 | hsa-miR-20b-5p | ZMAT3 | 0.000133701 | 5.65E-45 |
| AP002358.1 | hsa-miR-20b-5p | DRAXIN | 0.000156062 | 5.59E-100 |
| AP002358.1 | hsa-miR-20b-5p | PDE4C | 0.005893951 | 1.98E-74 |
| AP002358.1 | hsa-miR-204-5p | BID | 0.007528329 | 1.32E-124 |
| AP002358.1 | hsa-miR-20b-5p | MANEAL | 0.00040973 | 1.31E-19 |
| AP002358.1 | hsa-miR-204-5p | SAMD1 | 0.017138757 | 1.37E-35 |
| AP002358.1 | hsa-miR-7-5p | TRIM47 | 0.020777222 | 2.56E-72 |
| AP002358.1 | hsa-miR-204-5p | CDH4 | 0.020670943 | 3.42E-39 |
| AP002358.1 | hsa-miR-204-5p | SH2D4A | 0.04721742 | 2.39E-59 |
| AP002358.1 | hsa-miR-7-5p | FNDC4 | 0.000423862 | 5.92E-52 |
| AP002358.1 | hsa-miR-20b-5p | HIP1 | 0.000690763 | 2.54E-14 |
| AP002358.1 | hsa-miR-363-3p | PLXNA3 | 0.005255803 | 7.82E-80 |
| AP002358.1 | hsa-miR-20b-5p | QSOX1 | 0.001536897 | 6.31E-99 |
| AP002358.1 | hsa-miR-363-3p | TOR4A | 0.000583892 | 2.35E-37 |
| AP002358.1 | hsa-miR-204-5p | TGFBR1 | 0.003646803 | 1.45E-117 |
| AP002358.1 | hsa-miR-7-5p, hsa-miR-34a-5p | BAX | 0.003233779 | 8.55E-25 |
| AP002358.1 | hsa-miR-204-5p | B4GALNT3 | 0.015329897 | 3.62E-65 |
| AP002358.1 | hsa-miR-7-5p, hsa-miR-363-3p | PMEPA1 | 0.000166585 | 1.16E-51 |
| AP002358.1 | hsa-miR-7-5p | SSX1 | 0.020777222 | 3.12E-35 |
| AP002358.1 | hsa-miR-204-5p, hsa-miR-34a-5p | HRK | 0.010961404 | 1.79E-23 |
| AP002358.1 | hsa-miR-204-5p | PI4K2A | 0.001243924 | 5.21E-49 |
| AP002358.1 | hsa-miR-7-5p | LAMC2 | 0.010951884 | 2.24E-58 |
| AP002358.1 | hsa-miR-7-5p, hsa-miR-34a-5p | TGM2 | 0.00027994 | 1.46E-63 |
| AP002358.1 | hsa-miR-20b-5p, hsa-miR-34a-5p | SLC1A5 | 0.000159223 | 3.38E-71 |
| AP002358.1 | hsa-miR-7-5p, hsa-miR-34a-5p | EFHD2 | 0.027296867 | 6.66E-09 |
| AP002358.1 | hsa-miR-7-5p | IGSF3 | 0.014116943 | 3.62E-36 |
| AP002358.1 | hsa-miR-204-5p | ELOVL6 | 0.009540129 | 9.86E-22 |
| AP002358.1 | hsa-miR-20b-5p | SCD | 0.000220506 | 1.47E-75 |
| AP002358.1 | hsa-miR-204-5p | PLAUR | 0.000162353 | 3.90E-86 |
| AP002358.1 | hsa-miR-7-5p | DSC2 | 0.00027994 | 2.11E-87 |
| AP002358.1 | hsa-miR-146b-5p, hsa-miR-20b-5p, hsa-miR-363-3p | CDKN1A | 0.006022704 | 2.30E-08 |
| AP002358.1 | hsa-miR-205-5p | SLC7A2 | 0.002084698 | 1.68E-08 |
| AP002358.1 | hsa-miR-7-5p | ROR1 | 0.000441322 | 1.65E-69 |
| AP002358.1 | hsa-miR-20b-5p | RUNX3 | 0.000308752 | 9.41E-13 |
| AP002358.1 | hsa-miR-34a-5p | ZAP70 | 0.002475774 | 1.69E-11 |
| AC018816.1 | hsa-miR-204-5p, hsa-miR-34a-5p | HMGA2 | 0.019522737 | 2.66E-73 |
| AC018816.1 | hsa-miR-204-5p | FAXC | 0.013102616 | 2.49E-64 |
| AC018816.1 | hsa-miR-34a-5p | GALNT7 | 0.00265899 | 4.44E-64 |
| AC018816.1 | hsa-miR-199b-5p | PLXND1 | 0.001037701 | 3.09E-46 |
| AC018816.1 | hsa-miR-138-5p, hsa-miR-34a-5p | CCND1 | 0.000650722 | 1.05E-37 |
| AC018816.1 | hsa-miR-204-5p | BID | 0.011853229 | 7.23E-128 |
| AC018816.1 | hsa-miR-204-5p | SAMD1 | 0.002954935 | 2.40E-90 |
| AC018816.1 | hsa-miR-138-5p | LCN2 | 0.008464794 | 5.91E-56 |
| AC018816.1 | hsa-miR-205-5p | XPR1 | 0.018243574 | 1.24E-32 |
| AC018816.1 | hsa-miR-204-5p | CDH4 | 0.003593098 | 1.84E-38 |
| AC018816.1 | hsa-miR-138-5p | SEMA4C | 0.000989757 | 1.62E-58 |
| AC018816.1 | hsa-miR-204-5p | SH2D4A | 0.008615666 | 2.48E-46 |
| AC018816.1 | hsa-miR-204-5p | MDFI | 0.036224181 | 6.13E-30 |
| AC018816.1 | hsa-miR-204-5p | FSTL4 | 0.045967233 | 5.25E-10 |
| AC018816.1 | hsa-miR-204-5p | PLAG1 | 0.031215143 | 2.04E-45 |
| AC018816.1 | hsa-miR-34a-5p | BAX | 0.006720291 | 1.51E-58 |
| AC018816.1 | hsa-miR-204-5p | B4GALNT3 | 0.000541274 | 2.25E-39 |
| AC018816.1 | hsa-miR-138-5p | S100A1 | 0.033450748 | 5.05E-58 |
| AC018816.1 | hsa-miR-204-5p, hsa-miR-34a-5p | HRK | 0.000170889 | 1.38E-11 |
| AC018816.1 | hsa-miR-199b-5p | TSC22D1 | 0.006720291 | 1.25E-53 |
| AC018816.1 | hsa-miR-204-5p | PI4K2A | 0.003593098 | 3.96E-49 |
| AC018816.1 | hsa-miR-138-5p, hsa-miR-204-5p | MEX3A | 0.044042889 | 3.09E-26 |
| AC018816.1 | hsa-miR-204-5p | IGFBP2 | 0.033450748 | 6.17E-21 |
| AC018816.1 | hsa-miR-204-5p | ALPL | 0.008464794 | 5.47E-13 |
| AC018816.1 | hsa-miR-34a-5p | EFHD2 | 0.004560105 | 1.88E-54 |
| AC018816.1 | hsa-miR-204-5p | MYRF | 0.024191056 | 7.70E-11 |
| AC018816.1 | hsa-miR-204-5p | LRRC4 | 0.00033175 | 0.015992445 |
| AC018816.1 | hsa-miR-204-5p | ELOVL6 | 0.045967233 | 7.85E-10 |
| AC018816.1 | hsa-miR-204-5p, hsa-miR-205-5p | NOX5 | 0.001668869 | 0.013132243 |
| AC018816.1 | hsa-miR-204-5p | FAM167B | 0.011853229 | 1.03E-13 |
| AC018816.1 | hsa-miR-204-5p | PLAUR | 0.049772914 | 6.82E-59 |
| AC018816.1 | hsa-miR-199b-5p | SNAP25 | 0.018243574 | 2.46E-37 |
| AC018816.1 | hsa-miR-204-5p | CREB5 | 0.000235542 | 4.62E-50 |
| AC018816.1 | hsa-miR-204-5p | STEAP4 | 0.010722613 | 3.90E-14 |
| AC018816.1 | hsa-miR-204-5p | CXCL8 | 0.036224181 | 5.34E-22 |
| AC018816.1 | hsa-miR-205-5p | SLC7A2 | 0.005935856 | 2.69E-08 |
| AC018816.1 | hsa-miR-204-5p | ARNTL2 | 0.011853229 | 7.66E-20 |
| AC018816.1 | hsa-miR-34a-5p | ZAP70 | 0.000406329 | 3.65E-06 |
| LINC01759 | hsa-miR-150-5p | SRCIN1 | 0.028472489 | 4.33E-36 |
| LINC01759 | hsa-miR-150-5p | FAXC | 0.043093497 | 5.20E-31 |
| LINC01759 | hsa-miR-150-5p | MANEAL | 0.026163909 | 5.97E-27 |
| LINC01759 | hsa-miR-150-5p | XPR1 | 0.009619084 | 3.38E-12 |
| LINC01759 | hsa-miR-150-5p | HIP1 | 0.028472489 | 4.86E-10 |
| LINC01759 | hsa-miR-150-5p | MAPK13 | 0.010388611 | 8.10E-14 |
| LINC01759 | hsa-miR-150-5p | KCNK5 | 0.03655252 | 1.60E-08 |
| LINC01759 | hsa-miR-150-5p | NOTCH3 | 0.00346287 | 9.91E-06 |
| LINC01759 | hsa-miR-150-5p | GJC1 | 0.018853405 | 0.002584833 |
| LINC01759 | hsa-miR-150-5p | EMP2 | 0.032320123 | 9.21E-10 |
| LINC01759 | hsa-miR-150-5p | C3orf36 | 0.025394382 | 0.002368121 |
| LINC01759 | hsa-miR-150-5p | PTPRR | 0.000384763 | 0.000106599 |
| AC090673.1 | hsa-miR-9-5p | CDH3 | 0.003712624 | 2.21E-54 |
| AC090673.1 | hsa-miR-9-5p | P4HA2 | 0.001267142 | 4.02E-30 |
| AC090673.1 | hsa-miR-150-5p, hsa-miR-20b-5p | ZMAT3 | 0.004749505 | 1.17E-22 |
| AC090673.1 | hsa-miR-9-5p | KCNJ2 | 0.002693654 | 5.48E-36 |
| AC090673.1 | hsa-miR-150-5p, hsa-miR-20b-5p | DRAXIN | 0.001544398 | 4.30E-27 |
| AC090673.1 | hsa-miR-204-5p | BID | 0.032524987 | 2.45E-35 |
| AC090673.1 | hsa-miR-150-5p, hsa-miR-20b-5p | MANEAL | 0.00250659 | 7.01E-18 |
| AC090673.1 | hsa-miR-214-3p | JAG2 | 0.006851067 | 1.69E-14 |
| AC090673.1 | hsa-miR-363-3p | TMEM41A | 0.000142763 | 7.16E-38 |
| AC090673.1 | hsa-miR-204-5p | SAMD1 | 0.04728351 | 1.01E-20 |
| AC090673.1 | hsa-miR-138-5p | LCN2 | 0.035782993 | 4.86E-16 |
| AC090673.1 | hsa-miR-214-3p | AHNAK2 | 0.002009701 | 2.33E-30 |
| AC090673.1 | hsa-miR-7-5p | TRIM47 | 0.035782993 | 1.32E-18 |
| AC090673.1 | hsa-miR-20b-5p | ITGA2 | 0.035304895 | 2.57E-28 |
| AC090673.1 | hsa-miR-20b-5p | LIMK1 | 0.000408765 | 1.23E-06 |
| AC090673.1 | hsa-miR-20b-5p | PSD3 | 0.01369328 | 1.14E-34 |
| AC090673.1 | hsa-miR-204-5p | CDH4 | 0.005940621 | 4.09E-29 |
| AC090673.1 | hsa-miR-204-5p | SH2D4A | 0.020957022 | 1.92E-43 |
| AC090673.1 | hsa-miR-138-5p | LPL | 0.000273664 | 2.39E-08 |
| AC090673.1 | hsa-miR-204-5p | CD44 | 0.003018375 | 3.55E-50 |
| AC090673.1 | hsa-miR-150-5p, hsa-miR-20b-5p | HIP1 | 0.004284512 | 2.10E-17 |
| AC090673.1 | hsa-miR-9-5p | RAB34 | 0.006437488 | 3.89E-44 |
| AC090673.1 | hsa-miR-363-3p | PLXNA3 | 0.021228181 | 1.53E-55 |
| AC090673.1 | hsa-miR-150-5p, hsa-miR-20b-5p | QSOX1 | 0.004801094 | 2.84E-23 |
| AC090673.1 | hsa-miR-363-3p | TOR4A | 0.00360843 | 9.40E-31 |
| AC090673.1 | hsa-miR-214-3p | LZTS1 | 0.001267142 | 2.99E-06 |
| AC090673.1 | hsa-miR-7-5p | GLDN | 0.000168356 | 1.84E-16 |
| AC090673.1 | hsa-miR-7-5p, hsa-miR-363-3p | PMEPA1 | 0.004463549 | 2.03E-11 |
| AC090673.1 | hsa-miR-214-3p | TRIM29 | 0.004793227 | 2.28E-12 |
| AC090673.1 | hsa-miR-150-5p | NOTCH3 | 0.003186274 | 5.47E-09 |
| AC090673.1 | hsa-miR-138-5p, hsa-miR-181a-5p | S100A1 | 0.000172987 | 2.37E-11 |
| AC090673.1 | hsa-miR-7-5p | SSX1 | 0.035782993 | 3.20E-14 |
| AC090673.1 | hsa-miR-7-5p, hsa-miR-33b-5p | TMEM98 | 0.032524987 | 2.39E-31 |
| AC090673.1 | hsa-miR-150-5p, hsa-miR-20b-5p | RTN2 | 0.000447121 | 3.47E-21 |
| AC090673.1 | hsa-miR-9-5p, hsa-miR-204-5p | PI4K2A | 0.000418306 | 1.24E-11 |
| AC090673.1 | hsa-miR-20b-5p | TMEM100 | 0.000893392 | 3.68E-12 |
| AC090673.1 | hsa-miR-9-5p | TGFBI | 0.041743746 | 3.26E-10 |
| AC090673.1 | hsa-miR-150-5p | PLXDC1 | 0.004793227 | 7.49E-13 |
| AC090673.1 | hsa-miR-7-5p | TGM2 | 0.00139894 | 2.98E-05 |
| AC090673.1 | hsa-miR-9-5p | RHOV | 0.000418306 | 4.31E-06 |
| AC090673.1 | hsa-miR-20b-5p | FOXQ1 | 0.000579846 | 4.74E-37 |
| AC090673.1 | hsa-miR-150-5p, hsa-miR-20b-5p | SLC1A5 | 0.002954585 | 2.02E-16 |
| AC090673.1 | hsa-miR-214-3p | ASF1B | 0.003186274 | 4.54E-20 |
| AC090673.1 | hsa-miR-20b-5p | PCNX2 | 0.01139247 | 2.98E-20 |
| AC090673.1 | hsa-miR-204-5p | ELOVL6 | 0.014239948 | 5.27E-06 |
| AC090673.1 | hsa-miR-138-5p | CASTOR2 | 0.004782578 | 3.59E-17 |
| AC090673.1 | hsa-miR-9-5p | TM4SF1 | 0.037111829 | 8.00E-07 |
| AC090673.1 | hsa-miR-9-5p | NTRK3 | 0.00806967 | 9.95E-24 |
| AC090673.1 | hsa-miR-204-5p | PLAUR | 0.000820637 | 6.05E-10 |
| AC090673.1 | hsa-miR-204-5p | CREB5 | 0.001952811 | 5.28E-17 |
| AC090673.1 | hsa-miR-9-5p, hsa-miR-144-3p, hsa-miR-205-5p | SLC7A2 | 0.000893392 | 1.15E-08 |
| AC090673.1 | hsa-miR-7-5p, hsa-miR-181a-5p | RGS5 | 0.001504541 | 0.010027363 |
| AC090673.1 | hsa-miR-7-5p | ROR1 | 0.002180452 | 3.74E-07 |
| AC090673.1 | hsa-miR-7-5p, hsa-miR-214-3p | PAPPA | 0.000411465 | 1.92E-05 |
| AC090673.1 | hsa-miR-9-5p | LOX | 0.000142763 | 0.031140852 |
| AC090673.1 | hsa-miR-9-5p, hsa-miR-20b-5p | ESR1 | 0.00360843 | 3.50E-05 |
| AL031985.3 | hsa-miR-9-5p | CDH3 | 0.043241226 | 8.12E-114 |
| AL031985.3 | hsa-miR-9-5p | P4HA2 | 0.000208228 | 5.47E-57 |
| AL031985.3 | hsa-miR-9-5p, hsa-miR-183-5p | CCND1 | 0.025516199 | 1.95E-52 |
| AL031985.3 | hsa-miR-144-3p | MET | 0.003187806 | 1.08E-97 |
| AL031985.3 | hsa-miR-204-5p | BID | 0.002769958 | 2.16E-127 |
| AL031985.3 | hsa-miR-204-5p | SAMD1 | 0.008701529 | 5.93E-46 |
| AL031985.3 | hsa-miR-205-5p | XPR1 | 0.000394685 | 2.76E-83 |
| AL031985.3 | hsa-miR-204-5p | CDH4 | 0.010537618 | 1.96E-57 |
| AL031985.3 | hsa-miR-146b-5p, hsa-miR-204-5p | IL1RAP | 0.000439128 | 1.50E-129 |
| AL031985.3 | hsa-miR-204-5p | SH2D4A | 0.024659063 | 2.02E-59 |
| AL031985.3 | hsa-miR-144-3p | TGFB1 | 0.000282148 | 4.61E-52 |
| AL031985.3 | hsa-miR-363-3p | PLXNA3 | 0.000827294 | 1.89E-55 |
| AL031985.3 | hsa-miR-363-3p | TOR4A | 0.000501182 | 1.43E-45 |
| AL031985.3 | hsa-miR-181a-5p, hsa-miR-204-5p | TGFBR1 | 0.00073351 | 2.65E-126 |
| AL031985.3 | hsa-miR-204-5p | CDH2 | 0.004723385 | 3.16E-07 |
| AL031985.3 | hsa-miR-181a-5p | BAX | 0.019390668 | 2.96E-25 |
| AL031985.3 | hsa-miR-363-3p | PMEPA1 | 0.000166093 | 3.57E-53 |
| AL031985.3 | hsa-miR-9-5p, hsa-miR-204-5p | PI4K2A | 0.000439128 | 4.05E-38 |
| AL031985.3 | hsa-miR-9-5p | TGFBI | 0.040013009 | 9.04E-27 |
| AL031985.3 | hsa-miR-144-3p | GRIK3 | 0.040890515 | 1.79E-07 |
| AL031985.3 | hsa-miR-204-5p | ALPL | 0.014621008 | 2.51E-07 |
| AL031985.3 | hsa-miR-182-5p, hsa-miR-204-5p | LRRC4 | 0.001703102 | 2.03E-05 |
| AL031985.3 | hsa-miR-204-5p | ELOVL6 | 0.02097296 | 1.29E-29 |
| AL031985.3 | hsa-miR-181a-5p | MMP14 | 0.010537618 | 1.31E-38 |
| AL031985.3 | hsa-miR-181a-5p, hsa-miR-181b-5p, hsa-miR-181d-5p | SCD | 0.013679104 | 2.47E-57 |
| AL031985.3 | hsa-miR-204-5p | PLAUR | 0.003009705 | 7.28E-56 |
| AL031985.3 | hsa-miR-182-5p, hsa-miR-204-5p | CREB5 | 0.002009296 | 2.55E-75 |
| AL031985.3 | hsa-miR-204-5p | STEAP4 | 0.03044269 | 1.30E-18 |
| AL031985.3 | hsa-miR-9-5p, hsa-miR-144-3p, hsa-miR-205-5p | SLC7A2 | 0.000406699 | 7.70E-25 |
| AL031985.3 | hsa-miR-146b-5p, hsa-miR-181a-5p, hsa-miR-181b-5p, hsa-miR-181d-5p | PMAIP1 | 0.000184188 | 2.52E-33 |
| AL031985.3 | hsa-miR-181a-5p, hsa-miR-181b-5p, hsa-miR-181d-5p | ARSJ | 0.000158019 | 2.52E-41 |
| AL031985.3 | hsa-miR-9-5p, hsa-miR-182-5p | LOX | 0.000335038 | 1.13E-26 |
| NR2F1-AS1 | hsa-miR-150-5p | ZMAT3 | 0.001392325 | 3.14E-54 |
| NR2F1-AS1 | hsa-miR-9-5p | KCNJ2 | 0.000744913 | 1.26E-94 |
| NR2F1-AS1 | hsa-miR-138-5p | LCN2 | 0.026933436 | 1.80E-89 |
| NR2F1-AS1 | hsa-miR-150-5p | XPR1 | 0.000428835 | 5.07E-65 |
| NR2F1-AS1 | hsa-miR-146b-5p | IL1RAP | 0.033618353 | 6.46E-107 |
| NR2F1-AS1 | hsa-miR-34a-5p | CD44 | 0.038353408 | 1.99E-16 |
| NR2F1-AS1 | hsa-miR-363-3p | PLXNA3 | 0.004700984 | 2.25E-61 |
| NR2F1-AS1 | hsa-miR-150-5p, hsa-miR-199b-5p | QSOX1 | 0.000114688 | 8.83E-104 |
| NR2F1-AS1 | hsa-miR-150-5p | MAPK13 | 0.00062485 | 4.98E-60 |
| NR2F1-AS1 | hsa-miR-363-3p | TOR4A | 0.002774978 | 1.57E-39 |
| NR2F1-AS1 | hsa-miR-363-3p | PMEPA1 | 0.004490358 | 3.34E-71 |
| NR2F1-AS1 | hsa-miR-150-5p | NOTCH3 | 0.001399637 | 4.71E-20 |
| NR2F1-AS1 | hsa-miR-9-5p | PRDM1 | 0.000428835 | 6.45E-24 |
| NR2F1-AS1 | hsa-miR-9-5p | PXDN | 0.00062485 | 1.13E-08 |
| NR2F1-AS1 | hsa-miR-34a-5p | TGM2 | 0.013760399 | 1.06E-82 |
| NR2F1-AS1 | hsa-miR-9-5p | RHOV | 0.033618353 | 3.77E-56 |
| NR2F1-AS1 | hsa-miR-138-5p, hsa-miR-182-5p | CASTOR2 | 0.000314254 | 2.02E-29 |
| NR2F1-AS1 | hsa-miR-150-5p | MMP14 | 0.000138176 | 9.09E-61 |
| NR2F1-AS1 | hsa-miR-9-5p | TM4SF1 | 0.002356995 | 1.13E-83 |
| NR2F1-AS1 | hsa-miR-146b-5p, hsa-miR-182-5p, hsa-miR-363-3p | CDKN1A | 0.00027407 | 1.15E-18 |
| NR2F1-AS1 | hsa-miR-182-5p | CREB5 | 0.017863114 | 1.15E-115 |
| NR2F1-AS1 | hsa-miR-363-3p | FAM129A | 0.000152508 | 9.33E-35 |
| NR2F1-AS1 | hsa-miR-34a-5p | ZAP70 | 0.004143543 | 3.18E-13 |
| TNRC6C-AS1 | hsa-miR-150-5p | ZMAT3 | 0.004954845 | 1.32E-52 |
| TNRC6C-AS1 | hsa-miR-96-5p, hsa-miR-34a-5p | CCND1 | 0.000787074 | 8.97E-59 |
| TNRC6C-AS1 | hsa-miR-150-5p | TMEM92 | 0.007392181 | 5.72E-106 |
| TNRC6C-AS1 | hsa-miR-7-5p | TRIM47 | 0.015005771 | 5.04E-101 |
| TNRC6C-AS1 | hsa-miR-7-5p | RNF183 | 0.04436199 | 3.06E-48 |
| TNRC6C-AS1 | hsa-miR-7-5p | FNDC4 | 0.029792059 | 3.65E-64 |
| TNRC6C-AS1 | hsa-miR-34a-5p | CD44 | 0.019508336 | 3.74E-53 |
| TNRC6C-AS1 | hsa-miR-7-5p | SSX1 | 0.015005771 | 1.81E-31 |
| TNRC6C-AS1 | hsa-miR-199b-5p | TSC22D1 | 0.001255388 | 4.60E-69 |
| TNRC6C-AS1 | hsa-miR-7-5p, hsa-miR-199b-5p | LAMC2 | 0.000166202 | 3.08E-30 |
| TNRC6C-AS1 | hsa-miR-7-5p, hsa-miR-34a-5p | TGM2 | 0.004394775 | 4.62E-41 |
| TNRC6C-AS1 | hsa-miR-7-5p, hsa-miR-96-5p, hsa-miR-34a-5p | EFHD2 | 0.034023174 | 2.02E-36 |
| TNRC6C-AS1 | hsa-miR-7-5p | DSC2 | 0.004394775 | 2.67E-27 |
| TNRC6C-AS1 | hsa-miR-182-5p | CREB5 | 0.002215579 | 1.92E-66 |
| TNRC6C-AS1 | hsa-miR-7-5p | ROR1 | 0.005804299 | 6.21E-26 |
| TNRC6C-AS1 | hsa-miR-7-5p | PAPPA | 0.03194961 | 2.11E-22 |
| TNRC6C-AS1 | hsa-miR-96-5p, hsa-miR-182-5p | LOX | 0.005086082 | 4.91E-14 |
| TNRC6C-AS1 | hsa-miR-34a-5p | ZAP70 | 0.001292018 | 8.05E-11 |
| DOCK9-AS2 | hsa-miR-199b-5p | PLXND1 | 0.033333983 | 3.67E-29 |
| DOCK9-AS2 | hsa-miR-150-5p, hsa-miR-375 | XPR1 | 0.002401324 | 6.25E-37 |
| DOCK9-AS2 | hsa-miR-199b-5p | ITGA3 | 0.035930648 | 9.90E-80 |
| DOCK9-AS2 | hsa-miR-150-5p, hsa-miR-199b-5p | QSOX1 | 0.003445208 | 1.04E-81 |
| DOCK9-AS2 | hsa-miR-150-5p | NOTCH3 | 0.027405998 | 1.75E-08 |
| DOCK9-AS2 | hsa-miR-199b-5p | NAB2 | 0.009433552 | 1.95E-112 |
| DOCK9-AS2 | hsa-miR-199b-5p | TSC22D1 | 0.000853541 | 5.44E-87 |
| DOCK9-AS2 | hsa-miR-150-5p, hsa-miR-375 | RTN2 | 0.016113913 | 7.17E-57 |
| DOCK9-AS2 | hsa-miR-199b-5p | LAMC2 | 0.000230082 | 8.08E-23 |
| DOCK9-AS2 | hsa-miR-375 | TGM2 | 0.021373253 | 5.26E-19 |
| DOCK9-AS2 | hsa-miR-199b-5p | PLEKHG2 | 0.031439808 | 2.82E-19 |
| DOCK9-AS2 | hsa-miR-375 | SDC1 | 0.048265144 | 0.026148993 |
| DOCK9-AS2 | hsa-miR-199b-5p | SNAP25 | 0.002401324 | 7.52E-37 |
| DOCK9-AS2 | hsa-miR-221-3p, hsa-miR-222-3p | ESR1 | 0.019023644 | 5.37E-21 |
| LINC00511 | hsa-miR-9-5p | P4HA2 | 0.000654897 | 3.58E-26 |
| LINC00511 | hsa-miR-9-5p | KCNJ2 | 0.005182852 | 3.24E-74 |
| LINC00511 | hsa-miR-150-5p | TMEM92 | 0.020970465 | 8.24E-120 |
| LINC00511 | hsa-miR-31-5p | MET | 0.000673865 | 1.90E-75 |
| LINC00511 | hsa-miR-7-5p, hsa-miR-150-5p, hsa-miR-205-5p, hsa-miR-375 | XPR1 | 0.003393699 | 1.26E-76 |
| LINC00511 | hsa-miR-7-5p | TRIM47 | 0.025779146 | 6.54E-54 |
| LINC00511 | hsa-miR-20b-5p | LIMK1 | 0.000293919 | 8.52E-57 |
| LINC00511 | hsa-miR-9-5p | RUNX1 | 0.000248653 | 5.85E-115 |
| LINC00511 | hsa-miR-375 | IL1RAP | 0.002330651 | 3.84E-82 |
| LINC00511 | hsa-miR-199b-5p | ITGA3 | 0.043583782 | 6.76E-83 |
| LINC00511 | hsa-miR-7-5p | FNDC4 | 0.000654897 | 4.64E-55 |
| LINC00511 | hsa-miR-9-5p | RAB34 | 0.010454584 | 2.69E-51 |
| LINC00511 | hsa-miR-9-5p | ENDOD1 | 0.003933744 | 1.66E-59 |
| LINC00511 | hsa-miR-7-5p | GLDN | 0.000581625 | 6.31E-26 |
| LINC00511 | hsa-miR-9-5p | PXDN | 0.004529282 | 2.25E-12 |
| LINC00511 | hsa-miR-7-5p | SSX1 | 0.025779146 | 9.05E-21 |
| LINC00511 | hsa-miR-9-5p | PI4K2A | 0.030992676 | 2.35E-30 |
| LINC00511 | hsa-miR-9-5p | TGFBI | 0.002080088 | 6.88E-41 |
| LINC00511 | hsa-miR-9-5p, hsa-miR-375 | FSTL3 | 0.002859406 | 4.52E-73 |
| LINC00511 | hsa-miR-7-5p, hsa-miR-375 | TGM2 | 0.000532486 | 1.47E-85 |
| LINC00511 | hsa-miR-20b-5p | DCBLD2 | 0.000179449 | 2.03E-53 |
| LINC00511 | hsa-miR-9-5p, hsa-miR-375 | SDC1 | 0.000581625 | 9.66E-09 |
| LINC00511 | hsa-miR-20b-5p | SCD | 0.001734407 | 4.63E-62 |
| LINC00511 | hsa-miR-503-5p, hsa-miR-20b-5p | CDKN1A | 0.000141401 | 4.04E-23 |
| LINC00511 | hsa-miR-9-5p, hsa-miR-205-5p | SLC7A2 | 0.000864544 | 3.08E-06 |
| LINC00511 | hsa-miR-20b-5p | PMAIP1 | 0.001072641 | 1.70E-62 |
| LINC00511 | hsa-miR-7-5p | ROR1 | 0.000836286 | 9.74E-55 |
| LINC00511 | hsa-miR-375 | ARNTL2 | 0.013640349 | 2.08E-71 |
| LINC00511 | hsa-miR-20b-5p, hsa-miR-375 | RUNX3 | 0.00020048 | 3.35E-20 |
| AC105285.1 | hsa-miR-150-5p | FAXC | 0.00446783 | 3.94E-37 |
| AC105285.1 | hsa-miR-150-5p | TMEM92 | 0.018097982 | 3.34E-29 |
| AC105285.1 | hsa-miR-551b-3p | PDE4C | 0.040026547 | 5.29E-32 |
| AC105285.1 | hsa-miR-138-5p | MTHFD1L | 0.036156779 | 9.66E-24 |
| AC105285.1 | hsa-miR-138-5p | LCN2 | 0.023855329 | 1.16E-25 |
| AC105285.1 | hsa-miR-138-5p | SEMA4C | 0.001679426 | 5.15E-38 |
| AC105285.1 | hsa-miR-144-3p | TGFB1 | 0.001851133 | 7.62E-25 |
| AC105285.1 | hsa-miR-150-5p, hsa-miR-199b-5p | QSOX1 | 0.000191013 | 3.91E-23 |
| AC105285.1 | hsa-miR-138-5p | SOX4 | 0.000451634 | 4.67E-11 |
| AC105285.1 | hsa-miR-138-5p | CDH2 | 0.018313541 | 1.45E-11 |
| AC105285.1 | hsa-miR-138-5p | S100A1 | 0.003258039 | 4.32E-36 |
| AC105285.1 | hsa-miR-150-5p | PLXDC1 | 0.00106871 | 2.62E-14 |
| AC105285.1 | hsa-miR-144-3p | NEURL1B | 0.016688147 | 0.00018363 |
| AC105285.1 | hsa-miR-96-5p, hsa-miR-183-5p | EFHD2 | 0.003116039 | 4.41E-17 |
| AC105285.1 | hsa-miR-144-3p, hsa-miR-205-5p | SLC7A2 | 0.003848038 | 4.09E-20 |
| AC105285.1 | hsa-miR-150-5p | PTPRR | 0.023855329 | 0.024198732 |
| AC105285.1 | hsa-miR-144-3p | PBX3 | 0.003118524 | 8.77E-14 |
| AC105285.1 | hsa-miR-96-5p, hsa-miR-182-5p | LOX | 0.002182 | 0.003222271 |
| AC120036.5 | hsa-miR-138-5p, hsa-miR-183-5p | CCND1 | 0.04977152 | 1.16E-52 |
| AC120036.5 | hsa-miR-204-5p | BID | 0.042079099 | 2.37E-39 |
| AC120036.5 | hsa-miR-204-5p | SAMD1 | 0.011062659 | 4.24E-22 |
| AC120036.5 | hsa-miR-138-5p | LCN2 | 0.016544825 | 3.03E-32 |
| AC120036.5 | hsa-miR-204-5p | CDH4 | 0.013379924 | 1.39E-25 |
| AC120036.5 | hsa-miR-181a-5p | RUNX1 | 0.00441488 | 4.28E-28 |
| AC120036.5 | hsa-miR-146b-5p, hsa-miR-204-5p | IL1RAP | 0.000634968 | 8.24E-41 |
| AC120036.5 | hsa-miR-138-5p | SEMA4C | 0.012015228 | 8.91E-44 |
| AC120036.5 | hsa-miR-204-5p | SH2D4A | 0.031074112 | 1.05E-56 |
| AC120036.5 | hsa-miR-181a-5p | BAX | 0.024496609 | 2.63E-13 |
| AC120036.5 | hsa-miR-204-5p | HRK | 0.022268096 | 8.08E-16 |
| AC120036.5 | hsa-miR-204-5p | PI4K2A | 0.000634968 | 8.60E-25 |
| AC120036.5 | hsa-miR-144-3p | NEURL1B | 0.004607308 | 9.24E-05 |
| AC120036.5 | hsa-miR-204-5p, hsa-miR-21-5p | EPHA4 | 0.032324329 | 3.93E-40 |
| AC120036.5 | hsa-miR-181a-5p | MMP14 | 0.000634968 | 0.000465784 |
| AC120036.5 | hsa-miR-204-5p | PLAUR | 0.003846023 | 5.21E-14 |
| AC120036.5 | hsa-miR-146b-5p, hsa-miR-181a-5p, hsa-miR-182-5p | CDKN1A | 0.039334759 | 7.65E-08 |
| AC120036.5 | hsa-miR-204-5p | STEAP4 | 0.038266564 | 1.38E-14 |
| AC120036.5 | hsa-miR-204-5p | CXCL8 | 0.002605738 | 7.59E-11 |
| AC120036.5 | hsa-miR-144-3p, hsa-miR-181a-5p, hsa-miR-181b-5p | PBX3 | 0.001075424 | 0.002127869 |
| AC120036.5 | hsa-miR-204-5p | ARNTL2 | 0.042079099 | 5.14E-12 |
| AC120036.5 | hsa-miR-181a-5p, hsa-miR-181b-5p, hsa-miR-181d-5p | ARSJ | 0.000287772 | 1.72E-14 |
| CYP1B1-AS1 | hsa-miR-204-5p, hsa-miR-33b-5p | HMGA2 | 0.002538357 | 1.11E-72 |
| CYP1B1-AS1 | hsa-miR-150-5p, hsa-miR-204-5p | FAXC | 0.015810427 | 1.63E-63 |
| CYP1B1-AS1 | hsa-miR-214-3p | GALNT7 | 0.045778826 | 5.42E-83 |
| CYP1B1-AS1 | hsa-miR-150-5p | TMEM92 | 0.018097982 | 1.14E-95 |
| CYP1B1-AS1 | hsa-miR-204-5p | BID | 0.00106871 | 3.68E-96 |
| CYP1B1-AS1 | hsa-miR-214-3p | JAG2 | 0.014412842 | 1.82E-14 |
| CYP1B1-AS1 | hsa-miR-204-5p | SAMD1 | 0.02227817 | 4.33E-31 |
| CYP1B1-AS1 | hsa-miR-150-5p, hsa-miR-205-5p | XPR1 | 0.020462895 | 2.02E-37 |
| CYP1B1-AS1 | hsa-miR-214-3p | AHNAK2 | 0.000431302 | 5.94E-57 |
| CYP1B1-AS1 | hsa-miR-204-5p | CDH4 | 0.001861609 | 3.42E-25 |
| CYP1B1-AS1 | hsa-miR-204-5p | SH2D4A | 0.006926777 | 3.55E-43 |
| CYP1B1-AS1 | hsa-miR-150-5p, hsa-miR-199b-5p | QSOX1 | 0.000191013 | 5.47E-68 |
| CYP1B1-AS1 | hsa-miR-204-5p | TGFBR1 | 0.000158269 | 4.58E-106 |
| CYP1B1-AS1 | hsa-miR-214-3p | LZTS1 | 0.047150544 | 6.01E-22 |
| CYP1B1-AS1 | hsa-miR-204-5p | PLAG1 | 0.000113755 | 1.28E-66 |
| CYP1B1-AS1 | hsa-miR-214-3p | TRIM29 | 0.011036459 | 5.01E-39 |
| CYP1B1-AS1 | hsa-miR-150-5p | NOTCH3 | 0.000980691 | 2.09E-15 |
| CYP1B1-AS1 | hsa-miR-150-5p, hsa-miR-214-3p | TNFSF15 | 0.001147729 | 5.79E-26 |
| CYP1B1-AS1 | hsa-miR-199b-5p | TSC22D1 | 0.04816485 | 5.88E-89 |
| CYP1B1-AS1 | hsa-miR-204-5p | PI4K2A | 0.001861609 | 9.38E-27 |
| CYP1B1-AS1 | hsa-miR-21-5p | TGFBI | 0.001556796 | 5.67E-21 |
| CYP1B1-AS1 | hsa-miR-150-5p | PLXDC1 | 0.00106871 | 4.52E-20 |
| CYP1B1-AS1 | hsa-miR-204-5p | ALPL | 0.023855329 | 2.74E-06 |
| CYP1B1-AS1 | hsa-miR-214-3p | ASF1B | 0.000980691 | 2.11E-17 |
| CYP1B1-AS1 | hsa-miR-204-5p | MYRF | 0.030394259 | 4.36E-21 |
| CYP1B1-AS1 | hsa-miR-204-5p | ELOVL6 | 0.015368367 | 7.90E-14 |
| CYP1B1-AS1 | hsa-miR-204-5p, hsa-miR-21-5p | EPHA4 | 0.007542925 | 2.98E-48 |
| CYP1B1-AS1 | hsa-miR-204-5p | STEAP4 | 0.009541297 | 5.70E-23 |
| CYP1B1-AS1 | hsa-miR-205-5p | SLC7A2 | 0.021067157 | 2.18E-10 |
| CYP1B1-AS1 | hsa-miR-21-5p | CCR7 | 0.003258039 | 0.000175451 |
| CYP1B1-AS1 | hsa-miR-214-3p | PAPPA | 0.009541297 | 6.97E-31 |
| AL138756.1 | hsa-miR-9-5p | P4HA2 | 0.000912591 | 4.01E-19 |
| AL138756.1 | hsa-miR-150-5p | ZMAT3 | 0.029574407 | 1.96E-34 |
| AL138756.1 | hsa-miR-9-5p | KCNJ2 | 0.001296968 | 2.60E-07 |
| AL138756.1 | hsa-miR-214-3p | JAG2 | 0.003812873 | 6.88E-30 |
| AL138756.1 | hsa-miR-138-5p | MTHFD1L | 0.013333105 | 3.35E-33 |
| AL138756.1 | hsa-miR-138-5p | LCN2 | 0.030396306 | 3.16E-20 |
| AL138756.1 | hsa-miR-150-5p, hsa-miR-205-5p | XPR1 | 0.000753142 | 4.33E-12 |
| AL138756.1 | hsa-miR-146b-5p | IL1RAP | 0.042006775 | 0.0003965 |
| AL138756.1 | hsa-miR-138-5p | LPL | 0.035049339 | 1.34E-10 |
| AL138756.1 | hsa-miR-9-5p | RAB34 | 0.018347291 | 2.26E-09 |
| AL138756.1 | hsa-miR-363-3p | PLXNA3 | 0.00912811 | 5.67E-10 |
| AL138756.1 | hsa-miR-138-5p, hsa-miR-363-3p | SOX4 | 0.000108423 | 4.64E-13 |
| AL138756.1 | hsa-miR-363-3p | TOR4A | 0.000251606 | 5.57E-19 |
| AL138756.1 | hsa-miR-363-3p | PMEPA1 | 0.008128118 | 3.66E-18 |
| AL138756.1 | hsa-miR-214-3p | TRIM29 | 0.002644888 | 2.67E-08 |
| AL138756.1 | hsa-miR-150-5p | NOTCH3 | 0.001990208 | 3.69E-08 |
| AL138756.1 | hsa-miR-138-5p, hsa-miR-181a-5p | S100A1 | 0.00526146 | 2.57E-22 |
| AL138756.1 | hsa-miR-214-3p | RAB15 | 0.021228181 | 7.03E-09 |
| AL138756.1 | hsa-miR-9-5p | PXDN | 0.008152074 | 8.18E-17 |
| AL138756.1 | hsa-miR-150-5p, hsa-miR-214-3p | TNFSF15 | 0.018158734 | 6.33E-15 |
| AL138756.1 | hsa-miR-9-5p | PI4K2A | 0.042006775 | 3.11E-31 |
| AL138756.1 | hsa-miR-9-5p, hsa-miR-21-5p | TGFBI | 0.000401023 | 2.26E-08 |
| AL138756.1 | hsa-miR-150-5p | PLXDC1 | 0.002644888 | 9.98E-10 |
| AL138756.1 | hsa-miR-214-3p | ASF1B | 0.001990208 | 1.17E-31 |
| AL138756.1 | hsa-miR-150-5p | C3orf36 | 0.000701798 | 1.41E-06 |
| AL138756.1 | hsa-miR-150-5p | PTPRR | 0.030396306 | 2.25E-05 |
| AL138756.1 | hsa-miR-363-3p | FAM129A | 0.00206014 | 8.88E-05 |
| AL138756.1 | hsa-miR-181a-5p, hsa-miR-181b-5p, hsa-miR-181d-5p | ARSJ | 0.000713771 | 0.03958572 |
| AL138756.1 | hsa-miR-9-5p, hsa-miR-96-5p, hsa-miR-182-5p | LOX | 0.000617069 | 0.000532185 |
| ST7-AS1 | hsa-miR-181a-5p | TGFBR3 | 0.00842536 | 6.57E-17 |
| ST7-AS1 | hsa-miR-181a-5p, hsa-miR-181b-5p, hsa-miR-181d-5p | RGS16 | 0.000169399 | 8.67E-38 |
| ST7-AS1 | hsa-miR-181a-5p | RASSF6 | 0.036945539 | 2.26E-29 |
| ST7-AS1 | hsa-miR-181a-5p | MOB3B | 0.001677394 | 1.48E-18 |
| ST7-AS1 | hsa-miR-181a-5p | ALDH1A1 | 0.024477941 | 5.95E-27 |
| ST7-AS1 | hsa-miR-181a-5p | OCA2 | 0.00897717 | 1.19E-25 |
| ST7-AS1 | hsa-miR-181a-5p, hsa-miR-181b-5p | FOS | 0.005040443 | 6.19E-13 |
| ST7-AS1 | hsa-miR-181a-5p | GPR83 | 0.000437351 | 4.15E-22 |
| HAGLR | hsa-miR-204-5p, hsa-miR-34a-5p | HMGA2 | 0.002021565 | 8.52E-40 |
| HAGLR | hsa-miR-34a-5p | GALNT7 | 0.004160884 | 5.05E-39 |
| HAGLR | hsa-miR-204-5p | BID | 0.003312968 | 1.01E-49 |
| HAGLR | hsa-miR-204-5p | SAMD1 | 0.003316912 | 3.03E-33 |
| HAGLR | hsa-miR-20b-5p | LIMK1 | 0.001178707 | 4.22E-35 |
| HAGLR | hsa-miR-204-5p | CDH4 | 0.004455015 | 9.93E-24 |
| HAGLR | hsa-miR-9-5p | RUNX1 | 0.000230875 | 6.43E-53 |
| HAGLR | hsa-miR-204-5p | IL1RAP | 0.004455015 | 1.30E-42 |
| HAGLR | hsa-miR-204-5p | SH2D4A | 0.0159607 | 3.97E-34 |
| HAGLR | hsa-miR-199b-5p | ITGA3 | 0.04239751 | 6.47E-45 |
| HAGLR | hsa-miR-9-5p | ENDOD1 | 0.008819989 | 1.14E-31 |
| HAGLR | hsa-miR-34a-5p | BAX | 0.001056151 | 1.28E-18 |
| HAGLR | hsa-miR-20b-5p | F2R | 0.000134392 | 4.82E-07 |
| HAGLR | hsa-miR-9-5p, hsa-miR-204-5p | PI4K2A | 0.000281971 | 1.98E-19 |
| HAGLR | hsa-miR-9-5p | TGFBI | 0.004761395 | 8.91E-31 |
| HAGLR | hsa-miR-204-5p | ALPL | 0.032320123 | 0.000353625 |
| HAGLR | hsa-miR-96-5p, hsa-miR-34a-5p | EFHD2 | 0.016303888 | 1.18E-11 |
| HAGLR | hsa-miR-182-5p, hsa-miR-204-5p | LRRC4 | 0.001752569 | 0.009073753 |
| HAGLR | hsa-miR-204-5p | ELOVL6 | 0.009387757 | 1.92E-21 |
| HAGLR | hsa-miR-9-5p | SDC1 | 0.013453383 | 0.000319688 |
| HAGLR | hsa-miR-20b-5p | SCD | 0.000479652 | 8.30E-33 |
| HAGLR | hsa-miR-204-5p | PLAUR | 0.014229201 | 1.38E-46 |
| HAGLR | hsa-miR-182-5p, hsa-miR-204-5p | CREB5 | 0.001146746 | 3.56E-47 |
| HAGLR | hsa-miR-9-5p, hsa-miR-205-5p | SLC7A2 | 0.002823047 | 7.34E-07 |
| HAGLR | hsa-miR-96-5p, hsa-miR-182-5p | RGS2 | 0.002198293 | 0.027478254 |
| HAGLR | hsa-miR-20b-5p | ARSJ | 0.000134392 | 4.64E-34 |
| HAGLR | hsa-miR-9-5p, hsa-miR-96-5p, hsa-miR-182-5p | LOX | 0.000819509 | 1.02E-19 |
| HAGLR | hsa-miR-34a-5p | PDGFRA | 0.012746594 | 0.000177533 |
| HAGLR | hsa-miR-34a-5p | ZAP70 | 0.005937542 | 9.51E-06 |
| SEPT7-AS1 | hsa-miR-375 | TNS3 | 0.042353747 | 1.65E-47 |
| SEPT7-AS1 | hsa-miR-375 | ZFP36L2 | 0.005826264 | 6.39E-34 |
| SEPT7-AS1 | hsa-miR-205-5p, hsa-miR-375 | BCL2 | 0.013013451 | 7.67E-83 |
| SEPT7-AS1 | hsa-miR-375 | SLC7A6 | 0.013404636 | 1.68E-33 |
| SEPT7-AS1 | hsa-miR-205-5p | CYR61 | 0.003155828 | 2.27E-21 |
| SEPT7-AS1 | hsa-miR-205-5p | ESRRG | 0.031912216 | 1.48E-61 |
| SEPT7-AS1 | hsa-miR-375 | NCAM1 | 0.0187232 | 6.80E-17 |
| SEPT7-AS1 | hsa-miR-205-5p | AR | 0.025616204 | 7.47E-35 |
| AL137026.1 | hsa-miR-204-5p | HMGA2 | 0.033005462 | 8.60E-53 |
| AL137026.1 | hsa-miR-150-5p, hsa-miR-204-5p | FAXC | 0.024269814 | 8.55E-63 |
| AL137026.1 | hsa-miR-150-5p | ZMAT3 | 0.047217061 | 1.57E-24 |
| AL137026.1 | hsa-miR-138-5p | CCND1 | 0.00023554 | 2.83E-23 |
| AL137026.1 | hsa-miR-204-5p | BID | 0.005564496 | 3.81E-91 |
| AL137026.1 | hsa-miR-204-5p | SAMD1 | 0.001362629 | 2.36E-24 |
| AL137026.1 | hsa-miR-138-5p | LCN2 | 0.005771451 | 6.25E-41 |
| AL137026.1 | hsa-miR-204-5p | CDH4 | 0.001659884 | 6.20E-21 |
| AL137026.1 | hsa-miR-204-5p | SH2D4A | 0.004023064 | 5.17E-35 |
| AL137026.1 | hsa-miR-204-5p | MDFI | 0.017493119 | 2.36E-32 |
| AL137026.1 | hsa-miR-204-5p | CD44 | 0.00674945 | 1.32E-15 |
| AL137026.1 | hsa-miR-204-5p | FSTL4 | 0.022393351 | 6.47E-05 |
| AL137026.1 | hsa-miR-138-5p, hsa-miR-204-5p | CDH2 | 0.007976086 | 9.81E-05 |
| AL137026.1 | hsa-miR-204-5p | PLAG1 | 0.001009328 | 2.40E-51 |
| AL137026.1 | hsa-miR-204-5p | B4GALNT3 | 0.028938277 | 2.12E-45 |
| AL137026.1 | hsa-miR-138-5p | S100A1 | 0.022899818 | 3.00E-15 |
| AL137026.1 | hsa-miR-204-5p | RUNX2 | 0.010779147 | 5.38E-146 |
| AL137026.1 | hsa-miR-204-5p | PI4K2A | 0.001659884 | 4.97E-16 |
| AL137026.1 | hsa-miR-138-5p, hsa-miR-204-5p | MEX3A | 0.015640691 | 2.00E-46 |
| AL137026.1 | hsa-miR-204-5p | TTC39B | 0.046765827 | 6.90E-22 |
| AL137026.1 | hsa-miR-204-5p | IGFBP2 | 0.022899818 | 1.26E-09 |
| AL137026.1 | hsa-miR-204-5p | ALPL | 0.005771451 | 0.009743336 |
| AL137026.1 | hsa-miR-204-5p | MYRF | 0.011539106 | 1.23E-34 |
| AL137026.1 | hsa-miR-204-5p | ELOVL6 | 0.022393351 | 6.35E-19 |
| AL137026.1 | hsa-miR-204-5p | NOX5 | 0.011539106 | 0.00388988 |
| AL137026.1 | hsa-miR-150-5p | MMP14 | 0.001659884 | 6.55E-40 |
| AL137026.1 | hsa-miR-204-5p | PLAUR | 0.034165292 | 9.58E-69 |
| AL137026.1 | hsa-miR-204-5p | CREB5 | 0.001162861 | 6.17E-121 |
| AL137026.1 | hsa-miR-204-5p | STEAP4 | 0.005024776 | 1.20E-24 |
| AL137026.1 | hsa-miR-204-5p | ARNTL2 | 0.000167299 | 2.20E-22 |
| FOXD2-AS1 | hsa-miR-9-5p | CDH3 | 0.042119586 | 8.93E-36 |
| FOXD2-AS1 | hsa-miR-9-5p | P4HA2 | 0.02827522 | 3.08E-30 |
| FOXD2-AS1 | hsa-miR-150-5p | FAXC | 0.019664379 | 6.36E-38 |
| FOXD2-AS1 | hsa-miR-214-3p | ENTPD1 | 0.00022492 | 2.40E-16 |
| FOXD2-AS1 | hsa-miR-9-5p | CCND1 | 0.00194928 | 2.49E-18 |
| FOXD2-AS1 | hsa-miR-9-5p | KCNJ2 | 0.000557979 | 5.91E-26 |
| FOXD2-AS1 | hsa-miR-9-5p | NOX4 | 0.001920038 | 4.57E-31 |
| FOXD2-AS1 | hsa-miR-214-3p | JAG2 | 0.003396187 | 5.25E-26 |
| FOXD2-AS1 | hsa-miR-7-5p, hsa-miR-150-5p | XPR1 | 0.048183051 | 6.68E-24 |
| FOXD2-AS1 | hsa-miR-7-5p | TRIM47 | 0.014236245 | 2.70E-74 |
| FOXD2-AS1 | hsa-miR-9-5p, hsa-miR-221-3p | RUNX1 | 0.000290218 | 4.87E-49 |
| FOXD2-AS1 | hsa-miR-7-5p | RNF183 | 0.042119586 | 1.05E-19 |
| FOXD2-AS1 | hsa-miR-7-5p | FNDC4 | 0.02827522 | 1.93E-31 |
| FOXD2-AS1 | hsa-miR-150-5p | MAPK13 | 0.000483169 | 2.16E-23 |
| FOXD2-AS1 | hsa-miR-214-3p | LZTS1 | 0.000197269 | 1.79E-19 |
| FOXD2-AS1 | hsa-miR-150-5p, hsa-miR-214-3p | KCNK5 | 0.043997657 | 1.77E-12 |
| FOXD2-AS1 | hsa-miR-214-3p | TRIM29 | 0.000143262 | 6.48E-12 |
| FOXD2-AS1 | hsa-miR-150-5p | NOTCH3 | 0.000210492 | 5.43E-18 |
| FOXD2-AS1 | hsa-miR-9-5p, hsa-miR-222-3p | PRDM1 | 0.000355444 | 1.90E-12 |
| FOXD2-AS1 | hsa-miR-150-5p, hsa-miR-214-3p | TNFSF15 | 0.000369627 | 8.22E-13 |
| FOXD2-AS1 | hsa-miR-7-5p | SSX1 | 0.014236245 | 1.04E-18 |
| FOXD2-AS1 | hsa-miR-9-5p | PI4K2A | 0.010006118 | 3.97E-43 |
| FOXD2-AS1 | hsa-miR-7-5p | LAMC2 | 0.005232981 | 1.91E-20 |
| FOXD2-AS1 | hsa-miR-9-5p | TGFBI | 0.003396187 | 2.60E-27 |
| FOXD2-AS1 | hsa-miR-150-5p | PLXDC1 | 0.000143262 | 2.87E-32 |
| FOXD2-AS1 | hsa-miR-9-5p | RHOV | 0.000405402 | 6.33E-22 |
| FOXD2-AS1 | hsa-miR-214-3p | ASF1B | 0.006667978 | 1.73E-56 |
| FOXD2-AS1 | hsa-miR-7-5p | IGSF3 | 0.000355444 | 2.43E-14 |
| FOXD2-AS1 | hsa-miR-9-5p | SDC1 | 0.020877228 | 7.33E-11 |
| FOXD2-AS1 | hsa-miR-150-5p | MMP14 | 0.000405402 | 3.30E-15 |
| FOXD2-AS1 | hsa-miR-9-5p | NTRK3 | 0.000253939 | 0.004160001 |
| FOXD2-AS1 | hsa-miR-7-5p | ROR1 | 0.005232981 | 3.31E-11 |
| FOXD2-AS1 | hsa-miR-7-5p, hsa-miR-214-3p | PAPPA | 0.002200767 | 0.001082896 |
| LINC00607 | hsa-miR-150-5p, hsa-miR-204-5p | FAXC | 0.021956675 | 4.78E-102 |
| LINC00607 | hsa-miR-214-3p | ENTPD1 | 0.000485135 | 2.58E-17 |
| LINC00607 | hsa-miR-503-5p, hsa-miR-150-5p | ZMAT3 | 0.017417849 | 1.74E-49 |
| LINC00607 | hsa-miR-214-3p | JAG2 | 0.001037701 | 1.20E-11 |
| LINC00607 | hsa-miR-204-5p | SAMD1 | 0.004997252 | 1.64E-36 |
| LINC00607 | hsa-miR-138-5p | LCN2 | 0.037322047 | 3.97E-85 |
| LINC00607 | hsa-miR-150-5p, hsa-miR-375 | XPR1 | 0.001915295 | 3.55E-45 |
| LINC00607 | hsa-miR-214-3p | AHNAK2 | 0.000205773 | 3.50E-103 |
| LINC00607 | hsa-miR-204-5p | CDH4 | 0.006686705 | 1.83E-31 |
| LINC00607 | hsa-miR-146b-5p, hsa-miR-204-5p, hsa-miR-375 | IL1RAP | 0.000492042 | 5.29E-99 |
| LINC00607 | hsa-miR-138-5p | SEMA4C | 0.000804506 | 2.37E-65 |
| LINC00607 | hsa-miR-204-5p | SH2D4A | 0.023427933 | 4.54E-39 |
| LINC00607 | hsa-miR-138-5p | LPL | 0.004997252 | 2.58E-21 |
| LINC00607 | hsa-miR-204-5p, hsa-miR-34a-5p | CD44 | 0.000951133 | 8.10E-08 |
| LINC00607 | hsa-miR-138-5p, hsa-miR-204-5p, hsa-miR-363-3p, hsa-miR-31-5p | SOX4 | 0.000106915 | 1.96E-78 |
| LINC00607 | hsa-miR-363-3p | TOR4A | 0.004685515 | 2.70E-30 |
| LINC00607 | hsa-miR-181a-5p, hsa-miR-204-5p | TGFBR1 | 0.000476014 | 3.80E-65 |
| LINC00607 | hsa-miR-214-3p | LZTS1 | 0.001379106 | 7.08E-16 |
| LINC00607 | hsa-miR-363-3p | PMEPA1 | 0.00563866 | 5.19E-45 |
| LINC00607 | hsa-miR-214-3p | TRIM29 | 0.000648359 | 2.12E-53 |
| LINC00607 | hsa-miR-150-5p | NOTCH3 | 0.003594768 | 2.91E-10 |
| LINC00607 | hsa-miR-138-5p, hsa-miR-181a-5p | S100A1 | 0.000196314 | 9.83E-33 |
| LINC00607 | hsa-miR-150-5p, hsa-miR-214-3p | TNFSF15 | 0.044247843 | 8.19E-38 |
| LINC00607 | hsa-miR-204-5p | PI4K2A | 0.006686705 | 6.75E-47 |
| LINC00607 | hsa-miR-21-5p | TGFBI | 0.046405716 | 1.56E-20 |
| LINC00607 | hsa-miR-150-5p | PLXDC1 | 0.005575735 | 1.43E-13 |
| LINC00607 | hsa-miR-34a-5p, hsa-miR-375 | TGM2 | 0.025616204 | 9.58E-65 |
| LINC00607 | hsa-miR-204-5p | ALPL | 0.037322047 | 0.000221919 |
| LINC00607 | hsa-miR-214-3p | ASF1B | 0.000199064 | 1.59E-28 |
| LINC00607 | hsa-miR-204-5p | ELOVL6 | 0.016859512 | 4.28E-27 |
| LINC00607 | hsa-miR-375 | SDC1 | 0.019820376 | 4.59E-10 |
| LINC00607 | hsa-miR-204-5p | PLAUR | 0.018749283 | 2.59E-97 |
| LINC00607 | hsa-miR-503-5p, hsa-miR-96-5p, hsa-miR-146b-5p, hsa-miR-181a-5p, hsa-miR-182-5p, hsa-miR-363-3p | CDKN1A | 0.033394404 | 4.08E-19 |
| LINC00607 | hsa-miR-21-5p, hsa-miR-363-3p | BCAT1 | 0.002172329 | 4.33E-48 |
| LINC00607 | hsa-miR-204-5p | STEAP4 | 0.031638409 | 1.41E-22 |
| LINC00607 | hsa-miR-181a-5p | KLF6 | 0.001800459 | 2.21E-06 |
| LINC00607 | hsa-miR-204-5p | CXCL8 | 0.00181772 | 5.60E-66 |
| LINC00607 | hsa-miR-21-5p | CCR7 | 0.007876525 | 1.24E-11 |
| LINC00607 | hsa-miR-96-5p, hsa-miR-182-5p | RGS2 | 0.000380278 | 5.53E-08 |
| LINC00607 | hsa-miR-363-3p | FAM129A | 0.001149912 | 2.50E-46 |
| LINC00607 | hsa-miR-181a-5p, hsa-miR-181b-5p, hsa-miR-181d-5p | ARSJ | 0.01102734 | 1.27E-69 |
| LINC00607 | hsa-miR-214-3p | PAPPA | 0.000500954 | 2.02E-36 |
| LINC00607 | hsa-miR-146b-5p, hsa-miR-150-5p | CYTIP | 0.043050183 | 2.64E-16 |
| LINC00607 | hsa-miR-34a-5p | ZAP70 | 0.007876525 | 9.25E-11 |
| LBX2-AS1 | hsa-miR-214-3p | GALNT7 | 0.029865744 | 2.63E-31 |
| LBX2-AS1 | hsa-miR-503-5p, hsa-miR-96-5p | CCND1 | 0.000451272 | 5.19E-29 |
| LBX2-AS1 | hsa-miR-150-5p | CORO2A | 0.031522995 | 5.59E-29 |
| LBX2-AS1 | hsa-miR-146b-5p | IL1RAP | 0.003274016 | 3.32E-19 |
| LBX2-AS1 | hsa-miR-214-3p | LZTS1 | 0.01609786 | 5.68E-29 |
| LBX2-AS1 | hsa-miR-503-5p | PLAG1 | 0.003822866 | 3.02E-28 |
| LBX2-AS1 | hsa-miR-214-3p | BAX | 0.000194505 | 4.04E-48 |
| LBX2-AS1 | hsa-miR-150-5p, hsa-miR-214-3p | KCNK5 | 0.006215369 | 4.92E-15 |
| LBX2-AS1 | hsa-miR-150-5p, hsa-miR-214-3p | TNFSF15 | 0.002043167 | 6.90E-14 |
| LBX2-AS1 | hsa-miR-96-5p | EFHD2 | 0.027580365 | 7.59E-21 |
| LBX2-AS1 | hsa-miR-96-5p, hsa-miR-182-5p | CASTOR2 | 0.039339556 | 4.09E-06 |
| LBX2-AS1 | hsa-miR-503-5p, hsa-miR-96-5p, hsa-miR-146b-5p, hsa-miR-182-5p | CDKN1A | 0.012047048 | 4.35E-13 |
| LBX2-AS1 | hsa-miR-182-5p | CREB5 | 0.036708835 | 1.91E-24 |
| LBX2-AS1 | hsa-miR-214-3p | PAPPA | 0.009790336 | 2.81E-05 |
| LBX2-AS1 | hsa-miR-146b-5p, hsa-miR-150-5p | CYTIP | 0.002736013 | 8.11E-11 |
| MIR31HG | hsa-miR-214-3p | ENTPD1 | 0.005245796 | 4.24E-19 |
| MIR31HG | hsa-miR-214-3p, hsa-miR-34a-5p | GALNT7 | 0.00083643 | 1.59E-57 |
| MIR31HG | hsa-miR-214-3p | JAG2 | 0.000224435 | 2.80E-08 |
| MIR31HG | hsa-miR-7-5p | TRIM47 | 0.005771451 | 4.99E-32 |
| MIR31HG | hsa-miR-7-5p | UNC5CL | 0.045309446 | 1.14E-49 |
| MIR31HG | hsa-miR-7-5p | RNF183 | 0.017221204 | 1.20E-30 |
| MIR31HG | hsa-miR-7-5p | FNDC4 | 0.0115118 | 5.08E-42 |
| MIR31HG | hsa-miR-214-3p | LZTS1 | 0.0115118 | 2.15E-25 |
| MIR31HG | hsa-miR-214-3p | KCNK5 | 0.015640691 | 3.66E-24 |
| MIR31HG | hsa-miR-214-3p | TRIM29 | 0.000167299 | 1.31E-42 |
| MIR31HG | hsa-miR-214-3p | TNFSF15 | 0.00674945 | 6.17E-25 |
| MIR31HG | hsa-miR-7-5p | SSX1 | 0.005771451 | 1.39E-22 |
| MIR31HG | hsa-miR-34a-5p | HRK | 0.005721473 | 2.25E-05 |
| MIR31HG | hsa-miR-7-5p | LAMC2 | 0.045309446 | 3.49E-20 |
| MIR31HG | hsa-miR-7-5p, hsa-miR-34a-5p | TGM2 | 0.000642298 | 3.09E-31 |
| MIR31HG | hsa-miR-7-5p, hsa-miR-34a-5p | EFHD2 | 0.001009328 | 5.02E-23 |
| MIR31HG | hsa-miR-214-3p | ASF1B | 0.00109375 | 8.66E-11 |
| MIR31HG | hsa-miR-7-5p | DSC2 | 0.039752457 | 4.82E-38 |
| MIR31HG | hsa-miR-7-5p | ROR1 | 0.000853541 | 7.43E-42 |
| MIR31HG | hsa-miR-7-5p, hsa-miR-214-3p | PAPPA | 0.005024776 | 1.13E-30 |
| MIR31HG | hsa-miR-34a-5p | PDGFRA | 0.046765827 | 3.48E-13 |
| MIR31HG | hsa-miR-34a-5p | ZAP70 | 0.000185362 | 4.70E-09 |
| AC125807.2 | hsa-miR-181a-5p, hsa-miR-181b-5p, hsa-miR-181d-5p | ZFP36L2 | 0.001684763 | 1.77E-41 |
| AC125807.2 | hsa-miR-9-5p, hsa-miR-181a-5p, hsa-miR-181b-5p, hsa-miR-181d-5p | ID4 | 0.004421583 | 7.66E-55 |
| AC125807.2 | hsa-miR-181a-5p, hsa-miR-181b-5p, hsa-miR-181d-5p | WDR72 | 0.00041741 | 7.58E-49 |
| AC125807.2 | hsa-miR-181a-5p, hsa-miR-181b-5p | TNFRSF11B | 0.004874611 | 1.33E-57 |
| AC125807.2 | hsa-miR-181a-5p, hsa-miR-181b-5p, hsa-miR-181d-5p | RGS16 | 0.000515309 | 2.32E-39 |
| AC125807.2 | hsa-miR-181a-5p | MOB3B | 0.002292454 | 1.53E-43 |
| AC125807.2 | hsa-miR-181a-5p, hsa-miR-181b-5p, hsa-miR-181d-5p | ZNF781 | 0.000913779 | 1.48E-25 |
| AC125807.2 | hsa-miR-9-5p, hsa-miR-205-5p | SLC39A14 | 0.024814762 | 1.03E-62 |
| AC125807.2 | hsa-miR-146b-5p, hsa-miR-199b-5p | KIT | 0.005357438 | 1.36E-93 |
| AC125807.2 | hsa-miR-205-5p | CTGF | 0.010393003 | 4.12E-25 |
| AC125807.2 | hsa-miR-181a-5p | AKAP12 | 0.021882414 | 1.00E-45 |
| AC125807.2 | hsa-miR-7-5p, hsa-miR-181a-5p, hsa-miR-181b-5p | FOS | 0.025652376 | 2.67E-27 |
| AC125807.2 | hsa-miR-31-5p | CXCL12 | 0.019152023 | 2.47E-15 |
| AP001258.1 | hsa-miR-221-3p | RUNX1 | 0.004882863 | 1.47E-89 |
| AP001258.1 | hsa-miR-363-3p | PLXNA3 | 0.00028388 | 1.65E-77 |
| AP001258.1 | hsa-miR-363-3p | SOX4 | 0.000353659 | 3.04E-78 |
| AP001258.1 | hsa-miR-363-3p | PMEPA1 | 0.000382056 | 8.35E-42 |
| AP001258.1 | hsa-miR-222-3p | RUNX2 | 0.0010888 | 3.23E-99 |
| AP001258.1 | hsa-miR-451a | DCBLD2 | 0.021714738 | 3.14E-58 |
| AP001258.1 | hsa-miR-182-5p, hsa-miR-363-3p | CDKN1A | 0.000193258 | 4.29E-07 |
| AP001258.1 | hsa-miR-182-5p | CREB5 | 0.003470048 | 8.99E-78 |
| AP001258.1 | hsa-miR-221-3p, hsa-miR-222-3p | ESR1 | 0.000157646 | 9.20E-18 |
| DCST1-AS1 | hsa-miR-138-5p | CCND1 | 0.014274236 | 4.08E-16 |
| DCST1-AS1 | hsa-miR-138-5p | LCN2 | 0.00230858 | 2.09E-30 |
| DCST1-AS1 | hsa-miR-146b-5p | IL1RAP | 0.000242114 | 3.20E-19 |
| DCST1-AS1 | hsa-miR-138-5p | LPL | 0.022886687 | 1.81E-08 |
| DCST1-AS1 | hsa-miR-138-5p | S100A1 | 0.00920769 | 3.70E-39 |
| DCST1-AS1 | hsa-miR-138-5p | YPEL4 | 0.029666941 | 8.20E-23 |
| DCST1-AS1 | hsa-miR-146b-5p | PMAIP1 | 0.00013494 | 6.85E-10 |
| LINC01137 | hsa-miR-214-3p | ENTPD1 | 0.004271145 | 1.01E-15 |
| LINC01137 | hsa-miR-214-3p | JAG2 | 0.000180538 | 1.82E-24 |
| LINC01137 | hsa-miR-146b-5p | IL1RAP | 0.001441897 | 1.69E-14 |
| LINC01137 | hsa-miR-214-3p | KCNK5 | 0.012848101 | 9.68E-14 |
| LINC01137 | hsa-miR-214-3p | TRIM29 | 0.0001345 | 1.10E-05 |
| LINC01137 | hsa-miR-214-3p | RAB15 | 0.012122772 | 1.44E-06 |
| LINC01137 | hsa-miR-214-3p | TNFSF15 | 0.005504888 | 1.55E-08 |
| LINC01137 | hsa-miR-214-3p | ASF1B | 0.04752023 | 1.82E-19 |
| LINC01137 | hsa-miR-146b-5p | CDKN1A | 0.00078746 | 1.57E-11 |
| LINC01137 | hsa-miR-146b-5p | CYTIP | 0.015292849 | 0.007264328 |
| AC092803.2 | hsa-miR-204-5p | TPPP | 0.004218821 | 5.49E-34 |
| AC092803.2 | hsa-miR-182-5p | TCEAL7 | 0.031307713 | 1.47E-10 |
| AC092803.2 | hsa-miR-182-5p | RGS2 | 0.031673544 | 8.82E-08 |
| AC092803.2 | hsa-miR-204-5p | SAMD5 | 0.040663693 | 5.16E-13 |
| AC092803.2 | hsa-miR-20b-5p | ESR1 | 0.004889526 | 0.045515143 |
| OR2A1-AS1 | hsa-miR-34a-5p | CYTH3 | 0.000244743 | 2.53E-40 |
| OR2A1-AS1 | hsa-miR-96-5p | SOX5 | 0.000816261 | 1.71E-24 |
| OR2A1-AS1 | hsa-miR-503-5p | KIAA1456 | 0.022648593 | 1.02E-44 |
| OR2A1-AS1 | hsa-miR-96-5p, hsa-miR-34a-5p | CDON | 0.004175073 | 8.81E-45 |
| OR2A1-AS1 | hsa-miR-9-5p, hsa-miR-205-5p | SLC39A14 | 0.000450874 | 3.67E-40 |
| OR2A1-AS1 | hsa-miR-199b-5p, hsa-miR-34a-5p | KIT | 0.000845129 | 4.81E-49 |
| OR2A1-AS1 | hsa-miR-205-5p | CTGF | 0.000248251 | 5.31E-12 |
| OR2A1-AS1 | hsa-miR-7-5p, hsa-miR-34a-5p | FOS | 0.001831469 | 2.92E-14 |
| OR2A1-AS1 | hsa-miR-33b-5p, hsa-miR-34a-5p | GAS1 | 0.00059904 | 2.34E-05 |
| LINC01135 | hsa-miR-34a-5p | CYTH3 | 0.000123268 | 1.60E-61 |
| LINC01135 | hsa-miR-96-5p, hsa-miR-205-5p | SLC25A25 | 0.001866371 | 6.81E-29 |
| LINC01135 | hsa-miR-96-5p | SOX5 | 0.014780673 | 1.58E-10 |
| LINC01135 | hsa-miR-31-5p | WASF3 | 0.022722335 | 4.74E-105 |
| LINC01135 | hsa-miR-205-5p | SLC39A14 | 0.002197773 | 2.97E-63 |
| LINC01135 | hsa-miR-34a-5p | KIT | 0.026107384 | 5.09E-61 |
| LINC01135 | hsa-miR-205-5p | CTGF | 0.042019066 | 9.15E-08 |
| LINC01135 | hsa-miR-20b-5p | KLF6 | 0.000158849 | 0.018041406 |
| LINC01135 | hsa-miR-150-5p | PTPRR | 0.026163909 | 0.000244636 |
| PPP1R12A-AS1 | hsa-miR-181a-5p, hsa-miR-181b-5p, hsa-miR-181d-5p | ZFP36L2 | 0.000378862 | 7.36E-36 |
| PPP1R12A-AS1 | hsa-miR-181a-5p | SOX5 | 0.002961969 | 3.43E-21 |
| PPP1R12A-AS1 | hsa-miR-181a-5p, hsa-miR-181b-5p, hsa-miR-181d-5p | ID4 | 0.000615086 | 1.04E-79 |
| PPP1R12A-AS1 | hsa-miR-181a-5p, hsa-miR-181b-5p | TNFRSF11B | 0.00036958 | 5.74E-56 |
| PPP1R12A-AS1 | hsa-miR-221-3p | TUB | 0.007862583 | 1.42E-61 |
| PPP1R12A-AS1 | hsa-miR-221-3p | SPAG5 | 0.03978267 | 1.54E-38 |
| PPP1R12A-AS1 | hsa-miR-181a-5p | ALDH1A1 | 0.01609786 | 1.79E-52 |
| PPP1R12A-AS1 | hsa-miR-221-3p, hsa-miR-222-3p | KIT | 0.0009333 | 2.19E-81 |
| PPP1R12A-AS1 | hsa-miR-181a-5p, hsa-miR-181b-5p, hsa-miR-221-3p, hsa-miR-222-3p | FOS | 0.001007295 | 3.65E-10 |
| PPP1R12A-AS1 | hsa-miR-181a-5p | RGS5 | 0.006598284 | 0.007006026 |
| PPP1R12A-AS1 | hsa-miR-181a-5p | GPR83 | 0.024053942 | 1.81E-33 |
| PPP1R12A-AS1 | hsa-miR-221-3p | CXCL12 | 0.010825396 | 0.01739452 |
| PPP1R12A-AS1 | hsa-miR-181a-5p, hsa-miR-221-3p | TMEM132B | 0.040338245 | 5.56E-30 |
| AC007743.1 | hsa-miR-96-5p, hsa-miR-21-5p | SOX5 | 0.018276099 | 1.68E-36 |
| AC007743.1 | hsa-miR-21-5p | TNFRSF11B | 0.001359314 | 2.76E-55 |
| AC007743.1 | hsa-miR-21-5p | LIFR | 0.001321346 | 6.78E-48 |
| AC007743.1 | hsa-miR-182-5p, hsa-miR-31-5p | SYDE2 | 0.047672419 | 3.19E-47 |
| AC007743.1 | hsa-miR-21-5p | FMOD | 0.000231036 | 7.80E-35 |
| AC007743.1 | hsa-miR-96-5p | CDON | 0.043947279 | 5.89E-36 |
| AC007743.1 | hsa-miR-21-5p | FAXDC2 | 0.000231036 | 5.51E-28 |
| AC007743.1 | hsa-miR-182-5p | TCEAL7 | 0.030550034 | 4.26E-08 |
| AC007743.1 | hsa-miR-363-3p | MRO | 0.001241701 | 3.19E-33 |
| AC007743.1 | hsa-miR-96-5p, hsa-miR-182-5p | RGS2 | 0.000126131 | 6.57E-05 |
| AC007743.1 | hsa-miR-21-5p | SOD3 | 0.015390535 | 8.44E-21 |
| AC007743.1 | hsa-miR-204-5p, hsa-miR-21-5p | SAMD5 | 0.006190024 | 3.09E-18 |
| AC072061.1 | hsa-miR-21-5p, hsa-miR-375 | TNS3 | 0.023062646 | 6.07E-13 |
| AC072061.1 | hsa-miR-96-5p, hsa-miR-205-5p | SLC25A25 | 0.000136569 | 0.01249953 |
| AC072061.1 | hsa-miR-96-5p, hsa-miR-21-5p | SOX5 | 0.017070689 | 7.88E-16 |
| AC072061.1 | hsa-miR-9-5p | ID4 | 0.045437551 | 8.89E-47 |
| AC072061.1 | hsa-miR-21-5p | TNFRSF11B | 0.001292018 | 6.29E-21 |
| AC072061.1 | hsa-miR-21-5p | LIFR | 0.0479231 | 3.57E-34 |
| AC072061.1 | hsa-miR-182-5p, hsa-miR-31-5p | SYDE2 | 0.045530463 | 1.62E-08 |
| AC072061.1 | hsa-miR-31-5p | WASF3 | 0.023956831 | 6.59E-27 |
| AC072061.1 | hsa-miR-21-5p | FMOD | 0.000219484 | 1.31E-20 |
| AC072061.1 | hsa-miR-34a-5p | RCAN1 | 0.032145345 | 4.21E-07 |
| AC072061.1 | hsa-miR-204-5p, hsa-miR-205-5p | NOX5 | 0.008718862 | 0.000487927 |
| AC072061.1 | hsa-miR-96-5p, hsa-miR-34a-5p | CDON | 0.003950941 | 3.92E-60 |
| AC072061.1 | hsa-miR-21-5p | FAXDC2 | 0.000219484 | 4.95E-49 |
| AC072061.1 | hsa-miR-204-5p | FAM167B | 0.035167782 | 1.58E-06 |
| AC072061.1 | hsa-miR-182-5p | TCEAL7 | 0.029792059 | 8.45E-21 |
| AC072061.1 | hsa-miR-363-3p | MRO | 0.001061293 | 2.57E-60 |
| AC072061.1 | hsa-miR-375 | NCAM1 | 0.004394775 | 1.37E-11 |
| AC072061.1 | hsa-miR-21-5p | SOD3 | 0.015005771 | 2.10E-37 |
| AC072061.1 | hsa-miR-204-5p, hsa-miR-21-5p | SAMD5 | 0.000687891 | 3.70E-30 |
| AC072061.1 | hsa-miR-34a-5p | GAS1 | 0.00162332 | 0.003022232 |
| AL365259.1 | hsa-miR-214-3p | JAG2 | 0.00033175 | 7.90E-22 |
| AL365259.1 | hsa-miR-375 | XPR1 | 0.011060967 | 1.75E-17 |
| AL365259.1 | hsa-miR-214-3p | AHNAK2 | 0.000123607 | 6.53E-16 |
| AL365259.1 | hsa-miR-214-3p | DOCK9 | 0.003011658 | 6.97E-20 |
| AL365259.1 | hsa-miR-214-3p | BAX | 0.004023064 | 1.37E-17 |
| AL365259.1 | hsa-miR-214-3p | KCNK5 | 0.022173118 | 5.40E-44 |
| AL365259.1 | hsa-miR-214-3p | TNFSF15 | 0.000881502 | 1.01E-14 |
| AL365259.1 | hsa-miR-375 | TGM2 | 0.00082979 | 6.08E-05 |
| AL365259.1 | hsa-miR-375 | SDC1 | 0.004580129 | 0.035535756 |
| AL365259.1 | hsa-miR-375 | ARNTL2 | 0.007141163 | 0.016062957 |
| AL365259.1 | hsa-miR-214-3p | PAPPA | 0.000211299 | 0.016018271 |
| AL645608.1 | hsa-miR-33b-5p | HMGA2 | 0.043724843 | 1.79E-06 |
| AL645608.1 | hsa-miR-150-5p | TMEM92 | 0.0038464 | 6.84E-10 |
| AL645608.1 | hsa-miR-138-5p | MTHFD1L | 0.042940134 | 0.000912782 |
| AL645608.1 | hsa-miR-138-5p | SEMA4C | 0.002520957 | 1.55E-08 |
| AL645608.1 | hsa-miR-138-5p | LPL | 0.004776035 | 0.000315971 |
| AL645608.1 | hsa-miR-138-5p | YPEL4 | 0.008114628 | 1.27E-20 |
| AL645608.1 | hsa-miR-150-5p | C3orf36 | 0.032583598 | 0.000624868 |
| AL645608.1 | hsa-miR-150-5p | PTPRR | 0.010773374 | 0.015779665 |
| LIFR-AS1 | hsa-miR-221-3p | TLE4 | 0.005773788 | 0.001321051 |
| LIFR-AS1 | hsa-miR-96-5p, hsa-miR-181a-5p | SOX5 | 0.022424302 | 1.43E-13 |
| LIFR-AS1 | hsa-miR-144-3p, hsa-miR-375 | ELL2 | 0.00014232 | 3.76E-11 |
| LIFR-AS1 | hsa-miR-181a-5p, hsa-miR-181b-5p, hsa-miR-181d-5p | WDR72 | 0.000443787 | 1.98E-75 |
| LIFR-AS1 | hsa-miR-144-3p, hsa-miR-181a-5p | TGFBR3 | 0.01318851 | 2.25E-28 |
| LIFR-AS1 | hsa-miR-221-3p | TUB | 0.012606218 | 3.62E-52 |
| LIFR-AS1 | hsa-miR-375 | SLC7A6 | 0.008176839 | 2.02E-20 |
| LIFR-AS1 | hsa-miR-96-5p, hsa-miR-221-3p | PRDM16 | 0.0008036 | 5.07E-29 |
| LIFR-AS1 | hsa-miR-181a-5p, hsa-miR-181b-5p, hsa-miR-181d-5p | RGS16 | 0.000547697 | 8.72E-51 |
| LIFR-AS1 | hsa-miR-146b-5p | RARB | 0.007447621 | 7.88E-60 |
| LIFR-AS1 | hsa-miR-96-5p | CDON | 0.025679976 | 3.96E-62 |
| LIFR-AS1 | hsa-miR-181a-5p, hsa-miR-181b-5p, hsa-miR-181d-5p | ZNF781 | 0.000970275 | 6.60E-46 |
| LIFR-AS1 | hsa-miR-181a-5p | ALDH1A1 | 0.00086668 | 3.23E-28 |
| LIFR-AS1 | hsa-miR-181a-5p | OCA2 | 0.047186437 | 3.26E-25 |
| LIFR-AS1 | hsa-miR-375 | CTGF | 0.001597656 | 8.72E-05 |
| LIFR-AS1 | hsa-miR-181a-5p | GPR83 | 0.002549982 | 1.20E-24 |
| LIFR-AS1 | hsa-miR-150-5p | PTPRR | 0.02962678 | 2.08E-05 |
| LIFR-AS1 | hsa-miR-375 | NCAM1 | 0.016521484 | 1.25E-31 |
| LIFR-AS1 | hsa-miR-221-3p, hsa-miR-31-5p | CXCL12 | 0.002406054 | 0.001375806 |
| AC092118.1 | hsa-miR-204-5p, hsa-miR-34a-5p | HMGA2 | 0.045717722 | 4.04E-30 |
| AC092118.1 | hsa-miR-9-5p | CDH3 | 0.046600891 | 3.37E-42 |
| AC092118.1 | hsa-miR-9-5p | P4HA2 | 0.031307713 | 1.48E-17 |
| AC092118.1 | hsa-miR-214-3p | ENTPD1 | 0.000402712 | 9.65E-21 |
| AC092118.1 | hsa-miR-214-3p, hsa-miR-34a-5p | GALNT7 | 0.001756636 | 7.66E-26 |
| AC092118.1 | hsa-miR-9-5p, hsa-miR-138-5p, hsa-miR-34a-5p | CCND1 | 0.004432596 | 2.79E-20 |
| AC092118.1 | hsa-miR-204-5p | BID | 0.038562503 | 1.08E-38 |
| AC092118.1 | hsa-miR-9-5p | NOX4 | 0.002356686 | 1.29E-22 |
| AC092118.1 | hsa-miR-214-3p | JAG2 | 0.000317785 | 5.63E-17 |
| AC092118.1 | hsa-miR-138-5p | MTHFD1L | 0.012059109 | 1.94E-07 |
| AC092118.1 | hsa-miR-204-5p | SAMD1 | 0.010087256 | 2.60E-18 |
| AC092118.1 | hsa-miR-138-5p | LCN2 | 0.015775298 | 9.47E-11 |
| AC092118.1 | hsa-miR-7-5p | TRIM47 | 0.015775298 | 6.61E-21 |
| AC092118.1 | hsa-miR-204-5p | CDH4 | 0.012206416 | 6.70E-08 |
| AC092118.1 | hsa-miR-146b-5p, hsa-miR-204-5p | IL1RAP | 0.000551036 | 1.96E-32 |
| AC092118.1 | hsa-miR-138-5p | SEMA4C | 0.01017223 | 7.82E-45 |
| AC092118.1 | hsa-miR-204-5p | SH2D4A | 0.028434643 | 1.15E-27 |
| AC092118.1 | hsa-miR-7-5p | RNF183 | 0.046600891 | 1.18E-32 |
| AC092118.1 | hsa-miR-138-5p | LPL | 0.010087256 | 1.21E-08 |
| AC092118.1 | hsa-miR-7-5p | FNDC4 | 0.000242884 | 2.82E-22 |
| AC092118.1 | hsa-miR-204-5p, hsa-miR-34a-5p | CD44 | 0.004328813 | 7.46E-18 |
| AC092118.1 | hsa-miR-9-5p | RAB34 | 0.000151183 | 2.36E-38 |
| AC092118.1 | hsa-miR-204-5p | TGFBR1 | 0.044982394 | 1.94E-36 |
| AC092118.1 | hsa-miR-214-3p | LZTS1 | 0.000242884 | 1.92E-15 |
| AC092118.1 | hsa-miR-7-5p | GLDN | 0.025339516 | 1.22E-25 |
| AC092118.1 | hsa-miR-204-5p | PLAG1 | 0.039934808 | 1.61E-32 |
| AC092118.1 | hsa-miR-204-5p | B4GALNT3 | 0.005826145 | 1.48E-16 |
| AC092118.1 | hsa-miR-9-5p | PRDM1 | 0.006587923 | 1.31E-19 |
| AC092118.1 | hsa-miR-214-3p | TNFSF15 | 0.023073454 | 1.20E-12 |
| AC092118.1 | hsa-miR-7-5p | SSX1 | 0.015775298 | 3.50E-23 |
| AC092118.1 | hsa-miR-9-5p, hsa-miR-204-5p | PI4K2A | 0.000551036 | 2.70E-08 |
| AC092118.1 | hsa-miR-138-5p | YPEL4 | 0.016969276 | 3.57E-31 |
| AC092118.1 | hsa-miR-9-5p | ELF3 | 0.035051148 | 8.09E-36 |
| AC092118.1 | hsa-miR-7-5p | LAMC2 | 0.000193299 | 1.50E-11 |
| AC092118.1 | hsa-miR-9-5p, hsa-miR-21-5p | TGFBI | 0.045967233 | 3.77E-09 |
| AC092118.1 | hsa-miR-9-5p | FSTL3 | 0.002942861 | 2.73E-30 |
| AC092118.1 | hsa-miR-9-5p | MKX | 0.012059109 | 0.001147667 |
| AC092118.1 | hsa-miR-7-5p, hsa-miR-34a-5p | TGM2 | 0.004850778 | 2.24E-09 |
| AC092118.1 | hsa-miR-204-5p | ALPL | 0.015775298 | 0.001142928 |
| AC092118.1 | hsa-miR-7-5p, hsa-miR-34a-5p | EFHD2 | 0.039934808 | 1.45E-05 |
| AC092118.1 | hsa-miR-204-5p | LRRC4 | 0.00212606 | 0.048561029 |
| AC092118.1 | hsa-miR-7-5p | IGSF3 | 0.006587923 | 0.003106182 |
| AC092118.1 | hsa-miR-204-5p | ELOVL6 | 0.02565363 | 5.87E-06 |
| AC092118.1 | hsa-miR-9-5p | TM4SF1 | 0.023073454 | 7.11E-17 |
| AC092118.1 | hsa-miR-204-5p | FAM167B | 0.038562503 | 0.002996585 |
| AC092118.1 | hsa-miR-9-5p | NTRK3 | 0.005184856 | 1.07E-07 |
| AC092118.1 | hsa-miR-204-5p | PLAUR | 0.003499742 | 1.07E-17 |
| AC092118.1 | hsa-miR-7-5p | DSC2 | 0.004850778 | 4.26E-16 |
| AC092118.1 | hsa-miR-146b-5p | CDKN1A | 0.001134962 | 0.006513915 |
| AC092118.1 | hsa-miR-204-5p | CREB5 | 0.000260326 | 1.10E-27 |
| AC092118.1 | hsa-miR-204-5p | STEAP4 | 0.00296391 | 8.35E-08 |
| AC092118.1 | hsa-miR-204-5p | CXCL8 | 0.018140748 | 1.93E-13 |
| AC092118.1 | hsa-miR-146b-5p | PMAIP1 | 0.025721801 | 0.000188491 |
| AC092118.1 | hsa-miR-7-5p | ROR1 | 0.006403271 | 1.01E-10 |
| AC092118.1 | hsa-miR-204-5p | ARNTL2 | 0.038562503 | 0.000222386 |
| AC092118.1 | hsa-miR-7-5p, hsa-miR-214-3p | PAPPA | 0.00296391 | 5.55E-13 |
| MIR22HG | hsa-miR-375 | SLC7A6 | 0.034178302 | 1.09E-45 |
| MIR22HG | hsa-miR-375 | SLC25A15 | 0.018709596 | 9.18E-46 |
| MIR22HG | hsa-miR-375 | NCAM1 | 0.047538211 | 5.03E-26 |
| AF127577.4 | hsa-miR-199b-5p | PLXND1 | 0.004085357 | 1.60E-25 |
| AF127577.4 | hsa-miR-183-5p | CCND1 | 0.000398693 | 2.16E-24 |
| AF127577.4 | hsa-miR-205-5p, hsa-miR-375 | XPR1 | 0.000583116 | 6.25E-57 |
| AF127577.4 | hsa-miR-221-3p | RUNX1 | 0.021635424 | 1.02E-62 |
| AF127577.4 | hsa-miR-21-5p | TGFB1 | 0.005551547 | 1.52E-27 |
| AF127577.4 | hsa-miR-375 | PLAG1 | 0.043097492 | 1.02E-46 |
| AF127577.4 | hsa-miR-222-3p | PRDM1 | 0.007049831 | 9.98E-32 |
| AF127577.4 | hsa-miR-199b-5p | TSC22D1 | 0.023434989 | 6.67E-79 |
| AF127577.4 | hsa-miR-375 | FSTL3 | 0.023956831 | 1.42E-36 |
| AF127577.4 | hsa-miR-375 | TGM2 | 0.005086748 | 7.31E-24 |
| AF127577.4 | hsa-miR-183-5p | EFHD2 | 0.009889311 | 6.11E-10 |
| AF127577.4 | hsa-miR-21-5p | BCAT1 | 0.045477663 | 2.86E-14 |
| AF127577.4 | hsa-miR-205-5p | SLC7A2 | 0.005433992 | 3.87E-30 |
| AF127577.4 | hsa-miR-375 | ARNTL2 | 0.040305827 | 1.49E-19 |
| AF127577.4 | hsa-miR-221-3p, hsa-miR-222-3p | ESR1 | 0.005432387 | 9.79E-26 |
| AC009041.2 | hsa-miR-9-5p | P4HA2 | 0.045647331 | 7.12E-18 |
| AC009041.2 | hsa-miR-150-5p | TMEM92 | 0.01700119 | 5.49E-32 |
| AC009041.2 | hsa-miR-214-3p | JAG2 | 0.013184566 | 1.81E-18 |
| AC009041.2 | hsa-miR-138-5p | MTHFD1L | 0.033242024 | 3.55E-32 |
| AC009041.2 | hsa-miR-138-5p | LCN2 | 0.023085802 | 2.89E-44 |
| AC009041.2 | hsa-miR-9-5p | RUNX1 | 0.003922996 | 8.49E-35 |
| AC009041.2 | hsa-miR-138-5p | SEMA4C | 0.001412981 | 3.53E-23 |
| AC009041.2 | hsa-miR-138-5p | LPL | 0.020938678 | 0.000183613 |
| AC009041.2 | hsa-miR-214-3p | DOCK9 | 0.03471975 | 3.13E-13 |
| AC009041.2 | hsa-miR-138-5p, hsa-miR-20b-5p | SOX4 | 0.013436541 | 2.29E-21 |
| AC009041.2 | hsa-miR-214-3p | BAX | 0.004369047 | 7.24E-30 |
| AC009041.2 | hsa-miR-9-5p | PRDM1 | 0.018750631 | 4.75E-08 |
| AC009041.2 | hsa-miR-214-3p | RAB15 | 0.000213303 | 2.43E-23 |
| AC009041.2 | hsa-miR-150-5p, hsa-miR-214-3p | TNFSF15 | 0.021267369 | 1.77E-06 |
| AC009041.2 | hsa-miR-150-5p | PLXDC1 | 0.000943542 | 1.25E-07 |
| AC009041.2 | hsa-miR-20b-5p | DCBLD2 | 0.000543351 | 2.73E-10 |
| AC009041.2 | hsa-miR-96-5p, hsa-miR-183-5p | EFHD2 | 0.040656266 | 0.000483731 |
| AC009041.2 | hsa-miR-214-3p | ASF1B | 0.01700119 | 4.53E-32 |
| AC009041.2 | hsa-miR-9-5p | SDC1 | 0.00037985 | 2.12E-10 |
| AC009041.2 | hsa-miR-182-5p | CREB5 | 0.010544424 | 7.71E-16 |
| AC009041.2 | hsa-miR-9-5p | SLC7A2 | 0.018882626 | 6.66E-05 |
| AC009041.2 | hsa-miR-214-3p | PAPPA | 0.000768005 | 5.12E-15 |
| AC009041.2 | hsa-miR-20b-5p | RUNX3 | 0.000598024 | 0.033013575 |
| PAX8-AS1 | hsa-miR-144-3p, hsa-miR-181a-5p, hsa-miR-181b-5p, hsa-miR-181d-5p | ZFP36L2 | 0.000747432 | 8.77E-28 |
| PAX8-AS1 | hsa-miR-96-5p, hsa-miR-181a-5p | SOX5 | 0.000181727 | 1.99E-32 |
| PAX8-AS1 | hsa-miR-144-3p | ELL2 | 0.000111784 | 1.20E-13 |
| PAX8-AS1 | hsa-miR-181a-5p, hsa-miR-181b-5p, hsa-miR-181d-5p | ID4 | 0.000655474 | 2.53E-41 |
| PAX8-AS1 | hsa-miR-181a-5p, hsa-miR-181b-5p, hsa-miR-181d-5p | WDR72 | 0.003088066 | 4.35E-30 |
| PAX8-AS1 | hsa-miR-181a-5p, hsa-miR-181b-5p | TNFRSF11B | 0.011549504 | 1.66E-40 |
| PAX8-AS1 | hsa-miR-181a-5p, hsa-miR-183-5p | EGR1 | 0.047832682 | 7.65E-17 |
| PAX8-AS1 | hsa-miR-96-5p | PRDM16 | 0.002793565 | 3.95E-34 |
| PAX8-AS1 | hsa-miR-181a-5p, hsa-miR-181b-5p, hsa-miR-181d-5p | RGS16 | 0.00053001 | 3.26E-28 |
| PAX8-AS1 | hsa-miR-181a-5p | MOB3B | 0.011119741 | 5.91E-23 |
| PAX8-AS1 | hsa-miR-181a-5p, hsa-miR-181b-5p, hsa-miR-181d-5p | ZNF781 | 0.006406022 | 1.00E-22 |
| PAX8-AS1 | hsa-miR-363-3p | MRO | 0.001229416 | 1.33E-42 |
| PAX8-AS1 | hsa-miR-181a-5p, hsa-miR-183-5p | AKAP12 | 0.0132635 | 5.38E-26 |
| PAX8-AS1 | hsa-miR-7-5p, hsa-miR-181a-5p, hsa-miR-181b-5p | FOS | 0.01460246 | 5.30E-19 |
| PAX8-AS1 | hsa-miR-181a-5p | GPR83 | 0.005951341 | 1.94E-12 |
| PAX8-AS1 | hsa-miR-183-5p | GAS1 | 0.0042433 | 1.76E-12 |
| PAX8-AS1 | hsa-miR-181a-5p | PDGFRA | 0.005554741 | 0.001231005 |
| LINC00472 | hsa-miR-503-5p | KIAA1456 | 0.002250142 | 1.24E-74 |
| LINC00472 | hsa-miR-204-5p | SAMD5 | 0.02117101 | 9.55E-21 |
| AC008555.1 | hsa-miR-199b-5p | PLXND1 | 0.04693174 | 1.47E-28 |
| AC008555.1 | hsa-miR-34a-5p | CCND1 | 0.000103895 | 6.81E-36 |
| AC008555.1 | hsa-miR-7-5p | TRIM47 | 0.014236245 | 3.88E-09 |
| AC008555.1 | hsa-miR-146b-5p, hsa-miR-375 | IL1RAP | 0.000405402 | 1.28E-41 |
| AC008555.1 | hsa-miR-7-5p | RNF183 | 0.042119586 | 3.39E-15 |
| AC008555.1 | hsa-miR-7-5p | FNDC4 | 0.02827522 | 1.49E-14 |
| AC008555.1 | hsa-miR-199b-5p | C1orf226 | 0.041360308 | 1.19E-24 |
| AC008555.1 | hsa-miR-363-3p | PLXNA3 | 0.000713771 | 7.86E-19 |
| AC008555.1 | hsa-miR-363-3p | SOX4 | 0.008202441 | 2.82E-32 |
| AC008555.1 | hsa-miR-363-3p | TOR4A | 0.000431411 | 4.53E-26 |
| AC008555.1 | hsa-miR-375 | PLAG1 | 0.028660174 | 1.71E-46 |
| AC008555.1 | hsa-miR-7-5p | SSX1 | 0.014236245 | 3.75E-17 |
| AC008555.1 | hsa-miR-146b-5p, hsa-miR-363-3p | CDKN1A | 0.038009678 | 2.49E-05 |
| AC008555.1 | hsa-miR-7-5p | RGS5 | 0.004662749 | 1.45E-05 |
| LINC00900 | hsa-miR-150-5p | TMEM92 | 0.037191841 | 1.06E-10 |
| LINC00900 | hsa-miR-204-5p | BID | 0.030749313 | 1.54E-12 |
| LINC00900 | hsa-miR-214-3p | JAG2 | 0.006341936 | 4.14E-14 |
| LINC00900 | hsa-miR-204-5p | SAMD1 | 0.004168642 | 1.43E-07 |
| LINC00900 | hsa-miR-7-5p | TRIM47 | 0.035013467 | 2.58E-07 |
| LINC00900 | hsa-miR-204-5p | CDH4 | 0.005587657 | 9.74E-06 |
| LINC00900 | hsa-miR-204-5p | SH2D4A | 0.01977955 | 6.45E-09 |
| LINC00900 | hsa-miR-204-5p, hsa-miR-34a-5p | CD44 | 0.002629657 | 1.65E-31 |
| LINC00900 | hsa-miR-363-3p | PLXNA3 | 0.01903854 | 8.19E-12 |
| LINC00900 | hsa-miR-150-5p | QSOX1 | 0.012636752 | 2.28E-13 |
| LINC00900 | hsa-miR-150-5p | MAPK13 | 0.013314975 | 1.76E-10 |
| LINC00900 | hsa-miR-363-3p | TOR4A | 0.000733419 | 1.12E-14 |
| LINC00900 | hsa-miR-7-5p | GLDN | 0.00185352 | 2.46E-24 |
| LINC00900 | hsa-miR-7-5p, hsa-miR-363-3p | PMEPA1 | 0.000847383 | 3.06E-07 |
| LINC00900 | hsa-miR-214-3p | TRIM29 | 0.004431612 | 0.005551702 |
| LINC00900 | hsa-miR-150-5p | NOTCH3 | 0.002993486 | 3.15E-09 |
| LINC00900 | hsa-miR-181a-5p | S100A1 | 0.006949311 | 1.44E-07 |
| LINC00900 | hsa-miR-214-3p | RAB15 | 0.004519961 | 5.08E-05 |
| LINC00900 | hsa-miR-150-5p, hsa-miR-214-3p | TNFSF15 | 0.033842275 | 1.32E-06 |
| LINC00900 | hsa-miR-7-5p | SSX1 | 0.035013467 | 0.01610757 |
| LINC00900 | hsa-miR-7-5p, hsa-miR-33b-5p | TMEM98 | 0.030749313 | 3.48E-13 |
| LINC00900 | hsa-miR-150-5p | PLXDC1 | 0.004431612 | 2.38E-14 |
| LINC00900 | hsa-miR-204-5p | ALPL | 0.035013467 | 1.75E-08 |
| LINC00900 | hsa-miR-214-3p | ASF1B | 0.037191841 | 4.41E-05 |
| LINC00900 | hsa-miR-181a-5p, hsa-miR-181b-5p, hsa-miR-181d-5p | SCD | 0.021066655 | 7.49E-09 |
| LINC00900 | hsa-miR-204-5p | PLAUR | 0.016593637 | 0.000456433 |
| LINC00900 | hsa-miR-150-5p | C3orf36 | 0.000379356 | 5.68E-05 |
| LINC00900 | hsa-miR-150-5p | PTPRR | 0.035013467 | 0.013174065 |
| AC004847.1 | hsa-miR-7-5p | TRIM47 | 0.018083878 | 4.67E-45 |
| AC004847.1 | hsa-miR-7-5p | FNDC4 | 0.035847565 | 1.09E-54 |
| AC004847.1 | hsa-miR-363-3p | PLXNA3 | 0.002587602 | 2.17E-35 |
| AC004847.1 | hsa-miR-7-5p | BAX | 0.000110856 | 1.08E-29 |
| AC004847.1 | hsa-miR-7-5p | SSX1 | 0.018083878 | 3.85E-32 |
| AC004847.1 | hsa-miR-205-5p | RUNX2 | 0.013230131 | 4.00E-74 |
| AC004847.1 | hsa-miR-7-5p | LAMC2 | 0.008363377 | 5.81E-40 |
| AC004847.1 | hsa-miR-150-5p | PLXDC1 | 0.000368161 | 6.03E-18 |
| AC004847.1 | hsa-miR-150-5p | MMP14 | 0.015869925 | 8.14E-82 |
| AC004847.1 | hsa-miR-20b-5p | SCD | 0.000249694 | 3.29E-67 |
| AC004847.1 | hsa-miR-7-5p | DSC2 | 0.0063454 | 4.98E-57 |
| AC004847.1 | hsa-miR-20b-5p | PMAIP1 | 0.004134325 | 1.80E-69 |
| AC026369.2 | hsa-miR-214-3p | ENTPD1 | 0.022259645 | 2.72E-08 |
| AC026369.2 | hsa-miR-214-3p | JAG2 | 0.001073811 | 1.74E-30 |
| AC026369.2 | hsa-miR-138-5p | CDH2 | 0.021543354 | 6.24E-14 |
| AC026369.2 | hsa-miR-181a-5p, hsa-miR-214-3p | BAX | 0.008640814 | 0.004960854 |
| AC026369.2 | hsa-miR-214-3p | KCNK5 | 0.011747848 | 8.89E-08 |
| AC026369.2 | hsa-miR-138-5p, hsa-miR-214-3p | FAM109A | 0.041302387 | 1.96E-06 |
| AC026369.2 | hsa-miR-146b-5p | RARB | 0.001975718 | 0.001558031 |
| AC026369.2 | hsa-miR-181a-5p, hsa-miR-181b-5p | PBX3 | 0.000211179 | 7.66E-14 |
| AC090204.1 | hsa-miR-199b-5p | C1orf226 | 0.039333671 | 6.14E-17 |
| AC090204.1 | hsa-miR-363-3p | SOX4 | 0.00163652 | 1.87E-25 |
| AC090204.1 | hsa-miR-363-3p | PMEPA1 | 0.000121395 | 2.95E-09 |
| AC091563.1 | hsa-miR-221-3p | TUB | 0.002140023 | 1.76E-42 |
| AC091563.1 | hsa-miR-221-3p | PRDM16 | 0.029286639 | 5.90E-53 |
| AC091563.1 | hsa-miR-221-3p | SPAG5 | 0.020999639 | 4.04E-40 |
| AC091563.1 | hsa-miR-221-3p, hsa-miR-222-3p | KIT | 0.004634096 | 2.54E-83 |
| AC091563.1 | hsa-miR-221-3p | MEOX2 | 0.049725895 | 1.77E-12 |
| AC091563.1 | hsa-miR-221-3p, hsa-miR-222-3p | FOS | 0.023657978 | 6.77E-23 |
| AC091563.1 | hsa-miR-221-3p | ERBB4 | 0.037509819 | 1.35E-08 |
| ATP6V0E2-AS1 | hsa-miR-204-5p, hsa-miR-34a-5p | TPPP | 0.044688209 | 1.05E-38 |
| ATP6V0E2-AS1 | hsa-miR-34a-5p | CYTH3 | 0.000256728 | 1.47E-43 |
| ATP6V0E2-AS1 | hsa-miR-96-5p, hsa-miR-205-5p | SLC25A25 | 0.00426497 | 3.81E-11 |
| ATP6V0E2-AS1 | hsa-miR-96-5p | SOX5 | 0.005450893 | 9.69E-16 |
| ATP6V0E2-AS1 | hsa-miR-375 | SLC7A6 | 0.009244472 | 3.97E-28 |
| ATP6V0E2-AS1 | hsa-miR-205-5p | CYR61 | 0.01877272 | 2.63E-12 |
| ATP6V0E2-AS1 | hsa-miR-96-5p, hsa-miR-34a-5p | CDON | 0.030251708 | 4.72E-58 |
| ATP6V0E2-AS1 | hsa-miR-9-5p, hsa-miR-205-5p | SLC39A14 | 0.000477496 | 4.49E-52 |
| ATP6V0E2-AS1 | hsa-miR-146b-5p, hsa-miR-199b-5p, hsa-miR-34a-5p | KIT | 0.007024052 | 1.60E-51 |
| ATP6V0E2-AS1 | hsa-miR-7-5p, hsa-miR-34a-5p | FOS | 0.03424464 | 9.99E-13 |
| ATP6V0E2-AS1 | hsa-miR-187-3p | FOXA2 | 0.006888916 | 1.41E-18 |
| ATP6V0E2-AS1 | hsa-miR-375 | NCAM1 | 0.000967182 | 7.47E-49 |
| ATP6V0E2-AS1 | hsa-miR-34a-5p | GAS1 | 0.022906604 | 6.87E-09 |
| AL121839.2 | hsa-miR-21-5p | SOX5 | 0.001834545 | 2.71E-24 |
| AL121839.2 | hsa-miR-21-5p | TNFRSF11B | 0.003469789 | 3.43E-43 |
| AL121839.2 | hsa-miR-21-5p | FMOD | 0.000597139 | 1.87E-38 |
| AL121839.2 | hsa-miR-21-5p | FAXDC2 | 0.000597139 | 6.46E-17 |
| AL121839.2 | hsa-miR-199b-5p | KIT | 0.022259645 | 2.10E-38 |
| AL121839.2 | hsa-miR-21-5p | CCR7 | 0.003469789 | 0.005279677 |
| AL121839.2 | hsa-miR-21-5p | SOD3 | 0.024624856 | 1.27E-48 |
| AL121839.2 | hsa-miR-20b-5p | RUNX3 | 0.000888841 | 0.012108628 |
| AC006058.1 | hsa-miR-21-5p, hsa-miR-375 | TNS3 | 0.034612693 | 9.30E-21 |
| AC006058.1 | hsa-miR-34a-5p | CYTH3 | 0.001108674 | 6.55E-19 |
| AC006058.1 | hsa-miR-144-3p, hsa-miR-181a-5p, hsa-miR-181b-5p, hsa-miR-181d-5p, hsa-miR-375 | ZFP36L2 | 0.000836499 | 9.56E-45 |
| AC006058.1 | hsa-miR-96-5p, hsa-miR-181a-5p, hsa-miR-21-5p | SOX5 | 0.000204513 | 2.37E-20 |
| AC006058.1 | hsa-miR-144-3p, hsa-miR-375 | ELL2 | 0.010915483 | 0.002333291 |
| AC006058.1 | hsa-miR-9-5p, hsa-miR-181a-5p, hsa-miR-181b-5p, hsa-miR-181d-5p | ID4 | 0.022593637 | 4.01E-46 |
| AC006058.1 | hsa-miR-181a-5p, hsa-miR-181b-5p, hsa-miR-181d-5p | WDR72 | 0.003324226 | 2.17E-22 |
| AC006058.1 | hsa-miR-21-5p | FMOD | 0.002114866 | 6.94E-27 |
| AC006058.1 | hsa-miR-96-5p | PRDM16 | 0.000135911 | 5.85E-42 |
| AC006058.1 | hsa-miR-181a-5p, hsa-miR-181b-5p, hsa-miR-181d-5p | RGS16 | 0.000580399 | 7.59E-18 |
| AC006058.1 | hsa-miR-96-5p, hsa-miR-34a-5p | CDON | 0.016570288 | 1.03E-42 |
| AC006058.1 | hsa-miR-21-5p | FAXDC2 | 0.002114866 | 1.68E-28 |
| AC006058.1 | hsa-miR-181a-5p, hsa-miR-181b-5p, hsa-miR-181d-5p | ZNF781 | 0.006878339 | 1.24E-26 |
| AC006058.1 | hsa-miR-9-5p, hsa-miR-205-5p | SLC39A14 | 0.000347193 | 2.28E-08 |
| AC006058.1 | hsa-miR-181a-5p | ALDH1A1 | 0.002114866 | 7.28E-22 |
| AC006058.1 | hsa-miR-9-5p, hsa-miR-181a-5p | KLF6 | 0.001804751 | 0.000862969 |
| AC006058.1 | hsa-miR-181a-5p, hsa-miR-181b-5p, hsa-miR-34a-5p | FOS | 0.000240274 | 6.46E-23 |
| AC006058.1 | hsa-miR-181a-5p | GPR83 | 0.00615241 | 2.73E-24 |
| AC006058.1 | hsa-miR-375 | NCAM1 | 0.038124428 | 1.25E-09 |
| AC006058.1 | hsa-miR-21-5p | SOD3 | 0.046171604 | 1.44E-26 |
| AC006058.1 | hsa-miR-183-5p, hsa-miR-33b-5p, hsa-miR-34a-5p | GAS1 | 0.000134774 | 5.67E-06 |
| AC006058.1 | hsa-miR-146b-5p, hsa-miR-181a-5p, hsa-miR-34a-5p | PDGFRA | 0.001604185 | 0.023153413 |
| LINC01508 | hsa-miR-204-5p | TPPP | 0.017671439 | 1.71E-46 |
| LINC01508 | hsa-miR-204-5p, hsa-miR-205-5p | C11orf74 | 0.038690095 | 2.55E-61 |
| LINC01508 | hsa-miR-204-5p, hsa-miR-205-5p | NOX5 | 0.005926603 | 0.000974478 |
| LINC01508 | hsa-miR-205-5p | SLC39A14 | 0.00265899 | 3.92E-38 |
| LINC01508 | hsa-miR-199b-5p | KIT | 0.041302387 | 7.76E-63 |
| AC010210.1 | hsa-miR-34a-5p | CYTH3 | 0.000716845 | 2.87E-12 |
| AC010210.1 | hsa-miR-503-5p | KIAA1456 | 0.008321085 | 8.61E-61 |
| AC010210.1 | hsa-miR-146b-5p, hsa-miR-199b-5p, hsa-miR-34a-5p | KIT | 0.000395469 | 1.25E-22 |
| AC010210.1 | hsa-miR-7-5p | RGS5 | 0.048711977 | 0.003253056 |
| GUSBP11 | hsa-miR-150-5p, hsa-miR-20b-5p, hsa-miR-204-5p | FAXC | 0.001917121 | 1.16E-28 |
| GUSBP11 | hsa-miR-144-3p | MET | 0.001644608 | 9.70E-20 |
| GUSBP11 | hsa-miR-204-5p | BID | 0.047546542 | 6.03E-39 |
| GUSBP11 | hsa-miR-204-5p | SAMD1 | 0.006764646 | 6.42E-30 |
| GUSBP11 | hsa-miR-138-5p | LCN2 | 0.041554444 | 6.88E-13 |
| GUSBP11 | hsa-miR-150-5p, hsa-miR-205-5p, hsa-miR-375 | XPR1 | 0.018172304 | 2.27E-06 |
| GUSBP11 | hsa-miR-214-3p | AHNAK2 | 0.026328109 | 2.05E-16 |
| GUSBP11 | hsa-miR-20b-5p | LIMK1 | 0.005016575 | 2.40E-07 |
| GUSBP11 | hsa-miR-181a-5p | RUNX1 | 0.001498294 | 1.42E-23 |
| GUSBP11 | hsa-miR-204-5p, hsa-miR-375 | IL1RAP | 0.000742771 | 3.49E-18 |
| GUSBP11 | hsa-miR-138-5p | LPL | 0.006764646 | 1.06E-10 |
| GUSBP11 | hsa-miR-204-5p | CD44 | 0.000492877 | 9.42E-12 |
| GUSBP11 | hsa-miR-150-5p | MAPK13 | 0.023660603 | 1.53E-07 |
| GUSBP11 | hsa-miR-214-3p | LZTS1 | 0.001711442 | 2.05E-09 |
| GUSBP11 | hsa-miR-214-3p | TRIM29 | 0.008163312 | 1.12E-12 |
| GUSBP11 | hsa-miR-138-5p, hsa-miR-181a-5p | S100A1 | 0.009718288 | 5.23E-13 |
| GUSBP11 | hsa-miR-204-5p | PI4K2A | 0.00902285 | 2.74E-05 |
| GUSBP11 | hsa-miR-199b-5p | LAMC2 | 0.000173645 | 0.027955475 |
| GUSBP11 | hsa-miR-150-5p | PLXDC1 | 0.008163312 | 3.92E-20 |
| GUSBP11 | hsa-miR-375 | TGM2 | 0.031337754 | 4.22E-05 |
| GUSBP11 | hsa-miR-204-5p | CREB5 | 0.004174427 | 4.85E-25 |
| GUSBP11 | hsa-miR-20b-5p | CTSS | 0.000194128 | 1.68E-09 |
| GUSBP11 | hsa-miR-204-5p, hsa-miR-375 | ARNTL2 | 0.001062396 | 0.0061286 |
| AC124312.3 | hsa-miR-144-3p, hsa-miR-375 | ELL2 | 0.00388923 | 4.33E-14 |
| AC124312.3 | hsa-miR-181a-5p | EGR1 | 0.003927478 | 2.84E-19 |
| AC124312.3 | hsa-miR-181a-5p, hsa-miR-181b-5p, hsa-miR-181d-5p | RGS16 | 0.000169399 | 3.92E-06 |
| AC124312.3 | hsa-miR-181a-5p | MOB3B | 0.024307678 | 6.93E-09 |
| AC124312.3 | hsa-miR-146b-5p | RARB | 0.004058993 | 4.80E-23 |
| AC124312.3 | hsa-miR-9-5p, hsa-miR-205-5p | SLC39A14 | 0.00222832 | 7.11E-17 |
| AC124312.3 | hsa-miR-181a-5p | ALDH1A1 | 0.024477941 | 6.71E-09 |
| AC124312.3 | hsa-miR-181a-5p | OCA2 | 0.00897717 | 6.80E-14 |
| AC124312.3 | hsa-miR-146b-5p | KIT | 0.003245509 | 8.22E-25 |
| AC124312.3 | hsa-miR-205-5p, hsa-miR-375 | CTGF | 0.005494558 | 3.14E-15 |
| AC124312.3 | hsa-miR-182-5p | TCEAL7 | 0.024477941 | 5.56E-08 |
| AC124312.3 | hsa-miR-181a-5p | AKAP12 | 0.026662103 | 2.59E-35 |
| AC124312.3 | hsa-miR-9-5p, hsa-miR-181a-5p | KLF6 | 0.00640237 | 6.60E-07 |
| AC124312.3 | hsa-miR-181a-5p, hsa-miR-181b-5p | FOS | 0.033086601 | 1.46E-11 |
| AC124312.3 | hsa-miR-181a-5p | GPR83 | 0.000437351 | 2.60E-11 |
| DGUOK-AS1 | hsa-miR-204-5p | HMGA2 | 0.003345955 | 4.21E-16 |
| DGUOK-AS1 | hsa-miR-214-3p | GALNT7 | 0.032586419 | 8.46E-08 |
| DGUOK-AS1 | hsa-miR-138-5p | CCND1 | 0.000102232 | 5.25E-06 |
| DGUOK-AS1 | hsa-miR-204-5p | BID | 0.011853229 | 2.19E-06 |
| DGUOK-AS1 | hsa-miR-214-3p | JAG2 | 0.000730941 | 1.75E-17 |
| DGUOK-AS1 | hsa-miR-204-5p | SAMD1 | 0.002954935 | 3.78E-17 |
| DGUOK-AS1 | hsa-miR-204-5p | SH2D4A | 0.008615666 | 0.001159457 |
| DGUOK-AS1 | hsa-miR-204-5p | MDFI | 0.036224181 | 5.07E-11 |
| DGUOK-AS1 | hsa-miR-204-5p | FSTL4 | 0.045967233 | 0.000662903 |
| DGUOK-AS1 | hsa-miR-214-3p | BAX | 0.000224435 | 5.96E-17 |
| DGUOK-AS1 | hsa-miR-214-3p | KCNK5 | 0.044042889 | 3.39E-26 |
| DGUOK-AS1 | hsa-miR-138-5p | S100A1 | 0.033450748 | 8.95E-10 |
| DGUOK-AS1 | hsa-miR-214-3p | TNFSF15 | 0.019966999 | 0.01650025 |
| DGUOK-AS1 | hsa-miR-204-5p | IGFBP2 | 0.033450748 | 0.000432093 |
| DGUOK-AS1 | hsa-miR-138-5p, hsa-miR-214-3p | FAM109A | 0.032586419 | 1.19E-20 |
| DGUOK-AS1 | hsa-miR-204-5p | ALPL | 0.008464794 | 2.43E-11 |
| DGUOK-AS1 | hsa-miR-204-5p | LRRC4 | 0.008615666 | 3.74E-06 |
| DGUOK-AS1 | hsa-miR-204-5p | NOX5 | 0.024191056 | 3.35E-09 |
| DGUOK-AS1 | hsa-miR-204-5p | FAM167B | 0.011853229 | 1.45E-35 |
| UBXN10-AS1 | hsa-miR-204-5p | FAXC | 0.016900624 | 6.59E-15 |
| UBXN10-AS1 | hsa-miR-551b-3p | PDE4C | 0.015479576 | 2.41E-29 |
| UBXN10-AS1 | hsa-miR-204-5p | SAMD1 | 0.00013247 | 1.57E-25 |
| UBXN10-AS1 | hsa-miR-204-5p | CDH4 | 0.000161783 | 1.71E-13 |
| UBXN10-AS1 | hsa-miR-204-5p | IL1RAP | 0.020999639 | 3.01E-10 |
| UBXN10-AS1 | hsa-miR-204-5p | SH2D4A | 0.000398197 | 3.24E-14 |
| UBXN10-AS1 | hsa-miR-204-5p | MDFI | 0.001817661 | 0.000129365 |
| UBXN10-AS1 | hsa-miR-204-5p | CD44 | 0.006785823 | 5.11E-26 |
| UBXN10-AS1 | hsa-miR-204-5p | FSTL4 | 0.002356686 | 0.006996544 |
| UBXN10-AS1 | hsa-miR-204-5p | SOX4 | 0.002409117 | 4.31E-06 |
| UBXN10-AS1 | hsa-miR-204-5p | CDH2 | 0.045360991 | 1.73E-05 |
| UBXN10-AS1 | hsa-miR-204-5p | PLAG1 | 0.009465322 | 7.82E-31 |
| UBXN10-AS1 | hsa-miR-204-5p | B4GALNT3 | 0.003092359 | 1.61E-12 |
| UBXN10-AS1 | hsa-miR-204-5p | PI4K2A | 0.000161783 | 9.32E-11 |
| UBXN10-AS1 | hsa-miR-204-5p | MEX3A | 0.012303044 | 0.001602868 |
| UBXN10-AS1 | hsa-miR-204-5p | IGFBP2 | 0.007677509 | 5.25E-07 |
| UBXN10-AS1 | hsa-miR-204-5p | ALPL | 0.001923817 | 3.26E-09 |
| UBXN10-AS1 | hsa-miR-204-5p | MYRF | 0.001177753 | 0.000206481 |
| UBXN10-AS1 | hsa-miR-204-5p | EPHA4 | 0.007565779 | 4.70E-07 |
| UBXN10-AS1 | hsa-miR-204-5p | FAM167B | 0.000555016 | 2.18E-11 |
| UBXN10-AS1 | hsa-miR-204-5p | PLAUR | 0.01149854 | 2.86E-12 |
| UBXN10-AS1 | hsa-miR-204-5p | CREB5 | 0.002025136 | 7.21E-11 |
| UBXN10-AS1 | hsa-miR-204-5p | STEAP4 | 0.000499901 | 0.023837476 |
| AC128688.2 | hsa-miR-144-3p | ZFP36L2 | 0.023253481 | 2.03E-13 |
| AC128688.2 | hsa-miR-204-5p | C11orf74 | 0.04474766 | 4.25E-40 |
| AC128688.2 | hsa-miR-21-5p | SOX5 | 0.013732175 | 1.15E-11 |
| AC128688.2 | hsa-miR-144-3p | ELL2 | 0.037346486 | 5.15E-12 |
| AC128688.2 | hsa-miR-21-5p | FMOD | 0.027516357 | 2.24E-23 |
| AC128688.2 | hsa-miR-21-5p | FAXDC2 | 0.027516357 | 4.15E-14 |
| AC128688.2 | hsa-miR-363-3p | MRO | 0.000640541 | 4.27E-28 |
| AC128688.2 | hsa-miR-144-3p | PBX3 | 0.01322251 | 0.000460423 |
| AC128688.2 | hsa-miR-21-5p | SOD3 | 0.013851481 | 5.27E-32 |
| AC128688.2 | hsa-miR-204-5p, hsa-miR-21-5p | SAMD5 | 0.00421659 | 1.61E-25 |
| AC128688.2 | hsa-miR-33b-5p | GAS1 | 0.011788225 | 0.000117128 |
| FZD10-AS1 | hsa-miR-7-5p | FNDC4 | 0.000616095 | 0.039545869 |
| FZD10-AS1 | hsa-miR-183-5p | SLC25A25 | 0.001522544 | 0.014649393 |
| FZD10-AS1 | hsa-miR-20b-5p | RTN2 | 0.000180077 | 0.012765427 |
| FZD10-AS1 | hsa-miR-20b-5p | SLC1A5 | 0.000262403 | 0.000374803 |
| FZD10-AS1 | hsa-miR-183-5p | EGR1 | 0.015649856 | 9.93E-07 |
| FZD10-AS1 | hsa-miR-20b-5p | PCNX2 | 0.001523567 | 0.004880939 |
| FZD10-AS1 | hsa-miR-182-5p | TCEAL7 | 0.049403143 | 2.12E-23 |
| FZD10-AS1 | hsa-miR-187-3p, hsa-miR-363-3p | MRO | 0.005672579 | 0.016134311 |
| FZD10-AS1 | hsa-miR-182-5p | CREB5 | 0.013895826 | 3.35E-06 |
| FZD10-AS1 | hsa-miR-187-3p | FOXA2 | 0.040230566 | 1.18E-07 |
| FZD10-AS1 | hsa-miR-20b-5p | RUNX3 | 0.000976633 | 3.03E-19 |
| FZD10-AS1 | hsa-miR-31-5p | SELE | 0.004463508 | 7.31E-21 |
| AP004608.1 | hsa-miR-150-5p | ZMAT3 | 0.006697138 | 0.000841066 |
| AP004608.1 | hsa-miR-204-5p | BID | 0.005575735 | 0.022795059 |
| AP004608.1 | hsa-miR-9-5p | NOX4 | 0.012809223 | 0.007577935 |
| AP004608.1 | hsa-miR-214-3p | JAG2 | 0.001037701 | 3.25E-12 |
| AP004608.1 | hsa-miR-138-5p | MTHFD1L | 0.026476744 | 6.40E-10 |
| AP004608.1 | hsa-miR-204-5p | CDH4 | 0.006686705 | 8.58E-07 |
| AP004608.1 | hsa-miR-204-5p | SH2D4A | 0.023427933 | 0.000508943 |
| AP004608.1 | hsa-miR-138-5p | LPL | 0.004997252 | 7.72E-07 |
| AP004608.1 | hsa-miR-9-5p | RAB34 | 0.000190954 | 3.10E-09 |
| AP004608.1 | hsa-miR-363-3p | PLXNA3 | 0.008237799 | 1.33E-14 |
| AP004608.1 | hsa-miR-138-5p, hsa-miR-204-5p, hsa-miR-363-3p, hsa-miR-31-5p | SOX4 | 0.008404918 | 0.021899444 |
| AP004608.1 | hsa-miR-363-3p | TOR4A | 0.001169992 | 1.15E-08 |
| AP004608.1 | hsa-miR-204-5p | B4GALNT3 | 0.028961935 | 1.49E-06 |
| AP004608.1 | hsa-miR-138-5p, hsa-miR-181a-5p | S100A1 | 0.007876525 | 7.77E-08 |
| AP004608.1 | hsa-miR-214-3p | RAB15 | 0.006904064 | 0.00555843 |
| AP004608.1 | hsa-miR-150-5p, hsa-miR-214-3p | TNFSF15 | 0.044247843 | 7.16E-07 |
| AP004608.1 | hsa-miR-199b-5p | LAMC2 | 0.033333983 | 0.001597465 |
| AP004608.1 | hsa-miR-150-5p | PLXDC1 | 0.005575735 | 0.010887077 |
| AP004608.1 | hsa-miR-9-5p, hsa-miR-375 | FSTL3 | 0.013734986 | 8.87E-07 |
| AP004608.1 | hsa-miR-214-3p | ASF1B | 0.003594768 | 0.016165491 |
| AP004608.1 | hsa-miR-96-5p, hsa-miR-138-5p, hsa-miR-182-5p | CASTOR2 | 0.000270128 | 0.003511824 |
| AP004608.1 | hsa-miR-9-5p | NTRK3 | 0.009352838 | 4.81E-05 |
| AP004608.1 | hsa-miR-9-5p, hsa-miR-205-5p | SLC7A2 | 0.024453014 | 0.000448314 |
| LINC01550 | hsa-miR-34a-5p | CYTH3 | 0.000619601 | 6.33E-41 |
| LINC01550 | hsa-miR-375 | PRKX | 0.047249006 | 1.36E-07 |
| LINC01550 | hsa-miR-96-5p | SLC25A25 | 0.047449641 | 2.38E-10 |
| LINC01550 | hsa-miR-96-5p | SOX5 | 0.014079895 | 2.07E-10 |
| LINC01550 | hsa-miR-375 | SLC7A6 | 0.014383738 | 1.88E-39 |
| LINC01550 | hsa-miR-96-5p, hsa-miR-221-3p | PRDM16 | 0.028676234 | 1.71E-31 |
| LINC01550 | hsa-miR-96-5p, hsa-miR-34a-5p | CDON | 0.009809516 | 2.06E-80 |
| LINC01550 | hsa-miR-375 | CTGF | 0.000915786 | 0.000261185 |
| LINC01550 | hsa-miR-221-3p | MEOX2 | 0.010244131 | 1.57E-18 |
| LINC01550 | hsa-miR-150-5p, hsa-miR-199b-5p | C3orf36 | 0.00020318 | 0.030216761 |
| LINC01550 | hsa-miR-221-3p, hsa-miR-222-3p, hsa-miR-34a-5p | FOS | 0.000357457 | 6.88E-12 |
| LINC01550 | hsa-miR-144-3p | PBX3 | 0.001262387 | 0.00219132 |
| LINC01550 | hsa-miR-375 | NCAM1 | 0.028676234 | 4.45E-39 |
| LINC01550 | hsa-miR-221-3p | CXCL12 | 0.00690499 | 2.28E-05 |
| LINC01550 | hsa-miR-221-3p, hsa-miR-222-3p | SELE | 0.015999949 | 0.000999488 |
| AL590094.1 | hsa-miR-34a-5p | CYTH3 | 0.000337787 | 1.58E-21 |
| AL590094.1 | hsa-miR-96-5p | SOX5 | 0.000180752 | 6.15E-22 |
| AL590094.1 | hsa-miR-503-5p | KIAA1456 | 0.005053941 | 1.66E-28 |
| AL590094.1 | hsa-miR-182-5p, hsa-miR-31-5p | SYDE2 | 0.040722737 | 0.000208978 |
| AL590094.1 | hsa-miR-96-5p, hsa-miR-221-3p | PRDM16 | 0.001190006 | 2.14E-06 |
| AL590094.1 | hsa-miR-221-3p | MEOX2 | 0.006612622 | 1.63E-06 |
| AL590094.1 | hsa-miR-96-5p, hsa-miR-182-5p | RGS2 | 0.021180467 | 0.000282611 |
| AL590094.1 | hsa-miR-375 | NCAM1 | 0.021308352 | 5.51E-33 |
| BCRP3 | hsa-miR-214-3p, hsa-miR-34a-5p | GALNT7 | 0.000346599 | 1.49E-23 |
| BCRP3 | hsa-miR-503-5p | ZMAT3 | 0.00050303 | 0.000946955 |
| BCRP3 | hsa-miR-34a-5p | CD44 | 0.005303689 | 1.28E-18 |
| BCRP3 | hsa-miR-214-3p | DOCK9 | 0.007556311 | 6.14E-12 |
| BCRP3 | hsa-miR-214-3p | LZTS1 | 0.020673256 | 5.28E-08 |
| BCRP3 | hsa-miR-205-5p | RUNX2 | 0.000159701 | 9.88E-32 |
| BCRP3 | hsa-miR-214-3p | PAPPA | 0.00086775 | 9.19E-05 |
| BCRP3 | hsa-miR-34a-5p | PDGFRA | 0.024109595 | 0.000627027 |
| BCRP3 | hsa-miR-34a-5p | ZAP70 | 0.000615818 | 0.002228143 |
| LINC00982 | hsa-miR-204-5p, hsa-miR-34a-5p | TPPP | 0.012015228 | 6.24E-32 |
| LINC00982 | hsa-miR-183-5p | SLC25A25 | 0.036404137 | 0.004695352 |
| LINC00982 | hsa-miR-183-5p | EGR1 | 0.011347574 | 5.83E-19 |
| LINC00982 | hsa-miR-199b-5p, hsa-miR-34a-5p | KIT | 0.001632697 | 3.64E-41 |
| LINC00982 | hsa-miR-34a-5p | FOS | 0.000168004 | 2.87E-20 |
| PTCSC3 | hsa-miR-199b-5p | TSC22D1 | 0.006129758 | 0.005004729 |
| PTCSC3 | hsa-miR-96-5p | SOX5 | 0.034945168 | 5.90E-08 |
| PTCSC3 | hsa-miR-146b-5p | RARB | 0.019330314 | 1.72E-07 |
| PTCSC3 | hsa-miR-96-5p | CDON | 0.013034121 | 0.000161821 |
| PTCSC3 | hsa-miR-146b-5p, hsa-miR-199b-5p | KIT | 0.016682687 | 2.43E-15 |
| PTCSC3 | hsa-miR-205-5p | CTGF | 0.001605134 | 5.25E-06 |
| PTCSC3 | hsa-miR-182-5p | TCEAL7 | 0.01609786 | 6.58E-08 |
| PTCSC3 | hsa-miR-199b-5p | SNAP25 | 0.016682687 | 0.00565537 |
| PTCSC3 | hsa-miR-182-5p | CREB5 | 0.036708835 | 0.040248453 |
| PTCSC3 | hsa-miR-96-5p, hsa-miR-182-5p | RGS2 | 0.000343416 | 0.004751971 |
| AC020659.1 | hsa-miR-33b-5p, hsa-miR-34a-5p | HMGA2 | 0.000117645 | 0.000251338 |
| AC020659.1 | hsa-miR-9-5p | P4HA2 | 0.038868209 | 0.005974856 |
| AC020659.1 | hsa-miR-214-3p, hsa-miR-34a-5p | GALNT7 | 0.003951598 | 0.031621076 |
| AC020659.1 | hsa-miR-199b-5p | PLXND1 | 0.039481541 | 0.045110773 |
| AC020659.1 | hsa-miR-551b-3p | PDE4C | 0.016861872 | 2.63E-08 |
| AC020659.1 | hsa-miR-214-3p | JAG2 | 0.008429335 | 2.50E-13 |
| AC020659.1 | hsa-miR-7-5p | TRIM47 | 0.019622932 | 1.41E-07 |
| AC020659.1 | hsa-miR-7-5p | FNDC4 | 0.038868209 | 4.43E-05 |
| AC020659.1 | hsa-miR-34a-5p | CD44 | 0.046619914 | 0.026103242 |
| AC020659.1 | hsa-miR-7-5p, hsa-miR-214-3p, hsa-miR-34a-5p | BAX | 0.002743553 | 3.85E-09 |
| AC020659.1 | hsa-miR-214-3p | ASF1B | 0.000550514 | 0.000208616 |
| AC020659.1 | hsa-miR-9-5p, hsa-miR-375 | SDC1 | 0.003330222 | 0.028309825 |
| AC020659.1 | hsa-miR-9-5p | NTRK3 | 0.009561016 | 3.15E-05 |
| LINC01571 | hsa-miR-204-5p | TPPP | 0.034596364 | 2.34E-11 |
| LINC01571 | hsa-miR-182-5p, hsa-miR-31-5p | SYDE2 | 0.010518354 | 1.81E-15 |
| LINC01571 | hsa-miR-96-5p | PRDM16 | 0.047538211 | 4.81E-13 |
| LINC01571 | hsa-miR-204-5p | FAM167B | 0.007996853 | 0.00018143 |
| LINC01571 | hsa-miR-182-5p | TCEAL7 | 0.013806163 | 1.54E-18 |
| LINC01571 | hsa-miR-204-5p | SAMD5 | 0.043968457 | 2.59E-10 |
| LINC01571 | hsa-miR-31-5p | SELE | 0.003925502 | 0.001792455 |
| AC015914.1 | hsa-miR-9-5p | CDH3 | 0.021779893 | 3.38E-34 |
| AC015914.1 | hsa-miR-9-5p | P4HA2 | 0.014570358 | 4.96E-09 |
| AC015914.1 | hsa-miR-9-5p, hsa-miR-138-5p, hsa-miR-183-5p | CCND1 | 0.030296888 | 9.05E-25 |
| AC015914.1 | hsa-miR-9-5p | KCNJ2 | 0.017094391 | 7.48E-19 |
| AC015914.1 | hsa-miR-150-5p | TMEM92 | 0.00176847 | 2.01E-27 |
| AC015914.1 | hsa-miR-9-5p | NOX4 | 0.036049324 | 3.72E-15 |
| AC015914.1 | hsa-miR-214-3p | JAG2 | 0.000467572 | 0.000261417 |
| AC015914.1 | hsa-miR-138-5p | MTHFD1L | 0.02075672 | 6.29E-19 |
| AC015914.1 | hsa-miR-138-5p | LCN2 | 0.007310504 | 1.11E-17 |
| AC015914.1 | hsa-miR-7-5p, hsa-miR-150-5p, hsa-miR-205-5p | XPR1 | 0.000688684 | 2.10E-24 |
| AC015914.1 | hsa-miR-214-3p | AHNAK2 | 0.000174819 | 6.80E-49 |
| AC015914.1 | hsa-miR-7-5p | TRIM47 | 0.007310504 | 6.79E-11 |
| AC015914.1 | hsa-miR-138-5p | SEMA4C | 0.000549822 | 5.55E-34 |
| AC015914.1 | hsa-miR-7-5p | RNF183 | 0.021779893 | 2.96E-20 |
| AC015914.1 | hsa-miR-138-5p | LPL | 0.002200958 | 6.97E-09 |
| AC015914.1 | hsa-miR-7-5p | FNDC4 | 0.014570358 | 1.20E-12 |
| AC015914.1 | hsa-miR-7-5p, hsa-miR-9-5p | ALDH1A3 | 0.02204465 | 2.51E-11 |
| AC015914.1 | hsa-miR-9-5p | GALNTL6 | 0.028939439 | 3.66E-28 |
| AC015914.1 | hsa-miR-150-5p, hsa-miR-214-3p | KCNK5 | 0.000454536 | 1.17E-11 |
| AC015914.1 | hsa-miR-214-3p | TRIM29 | 0.000349347 | 3.04E-23 |
| AC015914.1 | hsa-miR-138-5p | S100A1 | 0.028939439 | 2.04E-07 |
| AC015914.1 | hsa-miR-150-5p, hsa-miR-214-3p | TNFSF15 | 0.000106454 | 3.98E-36 |
| AC015914.1 | hsa-miR-7-5p | SSX1 | 0.007310504 | 3.10E-17 |
| AC015914.1 | hsa-miR-205-5p | RUNX2 | 0.017094391 | 5.91E-34 |
| AC015914.1 | hsa-miR-9-5p | RHOV | 0.002678346 | 0.002743088 |
| AC015914.1 | hsa-miR-150-5p | EMP2 | 0.021712544 | 1.59E-22 |
| AC015914.1 | hsa-miR-150-5p | MMP14 | 0.002678346 | 1.01E-06 |
| AC015914.1 | hsa-miR-9-5p | NTRK3 | 0.000535223 | 1.36E-08 |
| AC015914.1 | hsa-miR-9-5p, hsa-miR-205-5p | SLC7A2 | 0.003872985 | 3.94E-08 |
| AC015914.1 | hsa-miR-7-5p, hsa-miR-214-3p | PAPPA | 0.008041839 | 1.05E-13 |
| AC015914.1 | hsa-miR-9-5p | ESR1 | 0.01436578 | 2.49E-06 |
| AC005082.1 | hsa-miR-199b-5p | KIT | 0.016682687 | 9.53E-08 |
| AC005082.1 | hsa-miR-150-5p | CYTIP | 0.033216347 | 0.001216594 |
| SMIM25 | hsa-miR-33b-5p, hsa-miR-34a-5p | HMGA2 | 0.001061205 | 9.26E-15 |
| SMIM25 | hsa-miR-214-3p, hsa-miR-34a-5p | GALNT7 | 0.005197175 | 9.52E-13 |
| SMIM25 | hsa-miR-20b-5p | DRAXIN | 0.00017824 | 3.33E-29 |
| SMIM25 | hsa-miR-20b-5p | PDE4C | 0.006528934 | 7.00E-24 |
| SMIM25 | hsa-miR-20b-5p | MANEAL | 0.000459829 | 0.000359071 |
| SMIM25 | hsa-miR-214-3p | JAG2 | 0.010388809 | 0.003270731 |
| SMIM25 | hsa-miR-7-5p | TRIM47 | 0.021161985 | 1.27E-39 |
| SMIM25 | hsa-miR-20b-5p | PSD3 | 0.029216058 | 4.72E-11 |
| SMIM25 | hsa-miR-7-5p | FNDC4 | 0.041884114 | 5.00E-32 |
| SMIM25 | hsa-miR-34a-5p | CD44 | 0.003144592 | 0.002988444 |
| SMIM25 | hsa-miR-20b-5p | HIP1 | 0.000773659 | 0.012075714 |
| SMIM25 | hsa-miR-214-3p | LZTS1 | 0.000439857 | 2.70E-09 |
| SMIM25 | hsa-miR-214-3p | TRIM29 | 0.000676532 | 3.06E-29 |
| SMIM25 | hsa-miR-214-3p | RAB15 | 0.002983314 | 3.03E-05 |
| SMIM25 | hsa-miR-7-5p | SSX1 | 0.021161985 | 1.28E-11 |
| SMIM25 | hsa-miR-7-5p, hsa-miR-33b-5p | TMEM98 | 0.007923625 | 1.12E-21 |
| SMIM25 | hsa-miR-20b-5p | RTN2 | 0.000381702 | 3.28E-21 |
| SMIM25 | hsa-miR-7-5p | LAMC2 | 0.011347574 | 4.11E-38 |
| SMIM25 | hsa-miR-7-5p, hsa-miR-34a-5p | TGM2 | 0.008627206 | 2.09E-61 |
| SMIM25 | hsa-miR-20b-5p, hsa-miR-34a-5p | SLC1A5 | 0.000789333 | 1.34E-36 |
| SMIM25 | hsa-miR-20b-5p | PCNX2 | 0.000550275 | 1.09E-09 |
| SMIM25 | hsa-miR-20b-5p | EPHA4 | 0.000122331 | 3.83E-24 |
| SMIM25 | hsa-miR-20b-5p | SCD | 0.003733865 | 9.04E-79 |
| SMIM25 | hsa-miR-7-5p | DSC2 | 0.008627206 | 5.59E-42 |
| SMIM25 | hsa-miR-20b-5p | PMAIP1 | 0.033141468 | 1.17E-47 |
| SMIM25 | hsa-miR-7-5p | ROR1 | 0.011347574 | 3.05E-27 |
| SMIM25 | hsa-miR-31-5p | CXCL12 | 0.000676532 | 2.12E-07 |
| SMIM25 | hsa-miR-7-5p, hsa-miR-214-3p | PAPPA | 0.000549822 | 1.70E-22 |
| SMIM25 | hsa-miR-34a-5p | PDGFRA | 0.008527332 | 2.26E-17 |
| SMIM25 | hsa-miR-34a-5p | ZAP70 | 0.002567865 | 1.17E-30 |
| SMIM25 | hsa-miR-31-5p | SELE | 0.034004598 | 0.001990097 |
| LINC00958 | hsa-miR-204-5p, hsa-miR-33b-5p | HMGA2 | 0.022191774 | 7.40E-07 |
| LINC00958 | hsa-miR-150-5p, hsa-miR-20b-5p, hsa-miR-204-5p | FAXC | 0.000156248 | 3.07E-05 |
| LINC00958 | hsa-miR-204-5p | BID | 0.009627458 | 0.005999519 |
| LINC00958 | hsa-miR-204-5p | SAMD1 | 0.02028243 | 0.004865739 |
| LINC00958 | hsa-miR-214-3p | AHNAK2 | 0.000355855 | 4.36E-05 |
| LINC00958 | hsa-miR-7-5p | TRIM47 | 0.022701039 | 0.022306568 |
| LINC00958 | hsa-miR-20b-5p | LIMK1 | 0.000130384 | 0.001340918 |
| LINC00958 | hsa-miR-204-5p | SH2D4A | 0.006026995 | 1.73E-07 |
| LINC00958 | hsa-miR-7-5p | FNDC4 | 0.04489528 | 3.92E-07 |
| LINC00958 | hsa-miR-204-5p | CD44 | 0.004490358 | 5.73E-05 |
| LINC00958 | hsa-miR-204-5p | TGFBR1 | 0.000114852 | 0.013443897 |
| LINC00958 | hsa-miR-204-5p | PLAG1 | 0.038197124 | 3.33E-06 |
| LINC00958 | hsa-miR-214-3p | TRIM29 | 0.00088503 | 3.35E-05 |
| LINC00958 | hsa-miR-7-5p | SSX1 | 0.022701039 | 0.005265463 |
| LINC00958 | hsa-miR-7-5p, hsa-miR-33b-5p | TMEM98 | 0.009627458 | 0.00043771 |
| LINC00958 | hsa-miR-204-5p | RUNX2 | 0.000334489 | 0.022317558 |
| LINC00958 | hsa-miR-7-5p | LAMC2 | 0.012993778 | 0.000104315 |
| LINC00958 | hsa-miR-150-5p | MMP14 | 0.024431541 | 0.002499184 |
| LINC00958 | hsa-miR-96-5p, hsa-miR-20b-5p, hsa-miR-182-5p | CDKN1A | 0.000225413 | 0.000533336 |
| LINC00958 | hsa-miR-204-5p | STEAP4 | 0.008316094 | 0.000674998 |
| PROSER2-AS1 | hsa-miR-375 | ZFP36L2 | 0.027191887 | 1.66E-07 |
| PROSER2-AS1 | hsa-miR-96-5p | SOX5 | 0.000290218 | 3.90E-08 |
| PROSER2-AS1 | hsa-miR-9-5p | ID4 | 0.027995805 | 2.73E-16 |
| PROSER2-AS1 | hsa-miR-375 | SLC7A6 | 0.00622717 | 1.99E-07 |
| PROSER2-AS1 | hsa-miR-96-5p | PRDM16 | 0.000532486 | 3.26E-22 |
| PROSER2-AS1 | hsa-miR-205-5p | CYR61 | 0.009404246 | 4.17E-14 |
| PROSER2-AS1 | hsa-miR-7-5p, hsa-miR-375 | SLC25A15 | 0.000989773 | 8.40E-08 |
| PROSER2-AS1 | hsa-miR-187-3p, hsa-miR-363-3p | MRO | 0.006827725 | 3.97E-24 |
| PROSER2-AS1 | hsa-miR-150-5p | C3orf36 | 0.001285336 | 0.002207614 |
| PROSER2-AS1 | hsa-miR-150-5p | PTPRR | 0.025779146 | 0.036831379 |
| PROSER2-AS1 | hsa-miR-375 | NCAM1 | 0.012647067 | 0.000323962 |
| PROSER2-AS1 | hsa-miR-363-3p | GOLGA8A | 0.000591712 | 0.033416294 |
| Abbreviations: ceRNAs: Competing endogenous RNAs; LncRNA: Long non-coding RNA; miRNA: microRNA. | | | | |
